# Augmented reality-delivered product information at the point of sale: when information controllability backfires

**DOI:** 10.1007/s11747-022-00855-w

**Published:** 2022-04-07

**Authors:** Stefan Hoffmann, Tom Joerß, Robert Mai, Payam Akbar

**Affiliations:** 1 Institute of Business Administration, Department of Marketing, Kiel University, Westring 425, 24098 Kiel, Germany; 2 Grenoble Ecole de Management, Department of Marketing, 12 Rue Pierre Semard, 38000 Grenoble, France

**Keywords:** Augmented reality, Controllability, Point of sale, Field study, Product information

## Abstract

Augmented reality-delivered product information (ARPI) can overcome the limited space at the point of sale to inform shoppers on demand and will therefore become more widespread in brick-and-mortar stores. To fill the void of academic research, this paper develops a model of how consumers process ARPI and how ARPI can shape brand image and purchase intentions. Making use of the cues-filtered-out theory, this paper suggests that the effect of ARPI controllability depends on information detailedness. An unintended backfire effect of controllability occurs when the accessible information is detailed, which is explained by the mediating process via perceived comprehensiveness. This backfire effect is a risk primarily in busy shopping times. The main experiment conducted in a hypermarket and four follow-up studies (using field, lab, and video settings) empirically confirm the proposed model on the basis of different data sources, including usage tracking, questionnaires, and scanner data. The paper derives managerial implications and outlines directions for future research.

**Supplementary Information:**

The online version contains supplementary material available at 10.1007/s11747-022-00855-w.

## Introduction

The lines between the physical world and the digital world are blurred. The same now becomes true for traditional brick-and-mortar stores, where augmented reality (AR) systems set out to change the shopping experience. On digital devices, such as mobile phones, tablets, AR smart glasses, AR technology provides an image of the physical environment that is enhanced by virtual elements (Azuma et al., 2001; Flavián et al., 2019). These elements overlay the physical environment such that they appear to coexist within the real world, and users can even interact with them in real time (Javornik, 2016a; Zhou et al., 2008). For grocery shopping, AR apps that inform consumers about product details have become popular. Chiquita partnered with Shazam to create an AR app that enables consumers to gather information about farming and harvesting when scanning the blue sticker on bananas (Martin, 2018), which was done by 16% of users worldwide (Searle, 2020). Pointing their phones at egg cartons, consumers find out via the CluckAR app whether the eggs are free-range (van Esch et al., 2019). Other applications help consumers understand nutrition information about packaged food (Juan et al., 2019).

As a new approach to guide decision making at the point of sale, AR technology provides largely untapped potential for marketers (Grewal et al., 2020; Wedel et al., 2020). Instead of changing physical store environments, AR enables retailers to communicate information tailored to the needs of individual customers. By digitally overlaying products or entire shelves, the technology can convey information without physically altering the packaging. Marketers, thus, demand knowledge about how consumers respond to virtual information. Relying on media richness theory (Daft & Lengel, 1986), we expect consumers to welcome the richer and fuller product information. There are first indications for positive AR effects on sales in e-commerce (Tan et al., 2021), but studies in e-commerce and physical stores report initial evidence about negative implications, too (Plotkina & Saurel, 2019; van Esch et al., 2019). Notably, the growing AR research focuses almost exclusively on experiential behaviors in e-commerce (Javornik, 2016b). In these settings, AR mainly helps visualize product usage and product fit, providing a fundamentally different experience and consumer benefit than the AR tools in brick-and-mortar retailing that convey virtual information for physical products. It is, therefore, necessary to shift the focus toward AR’s effectiveness in brick-and-mortar retailing, helping us understand why and when the technology will help and when it may backfire.

This article explores augmented reality-delivered product information (ARPI), which we define as product information whose communication is enabled by AR technology. To our best knowledge, this research is the first to address how the AR-enabled information delivery must be structured to be effective. AR’s inherent properties (esp. interactivity) may unlock unique consumer benefits that are not accessible via traditional communication. To avoid information overload (Hu & Krishen, 2019; Roetzel, 2019) and ensure user friendliness (Rese et al., 2014, 2017), AR apps can let consumers control information delivery. The app shows only those aspects consumers are interested in. Consumers may generally welcome this controllability of information. However, making use of cues-filtered-out theory (Sproull & Kiesler, 1986), we speculate that this controllability may be less effective or even counterproductive under certain conditions.

This research addresses four questions. First, can the virtually enabled information about physical products—via AR—change shopping outcomes in brick-and-mortar stores? The literature confirmed positive effects of both in-store self-service technologies (Evanschitzky et al., 2015) and AR in e-commerce (Tan et al., 2021) but is scarce on the effectiveness of AR tools in brick-and-mortar stores. Second, does permitting consumers control over information delivery foster or hinder the AR’s persuasiveness? While consumers value additional information provided through AR (Spreer & Kallweit, 2014), nothing is known about what happens when consumers filter out seemingly unnecessary information with the help of AR. Third, what mechanisms explain why ARPI’s controllability evokes adverse effects? Past research has considered only the advantages of information delivery via AR (Holdack et al., 2020; Joerß et al., 2021) while neglecting potential disadvantages. We propose that consumers’ perceptions of information comprehensiveness and the interaction quality evoke countervailing effects with regard to how they respond to controllable ARPI. Fourth, what external contingencies limit ARPI use? We explore the detailedness of information as a managerial design element and busy shopping times in the store (hereafter shopping rush hours) as a boundary condition with strong practical implications.

A series of studies (three pre-studies, a main study, four follow-up studies) answers these questions. The main study is an experiment conducted in a field setting involving an AR app at the point of sale specifically designed for this research. The pre-studies are online experiments, and the follow-up studies consist of two online experiments, one experiment conducted in a field setting and another one in the lab. These studies combine different data sources, including usage tracking, observational data, questionnaire data, and scanner data. As a major strength, this article demonstrates—with empirical evidence from different ARPI configurations, products, shopping times, AR devices—the substantive contribution and external relevance of ARPI usage. Establishing ecological validity with field evidence from a hypermarket, this research highlights that the design of the AR is critical for marketers, with real-world implications.

## Conceptual background

### AR technology at the point of sale

#### AR definition

AR systems provide an indirect view of the physical environment via a digital screen. Different devices—including fixed (interactive screens), mobile (smartphones, tablet computers), and mobile systems (head-mounted displays, AR glasses)—enable AR systems. AR overlays and supplements the real world with computer-generated objects, which appear in real time in the same visual space as the physical world elements, such that they appear to coexist (Azuma et al., 2001; Flavián et al., 2019). Users can even interact with the virtual objects (Zhou et al., 2008). Interactivity and augmentation are, therefore, key elements of AR applications (Javornik, 2016a) that we address with this research. In an information context, we investigate interactivity in terms of controllability and augmentation in terms of information detailedness.

#### AR functions in retailing

Several characteristic AR features (Javornik, 2016a) provide value to consumers. First, the AR system can augment the self via virtual try-ons (virtual mirrors) for apparel, cosmetics, glasses, etc. (Hilken et al., 2017; Yim et al., 2017). Second, AR can augment the actual environment. Furniture planners, for example, aid consumers in imagining how furniture would look in their rooms (Holdack et al., 2020; Javornik, 2016a). Third, AR can augment the product or shelf at the point of sale (Joerß et al., 2021; van Esch et al., 2019). For example, consumers scan the product to receive further details via a digital information layer. We focus on this information layer because superimposing information onto shopping realities is a promising AR application for brick-and-mortar retailing.

#### AR in e-commerce vs. brick-and-mortar retailing

While AR improves consumer experiences in online and offline retailing, it is necessary to augment and enrich fundamentally different aspects in both settings. E-commerce already enables unlimited space for product information, but consumers can struggle to imagine the product or product fit. There is no possibility to touch or try the product. Thus, AR in e-commerce characteristically offers three-dimensional visualizations and virtual try-ons (see the overview in Table 7 in Appendix 1 for AR literature in e-commerce). In offline contexts, the AR’s advantage is vice versa. While consumers can easily touch, taste, or try the actual three-dimensional product in the store, the space for additional product information is often restricted. ARPI overcomes this limitation with additional information layers.

#### Extant literature

As shown in Table 7 in Appendix 1, a growing body of AR literature focuses on e-commerce, including functions such as virtual try-ons (Kim & Forsythe, 2008; Yim et al., 2017) or furniture planners (Rauschnabel et al., 2019; Rese et al., 2014). Only a few studies consider traditional retail settings (Table 1). These studies have specific foci on anthropomorphism (van Esch et al., 2019), usage intention toward AR glasses (Holdack et al., 2020), or information evaluation (Spreer & Kallweit, 2014). While these studies provide valuable input, they neither manipulated the AR design nor considered purchase-related variables. Only Joerß et al. (2021) manipulated product ratings in the AR to show that the technology can guide consumer decisions. The lab study, however, did not provide field evidence on purchase behavior, nor did it manipulate the AR controllability or other variables relevant for the present research. Given the lack of insight into offline contexts, we also inform our model by AR research in online contexts, which we adapt due to the different AR functions. Further knowledge comes from research streams on related technologies, such as self-services technologies and personal shopping assistants (e.g., Evanschitzky et al., 2015; Giebelhausen et al., 2014; Mende et al., 2019; Meuter et al., 2000).

**Table 1:** AR at the point of sale literature overview

	Study Type			Manipulation		Moderation			Mediation		DV					
	lab exp.	field setting		information	controllability	detail-edness	medium	rushhour	p. comprehensiveness	other	brand image	PI	choice	purchases		
		study^a)^	exp.													
Spreer and Kallweit (2014)			■	■											books	AR users rated information provided at the POS better than store visitors without access to the AR information. Perceived usefulness and perceived enjoyment increase the intention to reuse.
van Esch et al. (2019)			■							■ ^c)^	■				food	The anthropomorphism of a mobile AR shopping device influences consumers’ experience, which in turn influences their attitude towards the brand.
Holdack et al. (2020)			■							■ ^d)^					furniture	Perceived ease of use, perceived informativeness, and perceived enjoyment influence the attitude towards and usage intention of AR glasses. Perceived enjoyment mediates the influence of perceived informativeness.
Joerß et al. (2021)	■				■								■		food	An AR app with sustainability product information can influence shopping decisions. Effects depend on consumers’ digital device usage, consumption habits, and the technology-as-a-solution-belief.
**This study**		■ ^b)^	■	■	■	■	■	■	■	■ ^e)^	■	■	■ ^f)^	■ ^g)^	food	ARPI can effectively influence brand image, purchase intentions, and purchases. Effectiveness depends on the controllability and detailedness of the product information presented, yet a high level of both creates a backfire effect. The effect is mediated by perceived comprehensiveness and further moderated by the medium and shopping times.

Study type: ^a)^ study in a field setting: participants filled in a questionnaire after using the AR without experimental manipulation. ^b)^ online experiments in pre-study 1 and 2 and follow-up study 1 and 2 as well as lab experiment in follow-up study 4. Manipulation: information (ARPI provided vs. not provided), controllability (controllable vs. uncontrollable). Moderation: detailedness (detailed vs. nondetailed), medium (ARPI vs. booklet), Mediation: p. comprehensiveness = perceived comprehensiveness; other ^c)^ confidence, convenience of transaction, discomfort, innovativeness, product usage barrier, side effect, ^d)^ perceived informativeness, perceived usefulness, perceived enjoyment, attitude, ^e)^ perceived complexity, perceived user friendliness, perceived credibility, presence, perceived novelty, hedonic and utilitarian benefit; DV = dependent variable: PI = purchase intention, choice: ^f)^ preference over competing brand in follow-up studies 1 and 2, purchases: ^g)^ scanner data in follow-up study 3

#### Drivers of AR usage in retailing

Studies in e-commerce (Rese et al., 2014) and offline settings (Spreer & Kallweit, 2014) have identified drivers of the intentions to use or reuse AR applications. These studies, among others, build on the technology acceptance model and its extensions (Venkatesh et al., 2003) to explain the adoption of AR technology (Huang & Liao, 2015; Rese et al., 2014, 2017). Other research refers to flow theory (Novak et al., 2003) to explain the motivational factors (Javornik, 2016b). Many studies consider AR’s utilitarian and hedonic benefits (e.g., Rauschnabel et al., 2019), building on insights into consumers’ adoption and digital technologies’ effectiveness to support shopping (Childers et al., 2001). Scholars usually operationalize hedonic benefit as perceived enjoyment, but consider perceived informativeness as a utilitarian benefit (Dacko, 2017; Holdack et al., 2020). Both aspects improve attitudes toward AR technology in e-commerce (Rese et al., 2014; Yim et al., 2017). Holdack et al. (2020) recently provided evidence that this is also true for brick-and-mortar retailing. Synthesizing the literature shows that hedonic benefits are central to the more playful applications in e-commerce (e.g., virtual try-ons). In brick-and-mortar-retailing, users of AR shopping apps may consider the prospect of receiving additional product information (e.g., about product sustainability) as an efficient means to reduce purchase uncertainty, which they deem a unique utilitarian benefit of AR over traditional shopping experiences (Dacko, 2017; Spreer & Kallweit, 2014). In two preliminary studies motivating the present research, we substantiated that AR product overlays predominantly provide utilitarian benefit to consumers in physical stores (see Web-Appendix A). This paper, therefore, shifts the focus to AR-enabled product information at the point of sale.

### Augmented reality-delivered product information (ARPI)

AR technology can enrich the product on the **A**ugmented **R**eality device’s screen with additional **P**roduct **I**nformation—we coined this ARPI. As a bridge between the digital world and the physical world, AR technology can open a virtually unlimited space for product details in offline stores. AR apps can provide product information precisely at the location where shoppers need it and exactly at the time they decide on the purchase. First evidence supports the assumption that consumers value additional information in offline retailing (van Esch et al., 2019; Holdack et al., 2020; Joerß et al., 2021; Spreer & Kallweit, 2014) but these studies are limited to information evaluations or usage intentions. The present research provides first insights into downstream marketing outcomes (such as brand image and purchase intentions) when systematically varying the content, context, and control of the AR-delivered information (Table 1).

By presenting unlimited information on demand, ARPI has various advantages over the dominating methods for delivering product information through packaging, websites, in-store brochures, etc. AR also differs from matrix codes on the packaging or shelf (e.g., bar codes or quick response (QR) codes; Grewal et al., 2017; Kim & Woo, 2016) for several reasons (Joerß et al., 2021). First, QR codes require additional space on the packaging, while ARPI has fewer space-related restrictions. Second, QR codes increase transaction costs for consumers who are asked to actively search for the code to access additional information. AR technology, by contrast, initiates the information delivery, with the user simply pointing the device at the product or shelf. Third, while the information is displayed separately from the product for shelf QR codes or screens, AR technology overlays the product itself with additional information, which implies stronger information*–*product-fit. For consumers, the information may be mentally linked much closer with the product, as the information appears on the packaging. The third pre-study (Web-Appendix A) supports this assumption. These AR advantages may unlock novel marketing potential for retailers who can digitally add elements to the packaging (e.g., price reduction tags).

### Theory and hypotheses development

Next, we develop a theoretical framework for how ARPI affects information processing at the point of sale. Media richness theory (Daft & Lengel, 1986) posits that an individual’s understanding can be enhanced through a richer medium, particularly in demanding and equivocal situations. A communication medium is considered richer the greater its ability is to reproduce the conveyed information, thereby enabling the receiver’s understanding in a given period. For example, social presence or face-to-face communication usually provides more cues than a technical facilitation of information (Sproull & Kiesler, 1986). Beyond transporting various cues, media richness also stems from the interactivity of the communication medium, immediate interaction, the ability to allow a personal focus, etc. (Qin et al., 2021). Augmentation and interactivity are central aspects of AR (Huang & Liao, 2015; Javornik, 2016a). Compared to traditional media at the point of sale, ARPI can therefore be classified as richer because the technology visually links additional information to the product and allows users to interact with it.

**Fig. 1:**
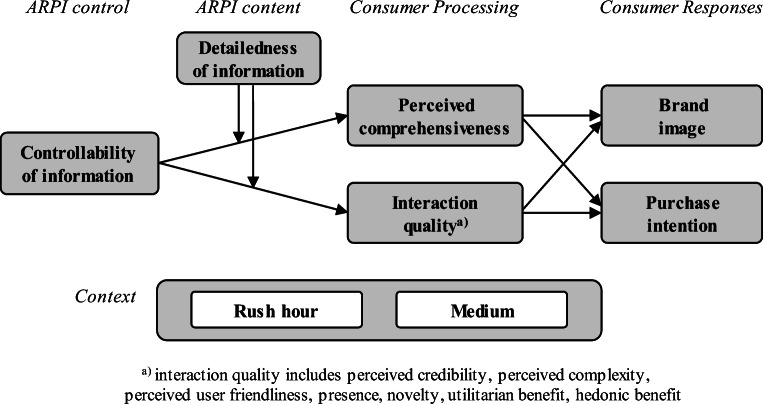
Conceptual model

Using these theoretical grounds as a foundation, the paper develops an ARPI-specific theory that explains why and when ARPI interactivity, specifically information controllability, can evoke an unexpected negative effect depending on certain boundary conditions. This unintended backfire effect^1^ occurs when both controllability and detailedness are high, which is explained by the mediating process via perceived comprehensiveness. The backfire effect particularly arises during busy shopping times. Figure 1 presents the conceptual model that we will now develop.

### Controllability of AR-delivered product information

Interactivity is a well-studied aspect of human-computer interactions in retail contexts (Varadarajan et al., 2010; Yadav & Pavlou, 2014). Scholars have analyzed website interactivity for a long time (Novak et al., 2003; van Noort et al., 2012), and the mechanisms also apply to AR, with interactivity fostering the ease of use and enjoyment of e-commerce enhanced through AR functionality (Pantano et al., 2017). Since interactivity is a broad concept that includes any two-way communication between user and medium, this research elaborates on the controllability of the information, which scholars sometimes even consider as a surrogate for interactivity (Ariely, 2000; Steuer, 1992). In user-machine interactions, controllability refers to the degree to which users can manipulate information delivery (Yadav & Varadarajan, 2005; Yoo et al., 2010). We define controllability as a design aspect that captures to what extent consumers can decide which parts of product information are displayed on the AR device (Fig. 1). While consumers should generally welcome this controllability of the AR device, we ask whether and when controllable information can trigger unintended side effects. We distinguish positive effects on interaction quality and negative effects on perceived comprehensiveness and argue that the controllability backfires under certain conditions, offsetting or even overturning the AR’s beneficial impact.

AR technology and its interactivity is beneficial in several manners for the consumer’s interaction with the device. Although consumers generally appreciate more information, they can be overwhelmed because humans can only process small amounts of information simultaneously (Hu & Krishen, 2019; Roetzel, 2019). Information needs are heterogeneous, and marketers often do not know exactly which pieces of information meet consumers’ current needs (Ariely, 2000). Information controllability could therefore simplify decision making because consumers can tailor the delivered information to their interests (concerning ingredients, production processes, origins, etc.) (Häubl & Trifts, 2000). Building on this rationale, one would initially expect that the controllability of information should improve perceptions of the interaction quality with ARPI.

#### Backfire effect of AR controllability

In retail settings, the AR-enabled controllability of information delivery may have a disadvantage, ignored thus far, that reduces or even cancels out the mentioned benefits. Given that ARPI is primarily helpful for utilitarian consumption decisions (see pre-studies 1 and 2 in Web-Appendix A), its effect on more downstream variables (brand image, purchase intention) will not only be driven by interaction quality. The question of whether consumers feel comfortable with the information collected will be even more important. We suggest that the controllability of information can provide a shortcut to the most relevant pieces of information at the expense of richer, fuller, and broader information.

While ARPI controllability enables consumers to select the information they value, this also implies filtering out certain pieces of information. Building on the cues-filtered-out perspective (Yadav & Varadarajan, 2005), a high degree of controllability implies that users who selectively choose content miss out on the non-chosen content (i.e., cues are filtered out). If consumers can control the AR-delivered information content, they may consider the seen information as less rich and may even fear that cues have been lost. While this unconsidered information might not be of focal interest, it may still serve as a signal to the amount and type of information that is principally available, shaping the user’s impression about the comprehensiveness of information. In other areas, such as social media, it has been shown that the fear of missing out on information creates stress and affects subsequent behaviors negatively (Tandon et al., 2021, 2022). We assume that these negative aspects also apply to ARPI communication.

Information controllability can create the impression of not being fully aware of all available information. By contrast, if users cannot control the AR-delivered content, they have to browse for the information they deem most relevant. During this search, they notice—consciously or unconsciously—the extent of other product information that they may not deliberately process but which subtly sends signals of being well-informed to them. This peripheral perception with regard to the comprehensiveness of information is lost when the AR device allows users to control the displayed information. Since consumers may not be exposed to all potentially available information chunks, they are less likely to develop a vivid imagination of the extent of information at hand. As a result of this, we propose that the controllability of ARPI decreases consumers’ perceptions of information comprehensiveness.

Since this is relevant to retailers, we discuss next *when* controllability’s detrimental effects on perceived comprehensiveness spill over to downstream marketing-relevant variables. Rese et al. (2014, 2017) show that in e-commerce, the perceived informativeness of an AR guides the perceived usefulness and, in turn, consumers’ usage intentions. We transfer this to the brick-and-mortar context. Plausibly, the magnitude of the perceived information comprehensiveness is contingent on the amount of information that is readily available and disclosed by the brand.

### Detailedness of information as a boundary condition of the ARPI’s backfire effect

Information detailedness is the extent of elaboration about a specific product attribute. Detailedness is a core property of augmentation and, thus, an inherent AR aspect. Expecting consumers to decide rationally, companies and retailers may be inclined to provide as much information as possible. Arguably, a greater amount of information delivered at the point of sale allows for more informed decisions. Spreer and Kallweit (2014) accordingly demonstrate that bookstore visitors who accessed additional information via AR evaluated offers better than those who did not. Consumers are, however, often overwhelmed when shopping environments offer too much information (Hu & Krishen, 2019; Roetzel, 2019). In a similar vein, too much variety and choice at the point of sale can harm (Chernev, 2003; Gourville & Soman, 2005).

We reasoned earlier that based on the cues-filtered-out notion (Yadav & Varadarajan, 2005), high degrees of controllability imply that users who selectively choose content also miss out on the non-chosen content (i.e., cues are filtered out). This negative effect of ARPI controllability should be more likely to occur when the available information has more detail. This might be so because the greater the amount of information chunks is that are potentially accessible, the greater is the likelihood for users to develop the impression of missing out on relevant cues. According to media richness theory, media providing fewer cues will inhibit decision quality (Dennis & Kinney, 1998; Maity et al., 2018). We, therefore, make the counterintuitive, AR-specific claim that a controllable (vs. uncontrollable) AR-enabled information delivery is less persuasive and less effective for marketing outcomes, the more detailed the delivered information is. We expect that the detailedness of the information moderates how ARPI controllability affects marketing outcomes. ARPI controllability weakens the marketing outcomes when information detailedness is high. This detrimental controllability effect is weaker when information detailedness is low because AR users will miss out on fewer details.

In terms of marketing outcomes relevant to retailers, we consider relational and transactional variables because recent research has stressed the need to consider both types of downstream variables (Güntürkün et al., 2020). As a rather relational variable, we consider brand image. We expect that perceptions of being comprehensively informed will spill over to brand image. The sensation of being aware of all relevant information about a brand will improve the opinions about the brand, such as liking, quality evaluations, and trust (Hoffmann & Müller, 2009).

#### H1a

There is an interaction effect between ARPI controllability and information detailedness on brand image. That is, ARPI controllability weakens consumers’ brand image perception when information detailedness is high; this effect is attenuated when information detailedness is low.

Furthermore, we consider the transactional outcome variable, i.e., purchase intention, which is typically more strongly guided by competence than by the warmth component of the provider (Güntürkün et al., 2020). Useful product information increases clarity and reduces doubts and risks. This enables consumers to make product choices with greater decision comfort, ultimately increasing purchase likelihood (Heller et al., 2019; Hilken et al., 2017).

#### H1b

There is an interaction effect between ARPI controllability and information detailedness on purchase intention. That is, ARPI controllability weakens consumers’ purchase intention when information detailedness is high; this effect is attenuated when information detailedness is low.

#### Mediating effect of perceived comprehensiveness

Our theory suggests that the backfire effect is due to a loss in perceived comprehensiveness of the information that is accessible through the AR device. We define perceived comprehensiveness as consumers’ sense of being aware of all the accessible details and of being fully informed. Following our reasoning about how information controllability affects the perception of being aware of all the available information, we suggest that reduced perceptions of such information comprehensiveness underlie the patterns proposed with H1. We, therefore, predict the following mediation: Information controllability affects perceived comprehensiveness, which, in turn, affects brand image. Notably, the mediation of controllability via perceived comprehensiveness is conditional on the degree of information detailedness. When information detailedness is high, the information controllability will lead to lower levels of perceived comprehensiveness; as a result, consumers’ perception of not being fully informed will negatively spill over to the brand image.

#### H2a

The effect of ARPI controllability on brand image is mediated by information comprehensiveness when information detailedness is high. This mediation effect is attenuated when information detailedness is low.

Besides the imprint on the rather relational outcome variable brand image, we also expect an effect on the transactional outcome variable purchase intention. The more the consumers have a perception of being fully informed, the better they are able to justify purchasing the product.

#### H2b

The effect of ARPI controllability on purchase intention is mediated by information comprehensiveness when information detailedness is high. This mediation effect is attenuated when information detailedness is low.

While controllability may hamper the ARPI’s effect under the condition of high information detailedness, there are also several alternative mediators of AR controllability that imply positive effects. We need to explore these positive effects to fully understand the backfire effect. The variables tested in AR research are prime candidates for alternative mediators (Table 7 in Appendix 1). These variables include perceived credibility, perceived complexity, user friendliness, presence, novelty, utilitarian benefit, and hedonic benefit. However, past research has focused on e-commerce and often used the adoption intention as dependent variables. These mediators, therefore, have to be tested in the context of ARPI in a brick-and-mortar setting and for more downstream marketing outcomes. The rationale for the potential mediating effects is given in Table 8 in Appendix 2. Still, all these positive effects compete with (and are potentially dominated by) the inhibiting effect of reduced perceived information comprehensiveness.

#### H3a/b

The effects of ARPI controllability on (a) brand image and (b) purchase intention are mediated by alternative mediators when information detailedness is high. The mediation effect is attenuated when information detailedness is low.

### Further boundary conditions of the ARPI’s backfire effect

#### Rush hours

Besides the moderating role of the AR detailedness, the proposed backfire effect of ARPI controllability may also depend on contextual factors involving strong practical relevance, such as rush hours (Irmak et al., 2020). In contrast to the e-commerce settings (e.g., Rese et al., 2014; Yim et al., 2017; Tan et al., 2021; Appendix A), the presence of other people in physical stores strongly influences the shopping experience and particularly consumers’ perceived stress level (Lucia-Palacios et al., 2018; Santini et al., 2020). Building on Lewin’s (1939) field theory, research has shown that particularly task-oriented shoppers feel more stress and display less patronage behavior if retail crowding is high (Baker & Wakefield, 2012). Against this background, we assume that the ARPI backfire effect may be more prominent during busy shopping times. Market reports, such as *Bring! Shopper Report Germany* (Bring!, 2020, p. 5), imply that retail crowding varies systematically across weekdays and day times. In busy shopping times (which we label “rush hours”), many consumers crowd around the same shelves. This detracts from a calm and relaxed atmosphere that may be required to interact intensively with a digital device for accessing additional product information. Shoppers are often urged to decide quickly and move on to let other consumers reach the shelf. This will determine whether shoppers construe the shopping situation as work or as a pleasant, explorative experience (Babin et al., 1994).

We, therefore, expect that retail crowding and time pressure govern whether customers value having control over the AR-enabled information presentation. Although controlling the information may sometimes help save time, it may increase the probability that consumers miss out on relevant information in stressful situations. Therefore, we investigate whether consumers respond less favorably to detailed information presented in a controllable manner during rush hours. Building on the rationale that retail crowding evokes stress that impairs attention and working memory, Gelbrich and Sattler (2014) demonstrate that technology anxiety hampers the usage of self-service technologies more strongly when retail crowding is high. Transferred to our context, we assume that controllable and detailed AR-delivered product information may unfold its unfavorable effect particularly when many consumers shop during rush hours (e.g., after work).

#### H4

The backfire effect of ARPI more likely occurs in busy rush hours but is less likely in more relaxed shopping times.

#### Medium

The task-media fit hypothesis (Mennecke et al., 2000), an extension of the media richness theory, argues that the medium needs to be adjusted to the task for better interaction effectiveness. The controllability effect and its interplay with other aspects (detailedness) should also depend on the medium through which the information is transported. As such, the proposed backfire effect should primarily be an issue for digital devices. In principle, ARPI increases media richness when combining the product’s image with virtual elements, thereby providing more cues (Dennis & Kinney, 1998). Yet, the controllability filters out cues, which reduces media richness (Yadav & Varadarajan, 2005). Controllability enables consumers to jump directly to the exact piece of information they are interested in while omitting supposedly irrelevant information. Since this happens digitally, users do not gain experience with the selected route that led to this detail, nor are they exposed to the structure by which the information is organized or the extent of other potentially available details. The backfire effect of controllability should be particularly prominent here. In nondigital environments, such as print media (e.g., books, booklets, newspapers, flyers), tools like content overviews, page numbers, tabs, and registers enable a certain sort of adaptability too. These tools allow easier retrieval of the piece of information the user is interested in. Although booklet users do not read all other information when skipping to the relevant detail, they still gain an impression of the wealth of available information. For example, when selecting a tab or opening the target page, users are still exposed to the other pages and can feel the booklet’s thickness, giving them a sense of the available information. Consequently, we assume no or a much weaker backfire effect in nondigital media.

#### H5

The backfire effect of ARPI more likely occurs when information is delivered in augmented realities but is less likely when information is delivered in physical realities.

## Overview of studies

We ran three pre-studies, a main study in a field setting, and four follow-up studies (Table 9 in Appendix 3). The pre-studies, which are reported in Web-Appendix A, set the stage for our main research: First, pre-study 1 and pre-study 2 demonstrate that ARPI outperforms a non-AR condition with regard to brand image, purchase intention, as well as perceived utilitarian benefit and perceived hedonic benefit. Second, confirming our overall premise, pre-study 1 and pre-study 2 further show that mainly utilitarian benefits foster brand image and purchase intention in the ARPI condition. Third, pre-study 3 additionally shows that ARPI is superior to QR codes in creating the information-product-fit. Since the additional information appears virtually on the packaging, ARPI users mentally linked the information closer with the product than users of QR codes.

The main study is an experiment conducted in the field setting of a hypermarket, involving an ARPI app crafted especially for this research. A multi-factorial design manipulates the AR-delivered information’s controllability and detailedness. Besides the mediating effect via perceived comprehensiveness, we test various indicators of interaction quality, including perceived complexity, user friendliness, and perceived credibility. The roles of information detailedness, medium, and rush hours as boundary conditions are explored. We ran four follow-up studies involving different ARPI configurations, product categories, and experimental designs to provide more evidence for selected hypotheses and to establish the external validity of our findings.

## Main study

### Design

We ran a 2 (controllable vs. uncontrollable) × 2 (detailed vs. nondetailed) between-subjects experiment at the point of sale. Conducting the experiment in a field setting (i.e., where customers naturally are and undertake their shopping) enables us to achieve ecological validity and test the ARPI effects in the natural environment (Gneezy, 2017; Grewal et al., 2018).

#### Development of the ARPI and its content

The ARPI and its content were specifically developed for this study. This allowed us to standardize the information delivered by the AR device. We chose the cereal domain because the relevant information can be systematically varied for this product type. We focus on information about sustainability, as this is a product attribute that appears to be highly relevant, marked by a high level of uncertainty and ambiguity,^2^ and which has been tested in a retailing context with AR (Joerß et al., 2021; van Esch et al., 2019). According to media richness theory, richer media can effectively help overcome the uncertainty in such contexts (Daft & Lengel, 1986). This main study included a specific brand to rule out distortions from brand preferences and reduce nuisance variance.

#### ARPI

We developed the application with the Vuforia Framework in the unity engine. The participants received the app on a tablet computer (Android, 10.1 in., 1920 × 1200 pixels, camera with 13 megapixels) at the point of sale in a hypermarket. When pointing the tablet camera at the shelf, the real world (e.g., the product and shelf) was displayed, giving the impression of looking through a glass pane. If participants focused on a specific product, an overlay with product information appeared, hovering half a centimeter in front of the product package. We pretested and optimized the app’s usability with consumers, company managers, and business researchers. Users could interact with the overlay, browse through the product information, and open further information. Figures 6, 7, Table 10 in Appendix 4 shows the ARPI in use.

#### Structure of information

The target product was a major cereal brand, which we selected because cereal products vary substantially in sustainability depending on the composition and production methods. Three flavors (chocolate, fruit, and honey nut) account for individual taste preferences. We developed the sustainability information’s content and structure from several pre-tests and discussions with the company’s product and marketing managers. For each product, the managers developed three statements about ecological, social, and regional aspects and provided three arguments for each statement. We reworked the statements and details in intensive discussions with the company to ensure that they were comparable in text length, comprehensibility, and tonality. This process resulted in systematic and standardized sustainability content for each of the three flavors (Figs. 6, 7, Table 10 in Appendix 4). In a two-factorial design, we independently manipulated the controllability and detailedness of the AR-enabled information.

#### Controllability

In the controllable condition, the users received the initial information regarding whether the products are ecologically, socially, and regionally produced. When interested in obtaining further information, they could click on a particular category to see three corresponding statements and, eventually, further details. Participants in the uncontrollable condition received the full information and did not have the opportunity to tailor the presentation (extend or hide details); they had to scroll to find and access the information they were interested in.

#### Detailedness

We split the delivered information into general statements and supporting details (Figs. 6, 7, Table 10 in Appendix 4). The complete information in the app consisted of 1337 words (10,517 characters). The statements made up about one-fourth of the text (in sum 378 words, 2858 characters). Participants in the nondetailed condition saw these statements, while the detailed condition also included three details per statement (in sum 959 words, 7659 characters). The statements and details, on average, consisted of 12.4 words (SD = 2.1) and 97.4 characters (SD = 10.8). Participants in the detailed conditions had access to all statements and details, whereas those in nondetailed cells had access to statements, but not to the arguments at the lower level. In the uncontrollable-detailed cell, they saw a long text and needed to scroll to search the information. If they were in the controllable-detailed cell, they could click on the statements they were interested in to see the three corresponding details.

#### Medium as an additional control group

To differentiate ARPI from the more traditional means of informing consumers at the point of sale, we designed four versions of a paper booklet, mirroring the four app manipulations. In the uncontrollable-detailed version, participants received a booklet with statements and the corresponding details. In the uncontrollable-nondetailed version, we provided only the statements, not the details. In the controllable versions, the same content was provided, but we added a content overview and several tabs on the right-hand side of the booklet to provide quick access to the relevant information.

#### Procedure

We ran the experiment in a hypermarket offering more than 80,000 articles, and located in a shopping mall of a medium-sized city. The experiment took place during four weeks. A planned schedule helped systematically vary the data collection across different times (9:00 a.m. to 12:30 p.m., 12:30 p.m. to 4:00 p.m., and 4:30 p.m. to 7:30 p.m.) and week days (Monday to Saturday). The hypermarket was closed on Sundays. Each slot used all four treatments (controllability × detailedness) and participants were randomly assigned.

Trained interviewers approached shoppers entering the hypermarket. Every time the interviewers were not busy explaining the app function to one of the participants, they asked the next shopper entering the hypermarket whether he or she was willing to participate in the study. There was no exclusion criterion (e.g., in terms of age, gender, etc.). Approximately 10 % of the approached shoppers agreed to participate. The interviewers briefly introduced the participants to the app’s basic functionality for approximately 30 s. The interviewers asked the participants to use the app for as long as they wanted to learn more about the cereals. They did not urge the participants to buy the cereals. The participants were instructed to walk unaccompanied to the cereals shelf where we provided a tablet with the ARPI (about five meters from the entrance on the main floor, see the map of the hypermarket in Web-Appendix B). The ARPI’s instructions and usage were, therefore, separated in time and space. The shoppers registered in the AR app with an anonymous code, ensuring that participants used the app only once. They logged in with their code and then used the ARPI to inform themselves about the target products in the absence of an interviewer. This ensured that they could do their shopping and use the ARPI at their own pace. After completing their shopping, the interviewer asked them at the hypermarket’s exit to complete a questionnaire that was matched with the ARPI app data (e.g., time taken to consider the information) and the interviewers’ observations (e.g., how full their shopping cart was). The shoppers received a cereal bar for participating in the study.

#### Measurements

We applied random assignment to the experimental condition, as well as multiple data sources to reduce common method variance (Table 2). We included several measures to reduce or control for the noise that comes with experiments in field settings.

**Table 2:** Overview of the multiple sources design (main study)

Type of variable	Variable	Source
Treatment	Controllability of the AR app	Manipulated via ARPI
	Detailedness of information	Manipulated via ARPI
Dependent variable	Purchases	Self-reported^a^
	Purchase intention	Self-reported
	Brand image	Self-reported
Boundary conditions	Rush hour (consumer stress)	Observed
	Medium	Manipulated
Mediator	Perceived comprehensiveness	Self-reported
Alternative mediators	Perceived credibility	Self-reported
	Perceived complexity	Self-reported
	Perceived user friendliness	Self-reported
Controls	Sociodemographics (gender, age)	Self-reported
	Device experience (tablet ownership)	Self-reported
	Attitude (sustainability attitude)	Self-reported
	Product category knowledge (food knowledge)	Self-reported
	Usage time^c^	Tracked via ARPI
	Shopping cart filling level	Observed^b^

^a^Validated via samples of shopping cart observations. ^b^ Only applied as robustness check with a reduced sample of 340

^c^Browsing time in the uncontrollable—nondetailed condition consists of the time that users spent browsing and reading the statements, starting from the moment the statements appeared until they disappeared, either by scrolling, scanning another product, or quitting the app. In the uncontrollable—detailed condition, we measured the time for reading the statements plus the arguments. In the controllable—nondetailed condition, we measured the time spent at the statement level, clicking through the various statements. In the controllable—detailed condition, we added the time spent browsing the arguments

We designed the app to track the usage time. On average, the participants used the ARPI for 59.07 s (max = 277.52 s).^3^ Table 11 in Appendix 5 presents the wording and the sources of all scales. We measured purchase intention with one item (“I intend to buy cereals of XY in the future,” M = 2.80) adapted from Dodds et al. (1991) and brand image with three items adapted from Hoffmann and Mueller (2009) and Mai et al. (2014) on five-point scales (M = 3.66, Cronbach’s α = .89). To operationalize the mediating mechanisms of the information’s comprehensiveness (M = 3.73, α = .91), we created a four-item scale on the basis of Rese et al. (2014, 2017) and Mai et al. (2014). We included several variables to control for and rule out alternative mediating processes (for two-item scales, we show Spearman and Brown’s ρ, Eisinga et al., 2013). The participants indicated perceived credibility on a two-item rating scale (M = 3.82, ρ = .90), perceived complexity on a two-item scale (M = 2.14, ρ = .76) adapted from Geissler et al. (2001), and perceived user friendliness with three items (M = 3.78, α = .74) adapted from Srinivasan et al. (2002). As controls, we measured the self-reported product category knowledge in the food domain with two five-point items (M = 3.81, ρ = .80) adapted from Chang (2004) and sustainability attitude with two nine-point items (M = 6.84, ρ = .84) based on Whitmarsh and O'Neill (2010). Confirmatory factor analysis (AMOS 25.0) with all multi-item constructs shows a good model fit (χ^2^_(115)_ = 198.61, χ^2^/d.f. = 1.73; CFI = .98; RMSEA = .04). The analysis confirms discriminant validity, because each construct’s average variance extracted exceeds the maximum of the squared correlations with all latent variables (Fornell & Larcker, 1981). To track purchases, consumers indicated in the questionnaire whether they had bought a box (or more) of the focal brand.^4^ Trained interviewers estimated and documented further details on five-point scales. First, they coded the participants’ apparent stress. We dichotomized this variable to relaxed (59.2%) and stressed (40.8%). Second, they tracked how full the shopping carts were, because consumers are potentially more likely to purchase cereals if they purchase more items overall.

#### Rush hour as a boundary condition

To gain more managerial insights, we created a rush hour index. Following previous field studies (e.g., Irmak et al., 2020), we use daytime to capture rush hour. We distinguish no rush hour (Monday–Friday morning and Saturday afternoon) vs. rush hours (Monday–Friday afternoon and Saturday morning).^5^ This distinction is in line with the *Bring! Shopper Report* (Bring!, 2020, p. 5) that presents the daily distributions of shopping trips for Germany, which are left-skewed for weekdays (Monday to Friday), with a peak at about 5 p.m. On Saturdays, the distribution is right-skewed, with a peak at 11 a.m. We conclude that the rush hour is characteristically in the afternoon on weekdays and in the morning on Saturdays. We validate the rush hour shopping based on the interviewers’ observation of the shoppers’ stress level (0 = relaxed, 1 = stressed). We found that on weekdays (Monday to Friday), the stress level was rather low before 2:00 p.m. and higher in the afternoon (M_morning_ = .29 vs. M_afternoon_ = .48), while on Saturdays the pattern is flipped (M_morning_ = .39 vs. M_afternoon_ = .13).

#### Sample

We gathered data of 271 shoppers who used the ARPI. For contrasting the ARPI to traditional means of informing consumers, we asked 153 additional shoppers to use a booklet providing the same information. Within both groups, we randomly assigned participants to one of the four treatments.^6^ The exclusion of shoppers who used the ARPI app for less than three seconds resulted in 250 ARPI users.^7^ The excluded participants do not differ from the sample in terms of gender (χ^2^(1) = 1.28, *p* = .258) or age (t(415) = .24, *p* = .814). The age of the participants ranged from 16 to 82 years, with a mean of 39.6 years (SD = 17.8); 39.9% of the participants were men.

## Results

### Backfire effect

We ran an ordinary least squares (OLS) regression testing the influence of the experimental controllability condition on the perceived comprehensiveness as dependent variable, which shows a statistically significant influence (β = −.231, t = −2.557, *p* = .012). The effect remains stable (β = −.206, t = −2.216, *p* = .029) when entering the control variables gender (β = −.118, t = −1.230, *p* = .221), age (β = −.101, t = −1.057, *p* = .293), device ownership (β = .023, t = .246, *p* = .806), product category knowledge (β = −.196, t = −2.007, *p* = .047), and sustainability attitude (β = .248, t = 2.311, *p* = .023).

**Fig. 2:**
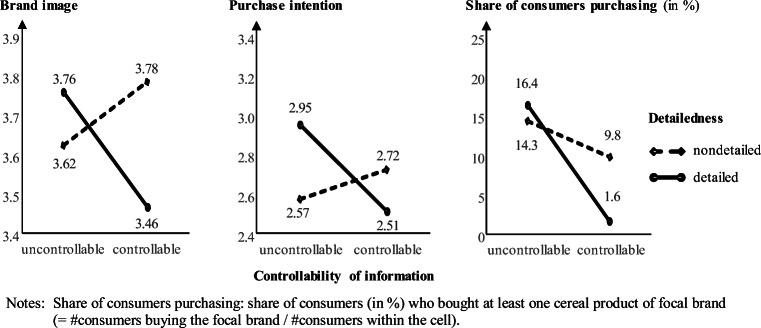
Backfiring-effect of information controllability (main study). Share of consumers purchasing: share of consumers (in %) who bought at least one cereal product of focal brand (= #consumers buying the focal brand / #consumers within the cell)

Next, we examined the backfire effect on marketing-relevant downstream variables and explored the moderating role of information detailedness for brand image (nondetailed: M_uncontr._ = 3.62, M_contr._ = 3.78, detailed: M_uncontr._ = 3.76, M_contr._ = 3.46), purchase intentions (nondetailed: M_uncontr._ = 2.57, M_contr._ = 2.72, detailed: M_uncontr._ = 2.95, M_contr._ = 2.51), and purchases (share of consumers’ purchases per cell: nondetailed: share_uncontr._ = 14.3%, share_contr._ = 9.8%, detailed: share_uncontr._ = 16.3%, share_contr._ = 1.6%). Regression analysis (Table 3) confirms AR-controllability’s suggested backfire effects on brand image and purchase intention under the condition of information detailedness (plots in Fig. 2), which supports H1. Furthermore, a chi-square test shows that the four experimental conditions differ in terms of purchases (χ^2^(3) = 8.32, *p* = .040, contingency coefficient *C* = .18; Fig. 2, right panel, for descriptive statistics). A difference arises between the controllable and uncontrollable versions (χ^2^(1) = 6.24, *p* = .012, *C* = .16), but not for the detailed and nondetailed versions (χ^2^(1) = .79, *p* = .375). A logistic regression (Table 3, right panel) with purchase as the dependent variable (including the controls) confirms that ARPI controllability’s effect on the purchases of the focal product decreases with greater information detailedness (b = −2.63, Wald(1) = 4.41, *p* = .036).^8^

**Table 3:** Backfire effect of AR-controllability and detailedness (main study)

Purchase of the product promoted in the ARPI	Brand Image ^a)^			Purchase Intention ^a)^			Purchases ^b)^				
	β	t	p	β	t	p	b	SE	Wald	p	odds exp.(b)
Constant							−6.878	1.886	13.302	<.001	.001
*Controls*											
Gender^1^	−.007	−.094	.925	.038	.534	.594	−.677	.531	1.626	.202	.508
Age	−.048	−.700	.485	−.097	−1.393	.165	.019	.016	1.420	.233	1.019
Device experience^2^	.063	.936	.350	.031	.457	.648	.152	.472	.104	.747	1.165
Product category knowledge	.126	1.718	.087	.021	.290	.772	−.009	.247	.001	.970	.991
Sustainability attitude	.049	.641	.522	.075	.981	.328	.332	.208	2.546	.111	1.394
Treatment											
Controllability	.157	1.433	.153	.123	1.277	.203	**2.495**	**1.082**	**5.322**	**.021**	**12.124**
Detailedness	.124	1.382	.168	**.191**	**2.121**	**.035**	2.123	1.132	3.514	.061	8.356
Controllability × detailedness	**−.268**	**−2.299**	**.022**	**−.272**	**−2.324**	**.021**	**−2.625**	**1.250**	**4.410**	**.036**	**.072**
−2 log-likelihood							139.256				
Cox & Snell R^2^							.079				
Nagelkerke’s R^2 a)^ / R^2 b)^	.050			.046			.159				

^a)^OLS regression, ^b)^ Logistic regression models. β = standardized coefficients. ^1^0: male, 1: female, ^2^0: no tablet ownership, 1: tablet ownership

### Mediators of the backfire effect

We ran mediation analyses to tap the backfire effect’s sources (Table 4). We test whether the backfire effect occurs because AR controllability activates perceptions of a loss in comprehensiveness, especially with high levels of detailedness. As expected for detailed ARPI, we observe full mediation for the detrimental controllability effect on both brand image (indirect effect a*b = −.17, 95% confidence interval CI_95%_: −.37; −.02) and purchase intention (a*b = −.10, CI_90%_: −.23; −.01), as path c’ becomes non-significant when including perceived comprehensiveness as the mediator. This supports our assumption, specified with H2, that a controllability-induced lack of perceived comprehensiveness is responsible for the backfire effect. This negative process does not occur for lower levels of information detailedness.

**Table 4:** Mediation analyses (main study)

Detailedness	Mediator	Response	a (IV ➔ MV)			b (MV ➔ DV)			c (IV ➔ DV)			c’ (IV ➔ DV)			a*b		
			b	t	p	b	t	p	b	t	p	b	t	p	b	LLCI	ULCI
Detailed	Perceived comprehensiveness	Image	−.402	−2.216	.029	.415	4.741	<.001	−.362	−2.009	.047	−.195	−1.160	.249	−.167	−.372	−.017 *
		Purchase Intention				.257	2.617	.010	−.442	−2.334	.021	−.339	−1.796	.075	−.103	−.265	.005 †
	Perceived credibility	Image	−.218	−1.237	.219	.406	4.464	<.001	−.362	−2.009	.047	−.273	−1.634	.105	−.089	−.246	.054
		Purchase Intention				.295	2.948	.004	−.442	−2.334	.021	−.377	−2.049	.043	−.063	−.182	.040
	Perceived complexity	Image	−.516	−2.736	.007	−.022	−.236	.814	−.362	−2.009	.047	−.373	−1.994	.049	.011	−.097	.149
		Purchase Intention				−.064	−.658	.512	−.442	−2.334	.021	−.475	−2.419	.017	.033	−.077	.177
	Perceived user friendliness	Image	.170	.405	.405	.317	4.182	<.001	−.362	−2.009	.047	−.397	−2.489	.014	.057	−.073	.199
		Purchase Intention				.352	4.310	<.001	−.442	−2.334	.021	−.492	−2.858	.005	.061	−.076	.216
Nondetailed	Perceived comprehensiveness	Image	−.298	−1.722	.088	.197	1.760	.081	.295	1.467	.145	.353	1.752	.083	−.059	−.189	.025
		Purchase Intention				.248	2.273	.025	.299	1.506	.135	.373	1.891	.061	−.074	−.183	.017
	Perceived credibility	Image	.378	2.083	.040	.587	6.379	<.001	.295	1.467	.145	.073	.416	.678	.222	.032	.436 *
		Purchase Intention				.272	2.625	.010	.299	1.506	.135	.196	.993	.323	.103	.005	.246 *
	Perceived complexity	Image	−.062	−.362	.718	−.232	2.190	.031	.295	1.467	.145	.279	1.415	.160	.015	−.070	.140
		Purchase Intention				−.043	−.380	.705	.299	1.506	.135	.296	1.485	.141	.003	−.044	.056
	Perceived user friendliness	Image	.512	3.100	.002	.232	2.089	.039	..295	1.467	.145	.161	.821	.413	.125	−.010	.334 †
		Purchase Intention				.119	1.039	.301	.299	1.506	.135	.231	1.141	.257	.062	−.063	.214

Mediation analyses with PROCESS (model 4, each mediator tested separately), b = unstandardized coefficients; all variables centralized, IV = controllability. Included control variables: gender, age, device experience, product category knowledge, sustainability attitude. a: regression of the mediator on controllability in a single linear regression analysis; b: regression of DV on the mediator in a multiple regression analysis; c: regression of DV on controllability in a single linear regression analysis; c’: regression of DV on controllability in a multiple regression analysis; a*b: indirect effect; *Bootstrapping (CI-95%, 5000 samples): LLCI: lower limit, ULCI: upper limit. †Bootstrapping with CI-90% applied to test for the p < .10-level

### Alternative mediators

The analysis (Table 4) shows that further processes become activated, but only in the nondetailed condition. AR controllability elicits a positive impact on the dependent variables operating through improved credibility perceptions (brand image: a*b = .22, CI_95%_: .03; .44; purchase intention: a*b = .10, CI_95%_: .01; .25) when information detailedness is low. AR controllability reduces perceived complexity, but does not affect the dependent variables. Ruling out these alternative mediators confirms the AR-induced perceptions of comprehensiveness as the relevant mechanisms. The backfire effect of AR controllability, therefore, must be traced back to a growing extent of information detailedness making AR controllability increasingly more likely to provoke a perceived loss in comprehensiveness (negative mechanism).

### Rush hour as a boundary condition of the backfire effect

**Fig. 3:**
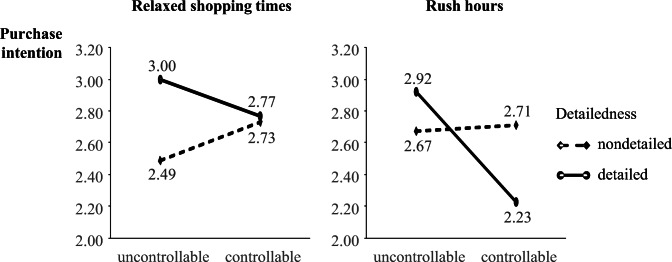
Moderating effect of busy shopping times on purchase intention (main study)

A moderation analysis tests the effect of controllability on purchase intention conditional on detailedness at specific shopping times (relaxed shopping hours vs. rush hours) (Fig. 3). A spotlight analysis illustrates that AR controllability’s negative effect triggered by detailed information is particularly prominent at specific times (Table 5). Predominantly at times when shoppers are generally stressed (Monday–Friday afternoons and Saturday mornings), the controllability of detailed information exerts a negative effect on purchase intention (b = −.64, SE = .31, t = 2.05, *p* = .042), which supports H4.

**Table 5:** Effects of controllability on intentions depending on rush hour and detailedness (main study)

Stress during shopping time		Detailedness	b	SE	t	p	LLCI	ULCI
Relaxed shopping	(Mo-Fr < 2 pm, Sat > 2 pm)	Nondetailed	.416	.353	1.180	.239	−.279	1.112
Relaxed shopping	(Mo-Fr < 2 pm, Sat > 2 pm)	Detailed	−.333	.333	−1.003	.317	−.989	.322
Rush hour	(Mo-Fr > 2 pm, Sat < 2 pm)	Nondetailed	.193	.322	.598	.550	−.442	.828
Rush hour	(Mo-Fr > 2 pm, Sat < 2 pm)	Detailed	−.637	.311	−2.048	.042	−1.251	−.024

OLS regression with PROCESS (model 3), b = unstandardized coefficients. Included control variables: gender, age, device experience, product category knowledge, sustainability attitude. Bootstrapping (95% CI, 5000 samples): LLCI: lower limit, ULCI: upper limit

### Comparing ARPI and booklets

**Fig. 4:**
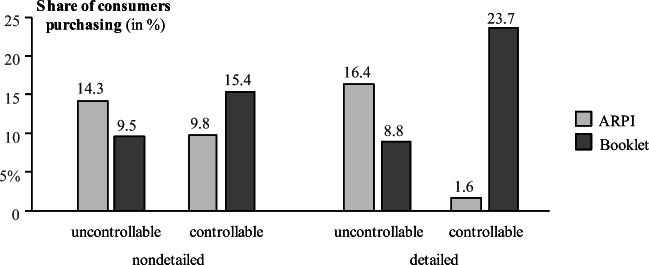
Moderating effect of medium on purchases (main study)

To test if the backfire effect is truly AR specific, we compare ARPI to traditional media. Logistic regression confirms that the backfire effect depends on the medium (three-way interaction medium × controllability × detailedness: b = 3.55, Wald(1) = 4.54, *p* = .033). For the booklet, there is no interaction effect between AR controllability and information detailedness on purchases, and neither on brand image and purchase intentions (Web-Appendix B for details). Consistent with our assumption, there is no backfire effect in the booklet condition. As visualized in Fig. 4, the ARPI outperforms the booklet in both uncontrollable conditions (either nondetailed or detailed). However, in the controllable, detailed condition in particular, the booklet is strikingly more effective than the ARPI. The findings support H5.

## Discussion

Although ARPI controllability reduces perceived complexity, controllability also negatively affects consumers’ perceived comprehensiveness of the AR-enabled information. The study confirms that although a hypermarket’s shoppers welcome both detailedness and controllability of information at the point of sale, their combination creates a backfire effect. When retailers provide detailed AR-enabled information but let consumers control which information is displayed, the consumers ironically fear that they are less comprehensively informed, leading to reduced purchase intentions, negative brand image, and ultimately fewer purchases. This effect predominantly arises during busy shopping times. A comparison with a control group using a printed booklet further confirms that this backfire effect is truly AR-specific.

## Follow-up studies

### Follow-up study 1: Backfire effect more prominent for higher degrees of controllability

#### Objective

We now seek to understand better when controllability impairs perceived comprehensiveness, and ultimately brand image and purchase intentions (referring to H2). We spotlight different degrees of ARPI controllability. In the main study, users could access multiple paths of information (category-statements-details) and they could decide when to exit the ARPI. Consumers may react differently if they can choose only one path of information (category-statements-details) or can follow multiple paths with an exit option. For this reason, we manipulated the degree of ARPI controllability. We focus on detailed information because the backfire effects were particularly strong in this condition in the main study. For generalizability, we use another brand and chocolate cereal bars as the product category.

#### Design

We ran a between-subjects experiment with three treatment groups and a control group. Similar to recent AR research (Barhorst et al., 2021), we created videos showing a shopping scene from a first-person perspective. Participants were instructed to imagine that they were this person. In the treatment groups, they were also instructed to imagine using an AR tablet that enables them to see additional product information as soon as they fixate a product. The video shows a supermarket shelf containing several boxes of cereal bars (Web-Appendix C for screenshots), and the person takes one box of chocolate cereal bars. The short video ended with the person holding the box in the hand. The degree of ARPI controllability was manipulated as follows. [1] *Control group without ARPI* (N = 70): The participants saw the box of cereal bars being taken from the shelf, but did not receive any additional information. [2] *Controllability, one round* (80): The participants saw three virtual text fields appear with the categories ecological, social, and regional hovering on the cereal bars box. They could choose one of the fields, and three related statements appeared. They could then pick a statement to which they received three details. [3] *Controllability, multiple rounds* (90): This condition consisted of three rounds. Each round was identical to the condition [2] (Controllability, one round), but at the end of rounds 1 and 2, the participants were referred to the starting screen to choose a category again. [4] *Controllability, multiple rounds with exit option* (70): This condition also includes three rounds, but in each step of the interaction the participants could indicate having received enough information and leave the ARPI. All product details were identical to the statements and details in the main study.

Next, the participants completed a short questionnaire. We measured brand image with three items as in the main study (M = 5.57, α = .91), and purchase intention with three items (M = 4.77, α = .95). As a more behavioral variable, we presented our focal brand and a competing brand. Box sizes were identical; the packaging design (colors, pictures) was very similar. The participants indicated on a seven-point scale whether they would rather buy the competing brand (1) or the brand presented in the ARPI (7) (M = 4.21). To operationalize the mediating mechanisms, the participants indicated their perceptions of the information’s comprehensiveness on the same scale as in the main study (M = 5.66, α = .91). As controls, we again measured product category knowledge (M = 4.08, ρ = .88) and sustainability attitude (M = 4.75, ρ = .89). Table 11 in Appendix 5 provides the sources, wording of items, and psychometric properties.

We gathered data of 310 U.S. consumers via Prolific. Their age ranged from 18 to 82 years, with a mean of 36.8 years (SD = 12.7); 41.6% are male, 58.1% female, and 0.3% diverse.

#### Results

We ran OLS regressions with dummy variables for the groups. Condition [4] (controllability, multiple rounds with exit option) imitates the main study’s controllable-detailed condition; therefore, we use this condition as contrast. The analyses show that all groups are assessed more positively than group [4]. This occurred for perceived comprehensiveness (group [1]: β = .425, t = 6.374, *p* < .001; [2]: β = .155, t = 2.290, *p* = .023; [3]: β = .245, t = 3.596, *p* < .001), brand image ([1]: β = .214, t = 3.069, *p* = .002; [2]: β = .140, t = 1.977, *p* = .049; [3]: β = .199, t = 2.783, *p* = .006), purchase intention ([1]: β = .239, t = 3.458, *p* < .001; [2]: β = .279, t = 3.989, *p* < .001; [3]: β = .179, t = 2.528, *p* = .012), and choice ([1]: β = .138, t = 1.962, *p* = .051; [2]: β = .158, t = 2.221, *p* = .027; [3]: β = .152, t = 2.114, *p* = .035). When entering the controls (sex, age, education, sustainability attitude, product category knowledge), the pattern of results remains (Table 6). Mediation analyses (Web Appendix C) confirm that perceived comprehensiveness mediates how the different types of the treatment shape brand image and purchase intention.

**Table 6:** Effect of different types of controllability (follow-up study 1)

	Comprehensiveness			Brand Image			Purchase Intention			Choice		
	β	t	p	β	t	p	β	t	p	β	t	p
Gender	.052	.963	.336	.032	.585	.559	.008	.146	.884	.027	.488	.626
Age	.037	.667	.505	−.004	−.069	.945	.031	.555	.580	.027	.473	.637
Sustainability Attitude	.074	1.247	.213	.219	3.562	<.001	.222	3.666	<.001	.265	4.336	<.001
Category knowledge	.065	1.073	.284	−.005	−.075	.940	.010	.159	.874	.007	.113	.910
Treamtment^1^												
- uncontrollable	.427	6.422	<.001	.219	3.184	.002	.242	3.582	<.001	.142	2.086	.038
- controllable 1 round	.143	2.122	.035	.128	1.832	.068	.267	3.889	<.001	.143	2.067	.040
- controllable 3 rounds no exit	.243	3.576	<.001	.191	2.719	.007	.173	2.494	.013	.144	2.065	.040
F	7.147			3.908			5.409			4.562		
R^2^	.142			.083			.111			.096		
R^2^adj	.122			.062			.091			.075		

OLS regression, β = standardized coefficients. ^1^ Treatment (dummy-coded): baseline = controllable, 3 rounds, exit option

#### Discussion

Follow-up study 1 confirms that the backfire effect of AR controllability arises due to reduced perceptions of information comprehensiveness. This negative effect, however, occurs mostly for higher degrees of controllability: when controllability is spread across multiple rounds and users have an exit option. Under these specific conditions, consumers realize that the cues of the richer medium are filtered out, and there was more information available such that they lack part of this information.

### Follow-up study 2: Low degree of controllability and further benefits of ARPI

#### Objective

This study elaborates the downstream consequences when applying lower degrees of information controllability (as established in follow-up study 1) and scrutinizes which positive effects on interaction quality inhibit or offset the backfire effect via perceived comprehensiveness. To advance our answers to H2, we include one round (category-statements-details) without an exit option as a low degree of controllability. Referring to H3, we expanded the interaction quality’s set of indicators as competing mediating processes. We test for variables that are relevant in AR settings, such as presence (Hilken et al., 2017; Javornik, 2016b), novelty (Yim et al., 2017), as well as utilitarian and hedonic benefit (Holdack et al., 2020; Rese et al., 2014). Moreover, this study compares the effect to that of a control group without ARPI. As another variation to increase the external validity, we use a video with AR glasses (Holdack et al., 2020; Rauschnabel et al., 2018) instead of AR tablets.

#### Design

We ran a 2 (controllable vs. uncontrollable) × 2 (detailed vs. nondetailed) between-subjects online experiment, adding a control group without an AR condition. We created videos for all five conditions, showing from a first-person perspective how a person takes one box of chocolate cereals from a shelf. Participants were instructed to imagine that they wear augmented reality glasses, which enable them to see additional product information as soon as they fixate a product. The experimental treatments were manipulated as follows. [1] *Uncontrollable-nondetailed* (N = 66): Three fields (ecological, social, regional) hovered on the cereals box. When participants clicked to proceed, they saw all three ecological statements; after another click, the ecological statements disappeared and three social statements appeared, followed by three regional statements. [2] *Uncontrollable-detailed* (86): This is the same as condition [1], except that the participants also saw three details for each statement. [3] *Controllable-nondetailed* (92): The participants could choose either the ecological, social, or regional field; afterward, a corresponding statement appeared. [4] *Controllable-detailed* (91): As in the third condition, the participants could choose one of the fields (ecological, social, or regional) to see the corresponding statement. Afterward, the participants could pick a statement to which they received a corresponding detail. [5] The *control group* (85) saw the video with the cereals box, but did not receive any additional information. The procedure is detailed in Web-Appendix D. The brand and all product details are identical to those used in the main study.

We again measured brand image (M = 5.40, α = .89), purchase intention (M = 4.29, α = .95), and as a more behavioral variable, choice of the focal brand over a competitor brand (M = 4.56). For the mediating mechanisms, the participants again indicated their perceptions of the information’s comprehensiveness (M = 5.16, α = .94), perceived credibility (M = 5.25, ρ = .92), perceived complexity (M = 2.15, ρ = .85), and perceived user friendliness (M = 4.75, α = .77). Extending the main study’s mediators, we added scales for presence (M = 4.43, α = .87) adopted from Huang and Liao (2015), novelty (M = 5.26, α = .82), utilitarian benefits (M = 4.42, α = .80) and hedonic benefit (M = 4.23, α = .92) taken from Voss et al. (2003). As controls, we again measured the self-reported product category knowledge (M = 4.75, ρ = .84) and sustainability attitude (M = 5.10, ρ = .81). Usage time was tracked (M = 67.47 s). Table 11 in Appendix 5 provides the sources, wording of items, and psychometric properties. We gathered data of 420 consumers via Prolific (M_age_ = 30.0 years, SD = 9.9; 57.9% of the participants are men, 41.2% female, and 1.0% diverse; 52.6% have a university degree).

#### Results

First, we contrasted the four experimental conditions against the control group without a treatment (group [5]). We ran OLS regressions, which show that all experimental groups produced a more positive brand image (group [1]: β = .201, t = 3.429, *p* < .001; [2]: β = .178, t = 2.936, *p* = .004; [3]: β = .143, t = 2.334, *p* = .020; [4]: β = .244, t = 3.990, *p* < .001). Moreover, purchase intentions ([1]: β = .122, t = 2.048, *p* = .041; [2]: β = .091, t = 1.473, *p* = .142; [3]: β = .059, t = .954, *p* = .341; [4]: β = .160, t = 2.591, *p* = .010) and preferences ([1]: β = .069, t = 1.167, *p* = .244; [2]: β = .130, t = 2.110, *p* = .035; [3]: β = .051, t = .817, *p* = .414; [4]: β = .144, t = 2.326, *p* = .020) were higher for half of the experimental groups compared to the control group.

In a second step, an OLS regression confirms the ARPI controllability’s influence on perceived comprehensiveness (β = −.397, t = −7.892, *p* < .001). The effect remains stable (β = −.388, t = −7.700, *p* < .001) when entering the control variables gender (β = −.006, t = −1.259, *p* = .209), age (β = −.060, t = −1.173, *p* = .242), education (β = −.098, t = −1.686, *p* = .093), product category knowledge (β = .042, t = .738, *p* = .481), and sustainability attitude (β = −.036, t = −.624, *p* = .533). For a full report, please consult the Web-Appendix D.

Finally, we ran mediation analyses (Table 12 in Appendix 6) that confirm the negative mediating influence of perceived comprehensiveness. Controllability decreases perceived comprehensiveness, which, in turn, influences the brand image and purchase intention. This effect can be found for both detailed and nondetailed information. For nondetailed information, there is also a mediating effect through perceived credibility. Adding to the main study, in the detailed conditions, there are further mediating effects of perceived complexity, perceived user friendliness, utilitarian benefits, and hedonic benefits.

#### Discussion

This follow-up study spotlighted lower degrees of controllability. Although the study confirms, again, information controllability’s negative influence on perceived comprehensiveness, in the condition of detailed information, there are also positive mediating effects via interaction quality, including (reduced) perceived complexity, user friendliness, utilitarian benefit, and hedonic benefit. Note that in contrast to the main study, we applied an online experiment to abstract the research model from the environmental setting of the point of sale. Presumably, due to this abstraction and the lower level of controllability, the positive mediators (known from online studies) are more pronounced. Still, our main finding that controllability reduces the perceptions of information comprehensiveness, which implies negative (countervailing) consequences for the marketing-relevant downstream variables, is replicated in this setting. Notably, contrasting ARPI against the group without ARPI supports the generally positive effect of the ARPI.

### Follow-up field study 3: ARPI effect on actual purchases in a whole product category

#### Objective

In the final stage of our research, we explored, in two follow-up studies (3 and 4), the moderating role of rush hour (H4) because this boundary condition carries strong managerial implications for retailers. We ran another study in a field setting that employed a more objective criterion (the number of customers purchasing in a given period). The second objective is to contrast ARPI users with regular shoppers based on hard data taken from check-out scanners. Third, our previous studies focused on only one brand in one product category. This study considered another product category with several product types and brands. We did not manipulate the ARPI design; instead, we focused on the critical controllable-detailed information condition. In the main study, we found the backfire effect to occur in this condition in busy shopping times.

#### Design

We ran the study in the same hypermarket as in the main study. We used an ARPI app that we designed exclusively for this study to give information about beer products. The participants could scan the product’s front or entire beer boxes to access the ARPI. The ARPI recommends other beer products that fit the customers’ preferences (see Web-Appendix E for a description). The hypermarket in which we ran the study offers 316 different items in the beer category (different types, brands, and containers, such as bottles and boxes), which we included in the app (we excluded cans and barrels). The price per bottle ranged from €0.29 to €2.79 for craft beer. Bottle sizes ranged from 0.2 to 1.0 l. The study covered a wide range of beer types, including non-alcoholic beer and beer-mixed drinks.

We collected the data over three weeks. As in the main study, we balanced the treatments across daytime slots and weekdays. Interviewers approached shoppers in the hypermarket’s beer section (opening claim: “Find out more about your beer!”). Most shoppers agreed to participate, and the interviewers briefly showed them the app’s basic functionality; shoppers used the app without an interviewer present to ensure that the shoppers made their choice at their own pace.

To determine the ARPI effect, we contrasted the ARPI users with the scanner data of the entire population, i.e., all other consumers who bought beer during the time that we ran the study, as well as about one week before and two weeks later (n = 10,657). We received usable data from 51 subjects who used the AR. The instructors guessed and coded the participants’ age (≤ 30 years: 41.9%, 31–50 years: 32.5%, > 50 years: 25.6%) and gender (male: 25.6%, female: 39.5%, male/ female couples: 34.9%). We applied the app’s tracking data and check-out scanner data. The AR tracked the time shoppers used the device. The subjects used the app for 86.1 s (SD = 90.7). Excluding the time for reading the instruction screen, the net usage time was 66.9 s (SD = 41.0). Having finished their shopping, we asked the shoppers to hand in their shopping receipts.

We coded the rush hours and more relaxed shopping hours as in the main study. As an additional measure, we coded the crowding in the retail area from the scanner data. For this proxy, we determined the number of customers in the hypermarket who purchased within 30 min (M = 17.77, SD = 9.70), ranging from one to a maximum of 60 purchasing customers.

#### Results

More than half of the ARPI users (53.2%) bought the beer they initially planned (beer type *and* brand), meaning that 46.8% deviated from their first choice. As another indication of ARPI’s potential to shift consumption patterns, 61.7% bought the recommended brand, although they may have switched the beer type (note that several users bought more than one brand or item). Likewise, 63.8% bought the recommended type, although they might have changed the brand. In sum, 55.3% bought exactly the beer (beer type *and* brand) the ARPI recommended.

To assess whether the ARPI resulted in more and different purchases, we compared the scanner data for the ARPI users with the scanner data for the population who purchased beer in the same period. The ARPI led to a notable shift and extension in consumers’ choice sets. The most popular and commonly purchased beer type sold in the hypermarket is pilsner. The share of consumers who bought only pilsner decreased from 29.1% to 23.5%, while the share of those who combined pilsner with other beer types when buying different types at the same time increased from 10.0% to 21.6% (χ^2^_(1)_ = 6807.325, *p* < .001). The average number of bottles of other beer types (not pilsner) per shopper almost doubled (15.7 vs. 7.9, + 98.6%, t_(10,706)_ = 4.150, *p* < .001, Cohen’s d = .583).

**Fig. 5:**
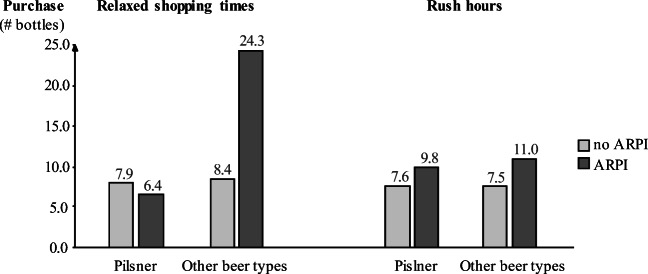
Shift in beer types based on ARPI usage and busy shopping times (follow-up study 3)

**Fig. 6:**
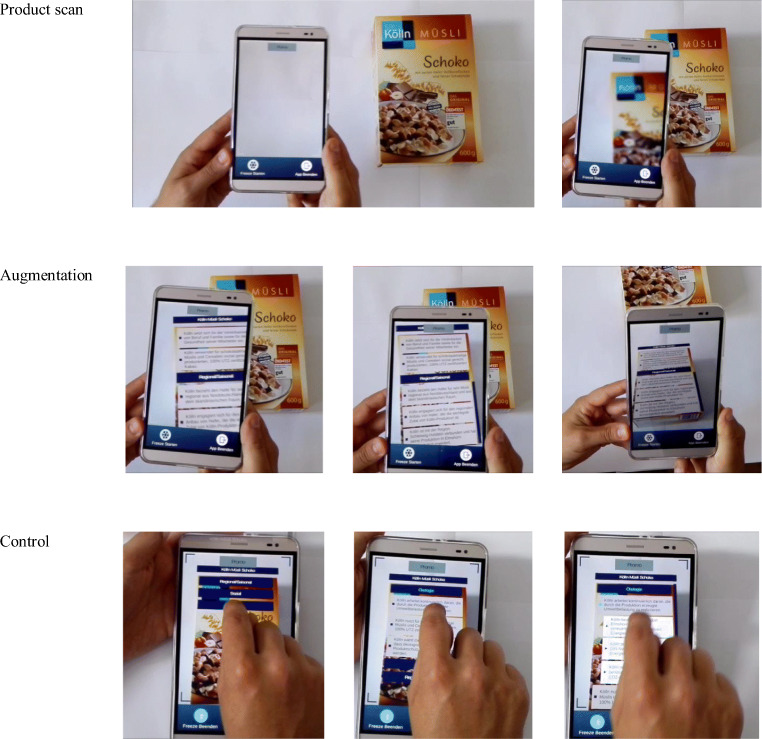
Visualization of the ARPI function (main study)

**Fig. 7:**
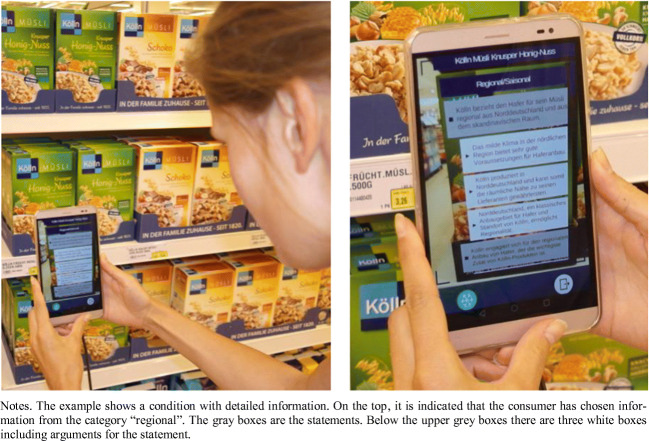
The ARPI Provided in a tablet at the point of sale (main study). Notes. The example shows a condition with detailed information. On the top, it is indicated that the consumer has chosen information from the category “regional”. The gray boxes are the statements. Below the upper grey boxes there are three white boxes including arguments for the statement

As shown in Fig. 5 and supporting the main study’s findings regarding H4, the ARPI usage’s effect in shifting consumption greatly varies across rush hours and more relaxed shopping hours. There is a large ARPI influence on the number of bottles of other beer types (not pilsner) that consumers buy (ANOVA: main effect ARPI: F_(1,10,704)_ = 24.315, *p* < .001, main effect rush hour F_(1,10,704)_ = 13.182, *p* < .001, interaction: F_(1,10,704)_ = 10.011, *p* = .002). The ARPI only helped increase the variety of bought beer types during relaxed shopping times. Note that there is no effect on the number of pilsner bottles bought during rush hours (main effect ARPI: F_(1,10,704)_ = .027, *p* = .870, main effect rush hour F_(1,10,704)_ = .381, *p* = .537, interaction: F_(1,10,704)_ = .548, *p* = .459).^9^ This interaction effect also occurs when applying the more objective operationalization of rush hour, namely crowding (interaction: B = −.496, t = 2.320, *p* = .020). The positive ARPI-effect only occurs when fewer (9) or average (16) numbers of customers are in the market (both *ps* < .001), but turns insignificant when many (27) customers were shopping in the 30 min period (*p* = .272).

As a robustness check, we also conducted propensity score matching on a large set of shopping characteristics detailed for certain product categories (e.g., amount of and spending on purchased food products; Web-Appendix E). These checks support the managerial relevance of the AR technology and the importance of consumer stress in busy shopping times.

#### Discussion

With hard scanner data, this study substantiated that ARPI can influence purchase behaviors at the point of sale. The ARPI was found to shape consumer decisions, as more than half of the ARPI users bought another beer type and/or brand than initially planned. Most importantly, the study further supported H4 and the adverse effects in busy shopping times.

### Follow-up lab study 4: Experimental manipulation of rush hour

#### Objective and design

The results of the main study imply that in rush hours, the backfire effect is particularly prominent, the more detailed the accessible information is. Thus far, we explored this critical contextual factor through proxies, such as the daytime (main study) or the number of purchasers in a given period (follow-up study 3). To gain further insights, we manipulated this variable in a two-level (rush hour vs. no rush hour) experiment in a fashion lab store of a French business school (133 students, 20.03 years, 53% females), where participants used an AR device to gain access to additional (sustainability-related) product information. Participants searched for a fashion item as a present for a friend. The focal product’s brand provides additional information on the item (a sweater) and the labels. This information was, however, shown in another language (German, in this case). To access the additional product information, participants used a tablet with an AR app that allows translations in real time. As for the ARPI in the main experiment, the AR function thus showed the additional (translated) information directly on the product. Participants used the AR device in both experimental conditions for the same time, but with vs. without being rushed. In the rush hour condition, the participants were informed that they are “in a hurry because the next customer wants to look at the product too” and pop-up messages reinforced this aspect (see Web-Appendix F for a more detailed description of the study design). This manipulation was successful, as participants experienced greater stress in this condition (one-tailed testing, M_no rush_ = 2.61; M_rush_ = 4.42, t = 5.715, *p* < .001). This study zooms in on whether rush hour favors more nondetailed information; therefore, we assessed how comprehensive the participants perceive the information to be. Since the study instructions ask the participants to imagine that they intend to purchase the sweater, we used their willingness to pay as a purchase-relevant variable in this study.

#### Results

Regression analysis (one-tailed) shows a significant interaction effect of rush hour and information comprehensiveness on willingness to pay (B = −19.34, t = −1.698, *p* = .046). AR use while being rushed had no implications when participants perceive the accessed information to be less comprehensive (B_-1SD_ = 6.89€, t = .431, *p* = .333) or average (B_mean_ = −12.45€, t = −1.101, *p* = .137). However, AR use while being rushed reduces willingness to pay by around €32 when participants perceive this information to be more comprehensive (B_+1SD_ = −31.80€, t = −1.974, *p* = .025). To ensure that this shift is not driven by outliers, we reran the test with ranks, or when eliminating extreme willingness to pay, with the same results. Furthermore, we assessed brand image for which we find a similar negative shift (interaction: B = −.34, t = −2.316, *p* = .011).

#### Discussion

This final experiment confirmed the key role of rush hour as a context factor for ARPI. Confirming our main study and follow-up study 3, the use of AR while being rushed hampers marketing outcomes (e.g., willingness to pay), especially when consumers perceive the accessible product information as being overly comprehensive. This liability in rush hours does not arise if consumers perceive the accessed information to be less comprehensive.

## Discussion and contribution to theory

This research demonstrates that AR technology has evolved from a toy to a tool that can guide shopping decisions in brick-and-mortar stores. Guided by our theoretical reasoning, the series of three pre-studies as well as a main study in a field setting and four follow-up studies confirm our proposed backfire effect and provide several novel insights into how consumers respond to ARPI in retail environments. The paper extends past AR research in several manners and, most importantly, contributes to developing an ARPI-specific theory.

First, this is the first approach to lay the theoretical grounds specifically for the application of AR in brick-and-mortar stores. Past literature mainly considered the technology in e-commerce (Table 7 in Appendix 1), where the main AR functionalities are trying-on and placing and the AR is basically used for hedonic and experiential reasons. Accordingly, past research has adopted theories, such as the technology acceptance model (Venkatesh et al., 2003), to explain the adoption of the technology through the perceived ease of use and the perceived enjoyment (e.g., Rese et al., 2014, 2017), or the flow theory (Novak et al., 2003) to explain the user experience via presence and flow experience (e.g., Barhorst et al., 2021). This paper widens the scope beyond experiential AR usage in e-commerce (Javornik, 2016b; Pantano et al., 2017; Rese et al., 2017) to informing consumers in offline retail settings, and concludes that specific theoretical approaches are needed here. As such, we add to the literature on in-store technologies (Grewal et al., 2020) and self-service technologies (Evanschitzky et al., 2015). As demonstrated in the pre-studies, consumers in these settings ask for utilitarian instead of hedonic benefits, as they are primarily interested in the information provided. Accordingly, a specific theoretical approach beyond the ones applied in e-commerce settings is needed. While the few empirical studies conducted in physical settings highlight AR’s positive effects on the evaluation of the information provided and the (re)use of the technology (Spreer & Kallweit, 2014), this paper is the first to comprehensively explain and empirically confirm ARPI’s impact at the point of sale on downstream marketing variables, such as brand image and purchase intention.

Second, our theory development includes the design of the ARPI as a key predictor of its effectiveness. While extant research has only compared ARPI with a control group without additional information (Joerß et al., 2021; Spreer & Kallweit, 2014), this paper theoretically discusses and empirically confirms that the technology’s effectiveness depends on how the ARPI is designed in terms of information controllability and detailedness. Although consumers generally welcome additional information, too much of it leads to cognitive overload (Hu & Krishen, 2019; Roetzel, 2019). The controllability of AR-delivered information may be a feasible solution to overcome this trade-off. AR technology enables interactivity with digital supplements such that consumers select the pieces of information that are of interest to them. However, we show that controllability is a double-edged sword. Integrating media richness theory (Daft & Lengel, 1986) and cues-filtered-out theory (Sproull & Kiesler, 1986) into AR research provides a major contribution to theory development in this growing area. Despite certain positive effects, information controllability also bears the danger of a backfire effect because users may have the impression of not being comprehensively informed. Notably, this backfire effect of information controllability under the condition of detailed information cannot be derived directly from media richness theory, because this reasoning would actually make more favorable predictions in the controllability and detailedness condition. By borrowing knowledge from the literature on the fear of missing out (Tandon et al., 2021, 2022), we were able to theoretically explain this new, unexpected AR-specific backfire effect. The follow-up studies further help disentangle this effect, as they reveal that the degree of controllability is relevant. The backfire effect is particularly strong, if consumers can decide by themselves when to stop using the ARPI. Evidently, the fear of missing out on information appears to be strongest under higher degrees of ARPI controllability.

Furthermore, this research introduces the concept of rush hour into the AR literature. This concept provides a theoretically rich and practically relevant boundary condition for in-store technologies. Building on the field theory (Lewin, 1939) and past literature of the stress-evoking aspects of retail crowding (Baker & Wakefield, 2012; Lucia-Palacios et al., 2018; Santini et al., 2020), we include the rush hour as an amplifier of the ARPI backfire effect in our theory. When shoppers are stressed and distracted by the retail crowding, they are unable to exploit the benefit of self-selecting the AR-delivered information. The rush hour concept once again demonstrates that a specific ARPI theory for brick-and-mortar retailing is needed. AR applications in e-commerce do not suffer from such crowding effects. For this reason, the existing broad body of literature on AR has not yet explored this relevant moderating influence.

In sum, this paper suggested and empirically validated ARPI-specific effects in brick-and-mortar retail to advance our theoretical knowledge about this important AR application, which will become even more widespread in retailing in the future. The new theory will, hopefully, guide AR designers, marketers, and retailers when they develop and implement new ARPI applications and it will hopefully stimulate more research in this evolving field.

### Implications for marketers and retailers

This study provides evidence-based knowledge in the substantive domain of ARPI in brick-and-mortar retailing. Since we conducted the main study in the field in a hypermarket, the findings are highly ecologically valid and of practical relevance. The multi-study approach contributed to generalizability and established a substantial contribution by testing the ARPI in various settings including different products and brands, AR devices, and product information content. The practical knowledge gained in the series of experiments relates to four central aspects, which we structure along the acronym ARPI: **A**ugmented reality effectiveness, **R**etailing channel and the corresponding AR functionalities, **P**roductive design, and **I**ntegration in the context.

#### Augmented reality effectiveness

ARPI can augment the limited physical space with digital supplements and create virtual space that provides almost unlimited information compared to traditional means of communication. This research has demonstrated that ARPI offers the potential to shape marketing outcomes, such as brand image and purchase intention. As the pre-studies show, AR technology outperforms other marker-based sources of information, such as QR codes, because it creates a stronger tie between product and information in the consumer’s mind. ARPI is, therefore, the preferable tool to provide information at the point of sale when the physical space is limited. However, marketers and retailers need to be aware of the backfire effect when the AR is controllable and lots of detailed information is principally accessible. This is important to stress, because this drawback is ARPI specific. In traditional media (e.g., booklets), the body in which the information chunks are embedded serves as a peripheral cue. In the digital world, consumers arguably have greater difficulty gathering knowledge (consciously or unconsciously) about the realm of accessible information. The amount of information that is accessible via ARPI is less salient to them. While the use of AR is generally recommended, its superiority over more traditional communication methods should thus be checked before implementing the technology.

#### Retailing channel and the corresponding AR functionalities

Using AR in retailing provides several functionalities including informing, placing, and trying-on. These functionalities offer different benefits for different retailing channels. Contrasting the present research with the extant AR studies in e-commerce (Table 7 in Appendix 1) pinpoints that the ARPI functionality of informing in physical stores differs from the characteristic AR functionalities of placing and trying-on in e-commerce. ARPI designers, marketers, and retailers should be well aware that the hedonic benefit is more relevant for AR applications in e-commerce. However, our pre-studies revealed that ARPI users are guided by AR’s utilitarian benefit instead. In physical retailing settings, marketers and retailers should therefore ensure that the AR technology provides relevant details and facts, which support the consumption decision, instead of trying to entertain the shopper.

#### Productive design

This research has demonstrated that ARPI will only be effective if marketers and retailers design their applications carefully. The risk that ARPI controllability backfires is contingent on other design elements, such as the detailedness of information. Our dual framework and the empirical investigations emphasize the existence of two countervailing mechanisms that the ARPI’s controllability can activate. Especially for fairly nondetailed information, the controllability stimulates positive inferences about information credibility, with even favorable consequences for buying intentions and brand image. If retailers seek to augment concise information, they may thus use a controllable approach. Conversely, when providing detailed information, AR controllability more likely provokes perceptions that relevant cues are filtered out. ARPI designers should therefore include elements that offer relevant cues to the available information’s comprehensiveness. Alternatively, retailers may limit the possibility of filtering out too many details to avoid harming perceived information comprehensiveness. Not allowing consumers to exit at any time could help as well. Although not tested within our research, certain design elements, such as content overviews and visualizations of the content structure, may help.

#### Integration in context

In times of retail crowding and when shoppers are more stressed (Monday–Friday afternoons and Saturday mornings), retailers are well advised to avoid delivering detailed information in a controllable fashion. For consumers in a hurry, concise information via AR is helpful, while more detailed information is not helpful if the AR is controllable. In more relaxed shopping situations, consumers are more open to consider additional details, which ultimately feeds into their decisions. Retailers may consequently adjust the AR application to the specific situation or might take measures to nudge a certain shopping mood in rush hours before equipping consumers with ARPI. Various ambient measures, such as slow music or relaxing lighting, could help consumers slow down (Biswas et al., 2017, 2019). Beyond adjusting ARPI according to daytimes and weekdays, setting the ideal default for controllability and detailedness may be best to improve ARPI effectiveness. Goldstein et al. (2008) propose a decision tree for different defaults that can be adopted for the ARPI design. If individual preferences are known, the AR app could use the customer’s ideal setting as default (persistent default). If information is available for similar customers only (target group), a smart default adjusting to the target group preferences can be applied. If no customer information is available, adjusting the design concerning daytime and weekdays could be a benign default. In the long run, integrating ARPI usage data and scanner sales for machine learning should help continuously optimize the design.

## Limitations and future research directions

Our study provides several avenues for AR research. First, ARPI can be a feasible tool to propel brick-and-mortar stores into the digital age. Still, we call for more evidence about the generalizability of the findings, and research needs to extend our approach. While we tested ARPI for cereals, beer, and fashion products, the tool is transferrable to other consumption types and shopping fields. With regard to the inhibiting effect of consumer stress in rush hours, we expect ARPI effectiveness to be even greater for shopping goods, such as furniture or consumer electronics, because consumers usually take more time to search for and compare these goods than when buying groceries in habitual routines. Even for products that shoppers buy habitually, the ARPI may shape the consumption decision if consumers can be encouraged to use the technology: Scanning their favorite product, consumers may discover alternative options or may be motivated to try other products in response to the ARPI (see follow-up study 3).

Second, our research has shown that the shopping times serve as a boundary condition. This effect is due to the higher retail crowding and the induced shopping stress (Lucia-Palacios et al., 2018; Santini et al., 2020). In these situations, consumers will consider shopping as work instead of as fun (Babin et al., 1994). We call for research to scrutinize the relationships among shopping time, crowding effects, and shopping values in future field studies. Relatedly, we recommend that researchers apply and compare different measures of the rush hour. In our research, we used pre-specified rush hours (Irmak et al., 2020), the number of shoppers extracted from scanner data, and we manipulated the retail crowding in an experimental study. In future research, scholars could also observe the number of shoppers per square meter or measure the perceived retail crowding via questionnaires (Baker & Wakefield, 2012). This stream of research could also consider how habituation of social distancing and increased hygiene concerns during the COVID-19 pandemic have affected the stress level induced by retail crowding. While this development has arguably reduced the number of customers in many shops, thereby potentially dampening the stress level, the actual presence of other waiting consumers might even raise the stress level further, leading to more extreme contexts for ARPI usage.

Third, the fear of missing out due to the reduced perceived information comprehensiveness could be subject to future investigation (Tandon et al., 2021, 2022). Furthermore, it would also be interesting to consider this construct as a trait to explore its moderating role.

Fourth, consumers who are generally open to novel technologies should tend to adopt ARPI early (Blut & Wang, 2020). We expect technological savviness, cognitive openness, and product involvement to determine how consumers react to the new technology. From a more long-term perspective on the diffusion of ARPI, we expect cohort effects, with digital natives being more open to electronic information supplements in analogous stores. Surely, ARPI is an innovative concept and consumers’ readiness to use it will change over time. Since the learning curve is presumably steep, longitudinal studies are required. Moreover, research on personal shopping assistants has shown that the factors influencing initial and repeated usage differ (Evanschitzky et al., 2015). We expect similar developments for how consumers use ARPI at the point of sale.

Fifth, it would be interesting to test the ARPI effects for different AR-enabling devices. Today’s consumers are accustomed to smartphones and tablets. Carrying the tablet and using the AR function for a specific product is, however, not what the customers would naturally do. Furthermore, the displays of tablets and phones are relatively small, which may have been another uncontrolled source of the backfire effect (although perceived complexity is actually reduced for controllable ARPI). Head-mounted displays, AR glasses, or even contact lenses could create a more natural shopping experience that allows consumers to use their hands (Flavián et al., 2019; Rauschnabel et al., 2019). These devices are not common yet in everyday experience, but once they are more widespread, retailers can apply them in their shops even for grocery shopping. The ARPI effects observed in this research may become even stronger as these devices are more subtly integrated into everyday life (e.g., similar to wearing regular glasses). Please note that pre-study 2 (Web-Appendix B) reports initial evidence indicating that ARPI glasses exert stronger effects than tablets. More research on the different AR devices at the point of sale is therefore required.

### Supplementary Information

ESM 1 (DOCX 5089 kb)

## Appendix 1

**Table 7:** AR in e-commerce literature overview

		Study type			Data collection			AR feature			Category	Main finding
		survey	experiment	scanner	on-line	lab	field	try-on	planner	infolayer		
Kim and Forsythe (2008)		■			■			■			apparel	Perceived usefulness and perceived entertainment improve attitude toward using, which in turn increases intended use and post-use evaluations.
Rese et al. (2014)		■				■				■	furniture	Components of the TAM can predict the intention to use a mobile AR catalogue app. Both perceived informativeness and perceived enjoyment increase the intention to use.
Huang and Liao (2015)		■			■			■			apparel	Consumers with high cognitive innovativeness emphasize usefulness, aesthetics, and service excellence, while consumers with low innovativeness emphasize playfulness and ease of use.
Javornik (2016b)			■			■		■	■		furniture, sunglasses	Flow mediates the effects of perceived augmentation on consumers’ affective responses and behavioral intentions.
Pantano et al. (2017)		■				■		■			sunglasses	Ease of use, enjoyment, and perceived usefulness jointly increase attitude towards and the intention to use a virtual mirror. Aesthetic quality and interactivity influence ease of use and enjoyment, response time and quality of information increase perceived usefulness.
Rese et al. (2017)		■				■		■		■	furniture, news, glasses, apparel	Components of the TAM predict the intention to use an AR catalog app. The relative importance of hedonic and utilitarian aspects depends on the type of AR app (marker-based vs. markerless).
Poushneh and Vasquez-Parraga (2017)			■			■		■			apparel; sunglasses	AR influences user experience and user experience in turn affects satisfaction and willingness to buy.
Yim et al. (2017)			■		■			■			sunglasses, watches	AR (compared to conventional websites) fosters immersion, which results in enjoyment, usefulness, positive attitude towards the medium, and purchase intention. Effects are stronger for consumers with low (vs. high) previous media experience
Hilken et al. (2017)			■			■		■			glasses, cosmetics	The AR-enabled interaction of simulated physical control and environmental embedding positively affects value perceptions. Spatial presence functions as a mediator and also predicts decision comfort. Customer value perceptions and decision comfort translate into positive behavioral intentions.
Beck and Crié (2018)			■		■			■			apparel, glasses	AR (virtual mirror) on a website increases curiosity, intention to patronize, and intention to purchase (online and offline).
Poushneh (2018)			■			■		■		■	sunglasses, cars, entertainment	Augmentation quality and user’s control of access to personal information enhance user satisfaction.
Rauschnabel et al. (2019)		■				■			■		furniture, music video	The effects of benefits from AR apps on brand attitude are mediated by consumer inspiration.
Yim and Park (2019)					■			■			sunglasses	Consumers with an unfavorable body image evaluate AR try-ons better than traditional Web-based product presentations. The effects of interactivity and media irritation on adoption intention are moderated by body image for AR but not for Web-based product presentations.
Heller et al. (2019)			■		■	■		■	■		furniture, toys, food	AR-enabled frontline affects positive WOM and product choice. The effect is mediated by processing fluency and decision comfort and moderated by processing-type and product contextuality.
Whang et al. (2021)			■		■			■			cosmetics	The effect of the AR experience on purchase intention is mediated by cognitive control. Peer’s opinion moderates the effect of AR on cognitive control.
Tan et al. (2021)				■			■	■			cosmetics	An international cosmetic retailer’s mobile AR app is associated with higher sales for less popular brands, products with a narrower appeal, and more expensive products. Findings indicate that AR is most effective when product-related uncertainty is high.

## Appendix 2

**Table 8:** Alternative mediators

Potential alternative mediator	Rationale
Perceived credibility	The effects of ARPI controllability on downstream marketing outcome variables might by mediated by perceived credibility. If the provider empowers consumers to choose the delivered information freely, consumers may also be less likely to develop the impression that the provider attempts to direct them to a specific piece of information or even that the provider tries to influence their decisions. Consumers will consider controllable information delivery as more credible than uncontrollable means of conveying information. This positive effect might spill-over to the brand image and purchase intention.
Perceived complexity	The effects of ARPI controllability on downstream marketing outcome variables might by mediated by perceived complexity. Controllability could decrease the perceived complexity, as consumers can focus on and pick the input that is relevant for them and filter the less relevant aspects (Mai et al., 2014). If consumers perceive the ARPI as less complex, the brand image and purchase intentions may increase.
Perceived user friendliness	The effects of ARPI controllability on downstream marketing outcome variables intention might by mediated by perceived user-friendliness. ARPI controllability should enhance perceptions of user friendliness (Rese et al., 2014, 2017). This positive effect might spill-over to the brand image and purchase intention.
Presence	The effects of ARPI controllability on downstream marketing outcome variables intention might by mediated by presence. Extant studies on AR in e-commerce have shown that AR increases presence (Hilken et al., 2017; Javornik, 2016b). This positive effect might spill-over to the brand image and purchase intention.
Novelty	The effects of ARPI controllability on downstream marketing outcome variables might by mediated by novelty. Past AR research in the e-commerce context confirmed that AR increases novelty (Yim et al., 2017). This positive effect might spill-over to the brand image and purchase intention.
Utilitarian benefit	The effects of ARPI controllability on downstream marketing outcome variables might by mediated by utilitarian benefit. Past studies on AR in e-commerce demonstrated that AR increases perceived usefulness and the utilitarian benefit (Holdack et al., 2020; Rese et al., 2014). This positive effect might spill-over to the brand image and purchase intention.
Hedonic benefit	The effects of ARPI controllability on downstream marketing outcome variables might by mediated by perceived credibility. Existing AR research in the e-commerce context supported that AR increases perceived enjoyment and hedonic benefit (Holdack et al., 2020; Rese et al., 2014). This positive effect might spill-over to the brand image and purchase intention.

## Appendix 3

**Table 9:** Overview of studies

	ARPI control		ARPI content		ARPI context				Consumer processing			Response		Design		
					device	other media	rush hour		comprehen.	interaction quality				location	N	product
Pre-study 1^a^	no ARPI / uncontrollable		nondetailed		glasses	CG				utilitarian / hedonic benefit		purchase intention		web	469	cereals
Pre-study 2^a^	no ARPI / uncontrollable		detailed		tablet, glasses	QR, CG				utilitarian / hedonic benefit		brand image, purchase intention		web	308	cereal bars
Pre-study 3^a^	controllability demonstrate		detailed		tablet	QR				utilitarian / hedonic benefit, presence, information-product-fit		brand image, purchase intention		web	260	cereals
Main study	uncontrollable / controllable^b^		nondetailed / detailed		tablet	booklet	relaxed / rush hour		comprehensiveness	complexity, user friendliness, credibility		brand image, purchase intention purchases^e^		field	403	cereals
Follow-up 1	no ARPI / controllable^b,c,d^		detailed		tablet	CG			comprehensiveness			brand image, purchase intention choice^f^		web	310	cereal bars
Follow-up 2	no ARPI / uncontrollable / controllable^d^		nondetailed / detailed		glasses	CG			comprehensiveness	complexity, user friendliness, credibility, presence, novelty, utilitarian / hedonic benefit		brand image, purchase intention choice^f^		web	420	cereals
Follow-up 3	no ARPI / controllable^b^		detailed		tablet	CG	relaxed / rush hour					scanner^g^		field	51	beer
Follow-up 4	not manipulated		not manipulated		tablet		manipulated (relaxed / rush hour)		comprehensiveness			willingness to pay		lab	133	fashion

^a^Pre-studies are reported in the Web-Appendix A. controllability: ^b^ multiple rounds with exit, ^c^ multiple rounds without exit, ^d^ one round. Other media: CG = control group without ARPI. Response: ^e^self-reported purchases, ^f^preference over competing brand, ^g^check-out scanner data of purchases. Location: field: ARPI-tablet in hypermarket, web: online-experiment showing ARPI video from a first-person perspective

## Appendix 4 Design of the main study.

**Table 10:** Structure of the sustainability content (main study)

	**Chocolate**			**Fruits**			**Honey-Nut**		
	Ecological	Social	Regional	Ecological	Social	Regional	Ecological	Social	Regional
*Statement*	*S*_*c,e1*_	*S*_*c,s1*_	*S*_*c,r1*_	*S*_*f,e1*_	*S*_*f,s1*_	*S*_*f,r1*_	*S*_*h,e1*_	*S*_*h,s1*_	*S*_*h,r1*_
Detail	D_c,e1_a D_c,e1_b D_c,e1_c	D_c,s1_a D_c,s1_b D_c,s1_c	D_c,r1_a D_c,r1_b D_c,r1_c	D_f,e1_a D_f,e1_b D_f,e1_c	D_f,s1_a D_f,s1_b D_f,s1_c	D_f,r1_a D_f,r1_b D_f,r1_c	D_h,e1_a D_h,e1_b D_h,e1_c	D_h,s1_a D_h,s1_b D_h,s1_c	D_h,r1_a D_h,r1_b D_h,r1_c
*Statement*	*S*_*c,e2*_	*S*_*c,s2*_	*S*_*c,r2*_	*S*_*f,e2*_	*S*_*f,s2*_	*S*_*f,r2*_	*S*_*h,e2*_	*S*_*h,s2*_	*S*_*h,r2*_
Detail	D_c,e2_d D_c,e2_e D_c,e2_f	D_c,s2_d D_c,s2_e D_c,s2_f	D_c,r2_d D_c,r2_e D_c,r2_f	D_f,e2_d D_f,e2_e D_f,e2_f	D_f,s2_d D_f,s2_e D_f,s2_f	D_f,r2_d D_f,r2_e D_f,r2_f	D_h,e2_d D_h,e2_e D_h,e2_f	D_h,s2_d D_h,s2_e D_h,s2_f	D_h,r2_d D_h,r2_e D_h,r2_f
*Statement*	*S*_*c,e3*_	*S*_*c,s3*_	*S*_*c,r3*_	*S*_*f,e3*_	*S*_*f,s3*_	*S*_*f,r3*_	*S*_*h,e3*_	*S*_*h,s3*_	*S*_*h,r3*_
Detail	D_c,e3_g D_c,e3_h D_c,e3_i	D_c,s3_g D_c,s3_h D_c,s3_i	D_c,r3_g D_c,r3_h D_c,r3_i	D_f,e3_g D_f,e3_h D_f,e3_i	D_f,s3_g D_f,s3_h D_f,s3_i	D_f,r3_g D_f,r3_b D_f,r3_i	D_h,e3_g D_h,e3_h D_h,e3_i	D_h,s3_g D_h,s3_h D_h,s3_i	D_h,r3_g D_h,r3_h D_h,r3_i

S – Statement (given in the detailed and nondetailed condition), D – Detail (only given in the detailed condition). Indices: c – chocolate, f – fruits, h – honey-nut, content of information: e – ecological, s – social, r – regional, number (1–3) indicate the statement, letters indicate different details

Example: A statement for social engagement read: “The company supports and ensures a good work-life balance and is concerned about the health of all co-workers.” At the more detailed level are arguments supporting the statements, such as “The company offers several work time models to facilitate the re-entry of co-workers after parental leave.”

## Appendix 5

**Table 11:** Multi-item-scales

Construct	Indicators	Main Study				Follow-up 1				Follow-up 2		
		λ	AVE	r^2^_max_		λ	AVE	r^2^_max_		λ	AVE	r^2^_max_
Brand image ^a)^			*.885*	*.329*			*.768*	*.490*			*.732*	*.498*
	I like XY.	.804				.846				.835		
	XY stands for quality.	.806				.889				.862		
	I trust in XY.	.921				.894				.870		
Purchase intention ^b)^							*.874*	*.490*			*.875*	*.498*
	It is very likely that I will buy the product shown.					.943				.957		
	I have great interest to buy the product shown.					.921				.941		
	I will buy the shown product in the future.					.894				.908		
Perceived comprehensiveness ^c)^	*For me, the provided information was …*		*.708*	*.314*			*.731*	*.311*			*.791*	*.289*
	detailed	.842				.936				.868		
	broad	.805				.706				.849		
	deep	.881				.911				.910		
	comprehensive	.837				.849				.928		
Category knowledge ^d)^	*Compared to other people,*		*.799*	*.323*			*.760*	*.238*			*.731*	*.289*
	I think intensively about food products.	.951				.871				.764		
	I know a lot about food products.	.701				.872				.937		
Sustainability attitude ^e)^			*.724*	*.329*			*.785*	*.238*			*.745*	*.289*
	Overall, I am an environmentally friendly person.	.887				.836				.837		
	Overall, I consume in a sustainable manner.	.813				.933				.888		
Perceived credibility ^f)^	*For me, the provided information was …*		*.811*	*.323*							*.858*	*.460*
	trustworthy	.908								.930		
	credible	.893								.923		
Perceived complexity ^g)^	*For me, the provided information was …*		*.606*	*.048*							*.740*	*.530*
	complex	.782								.859		
	confusing	.775								.861		
Perceived user friendliness ^h)^	*For me, the provided information was …*		*.558*	*.193*							*.543*	*.530*
	user-friendly	.738								.813		
	appealing	.628								.583		
	well-arranged	.875								.797		
Utilitarian benefit ^i)^	*I find the shopping experience to be ...*										*.601*	*.566*
	functional									.794		
	necessary									.632		
	practical									.793		
Hedonic benefit ^i)^	*I find the shopping experience to be ...*										*.767*	*.566*
	fun									.847		
	exciting									.878		
	thrilling									.902		
Presence ^j)^											*.709*	*.191*
	I had a sense of being in the scenes displayed									.932		
	I felt I was visiting the places in the displayed environment									.897		
	I felt that the characters and/or objects could almost be touched									.674		
Novelty ^k)^	*Using the augmented reality feature offers …*										*.619*	*.448*
	novel information									.749		
	unique information									.775		
	specific content									.833		

Source: Scales adopted or adapted from ^a)^ Hoffmann & Mueller (2009), Mai et al. (2014), ^b)^ Dodds et al. (1991), ^c)^ Rese et al. (2014, 2017), Mai et al. (2014), ^d)^ Chang (2004), ^e)^ Whitmarsh & O’Neill (2010), ^f)^ Sengupta and Johar (2002), ^g)^ Geissler et al. (2001), ^h)^ Srinivasan et al. (2002), ^i)^ Voss et al. (2003), ^j)^ Huang and Liao (2015), ^k)^ Yim et al. (2017). Confirmatory factor analysis (AMOS 25.0): Study 1: χ^2^_(115)_ = 198.61, χ^2^/d.f. = 1.73; CFI = .98; RMSEA = .04; follow-up study 1: χ^2^_(67)_ = 130.20, χ^2^/d.f. = 1.94; CFI = .98; RMSEA = .05; follow-up study 2: χ^2^_(419)_ = 768.85, χ^2^/d.f. = 1.79; CFI = .96; RMSEA = .05. λ = factor loadings. Fornell-Larcker-Test: AVE = average variance extracted, r^2^_max_ = maximum squared correlation with all other constructs

## Appendix 6

**Table 12:** Mediation analyses (follow-up study 2)

Detailedness	Mediator	DV	a (IV -- > MV)			b (MV -- > DV)			c (IV -- > DV)			c’ (IV -- > DV)			a * b		
			b	t	p	b	t	p	b	t	p	b	t	p	b	LLCI	ULCI
Detailed	P. comprehensiveness	Image	−.894	−4.650	<.001	.377	6.859	<.001	.168	1.083	.280	.505	3.461	.001	−.337	−.535	−.166**
		PI				.400	4.452	<.001	.244	1.032	.303	.602	2.524	.013	−.358	−.590	−.171**
	P. credibility	Image	.102	.631	.529	.609	10.702	<.001	.168	1.083	.280	.106	.880	.880	.062	−.126	.290
		PI				.542	5.184	<.001	.244	1.032	.303	.189	.856	.393	.056	−.116	.266
	P. complexity	Image	−.978	−5.049	<.001	−.260	−4.445	<.001	.168	1.083	.280	−.086	−.545	.587	.254	.098	.457**
		PI				−.272	−2.961	.004	.244	1.032	.303	−.022	−.088	.930	.266	.051	.539*
	P. user friendliness	Image	.597	3.417	.001	.291	4.510	<.001	.168	1.083	.280	−.006	−.040	.968	.174	.057	.336**
		PI				.477	4.871	<.001	.244	1.032	.303	−.040	−.175	.861	.285	.097	.528**
	Presence	Image	.136	.698	.486	.222	3.771	<.001	.168	1.083	.280	.138	.921	.358	.030	−.054	.127
		PI				.284	3.121	.002	.244	1.032	.303	.206	.890	.375	.039	−.070	.171
	Novelty	Image	.214	1.169	.244	.289	4.179	<.001	.168	1.083	.280	.106	.722	.471	.062	−.039	.202
		PI				.308	3.188	.002	.244	1.032	.303	.178	.770	.442	.066	−.042	.217
	Utilitarian benefit	Image	.700	3.362	<.001	.296	5.621	<.001	.168	1.083	.280	−.039	−.268	.789	.207	.067	.407**
		PI				.458	5.963	<.001	.244	1.032	.303	−.076	−.337	.736	.320	.104	.600**
	Hedonic benefit	Image	.744	3.560	<.001	.261	4.874	<.001	.168	1.083	.280	−.260	−.175	.862	.194	.055	.401**
		PI				.357	4.307	<.001	.244	1.032	.303	−.021	−.092	.927	.266	.073	.563**
Nondetailed	P. comprehensiveness	Image	−1.179	−6.065	<.001	.240	4.073	<.001	−.240	−1.620	.107	.044	.277	.728	−.283	−.462	−.137**
		PI				.339	3.650	<.001	−.311	−1.346	.180	.089	.360	.719	−.400	−.715	−.156**
	P. credibility	Image	−.425	−2.410	.017	.486	8.702	<.001	−.240	−1.620	.107	−.033	−.268	.789	−.207	−.381	−.039*
		PI				.599	6.298	<.001	−.311	−1.346	.180	−.056	−.268	.789	−.255	−.492	−.052*
	P. complexity	Image	−.490	−3.312	.001	−.123	−1.159	.131	−.240	−1.620	.107	−.300	−1.966	.051	.060	−.025	.164
		PI				−.223	−1.770	.079	−.311	−1.346	.180	−.420	−1.770	.079	.109	−.024	.290
	P. user friendliness	Image	.052	.303	.763	.239	3.554	.001	−.240	−1.620	.107	−.252	−1.768	.079	.012	−.071	.107
		PI				.453	4.413	<.001	−.311	−1.346	.180	−.335	−1.534	.127	.024	−.135	.194
	Presence	Image	−.308	−1.341	.182	.126	2.449	.015	−.240	−1.620	.107	−.201	−1.371	.172	−.039	−.131	.018
		PI				.297	3.784	<.001	−.311	−1.346	.180	−.220	−.986	.326	−.091	−.250	.046
	Novelty	Image	−.205	−1.133	.259	.136	2.060	.041	−.240	−1.620	.107	−.212	−1.441	.152	−.028	−.104	.022
		PI				.321	3.179	.002	−.311	−1.346	.180	−.245	−1.088	.278	−.066	−.194	.065
	Utilitarian benefit	Image	.356	1.737	.084	.184	3.238	.001	−.240	−1.620	.107	−.305	−2.107	.037	.066	−.009	.177†
		PI				.377	4.355	<.001	−.311	−1.346	.180	−.445	−2.020	.045	.134	−.018	.332†
	Hedonic benefit	Image	.374	1.531	.128	.124	2.564	.011	−.240	−1.620	.107	−.286	−1.954	.053	.046	−.014	.138
		PI				.347	4.818	<.001	−.311	−1.346	.180	−.440	−2.027	.044	.129	−.041	.316

Mediation analyses with PROCESS (model 4, each mediator tested separately), b = unstandardized coefficients; all variables centralize, IV = controllability. Included control variables: gender, age, education, product category knowledge, sustainability attitude. a: regression of the mediator on controllability in a single linear regression analysis; b: regression of DV on the mediator in a multiple regression analysis; c: regression of DV on controllability in a single linear regression analysis; c’: regression of DV on controllability in a multiple regression analysis; a * b: indirect effect; Bootstrapping (*CI-95%, 5000 samples): LLCI: lower limit, ULCI: upper limit. Bootstrapping with CI-90% (†) and CI-99% (**) applied to test for the p < .10 and p < .01-level

## Footnotes

1 In line with marketing literature (e.g., Tangari et al., 2019), we use the term “backfire effect” to describe a mechanism of a marketing measure (here, ARPI controllability) with an intended positive effect, but which, ironically, evokes a negative effect. While we apply the term in the ARPI context, scholars in certain fields use the term “backfire effect” exclusively for a specific effect (i.e., in communications science, Swire-Thompson et al., 2020), which is not meant here.

2 The ARPI app focused on sustainability information for several reasons. Concerns about the ecological and social implications of consumption increased in many industrialized countries over the past years (Mai et al., 2021). Sustainability is a credence attribute and consumers need to rely on information to judge it. According to the low-cost hypothesis, consumers are more prone to act environmentally friendly if additional costs (e.g., search costs) are low (Diekmann & Preisendoerfer, 2003; Reimers & Hoffmann, 2019). Consumers show greater acceptance of products that are clearly identifiable as being socially responsible (Tully & Winer, 2014). A qualitative study by Olsson et al. (2013) revealed that consumers using AR at the point of sale would like to receive further details, particularly about how ecological or ethical the product is. Our AR app accordingly provided sustainability information.

3 A two-way analysis of variance (ANOVA) with the experimental factors shows that ARPI usage time (M_uncontr./nondetailed_ = 63.0 s, M_uncontr./detailed_ = 83.6 s, M_contr./nondetailed_ = 37.7 s, M_contr./detailed_ = 47.4 s) depends on information controllability (F(1, 246) = 25.41, *p* < .001) and information detailedness (F(1, 246) = 6.16, *p* = .014), but there is no interaction effect (F(1, 246) = .80, *p* = .373). All effects remain stable controlling for gender, age, device experience, sustainability attitude, and product category knowledge.

4 We applied measures to ensure the validity of this self-reported variable: Directly after having completed the shopping, the interviewers asked a small test sample to hand in their receipts (n = 8). All self-reported purchases of this test sample were correct.

5 The two groups do not differ with regard to gender (χ^2^(1) = 2.245, *p* = .134), age (t(244) = .131, *p* = .896), device experience (χ^2^(1) = .048, *p* = .827), product category knowledge (t(244) = .722, *p* = .471), and sustainability attitude (t(244) = 1.343, *p* = .181).

6 The booklet study and the ARPI study were run in the same month and in the same hypermarket according to a schedule consisting of weekdays and daytimes to switch treatments. The same interviewers instructed the participants.

7 Excluding participants who used the ARPI for less than three seconds slightly reduced the number of participants in all four cells (before exclusion: N_uncontr./nondetailed_ = 79, N_uncontr./detailed_ = 64, N_contr./nondetailed_ = 60, N_contr./detailed_ = 68; after exclusion: N_uncontr./nondetailed_ = 77, N_uncontr./detailed_ = 61, N_contr./nondetailed_ = 51, N_contr./detailed_ = 61). To ensure that the cut-off of three seconds does not affect the results, we ran robustness checks with cut-offs of two seconds (N = 251) and four seconds (N = 248). All patterns of results reported in Table 3 (for the three seconds cut-off, i.e. interaction controllability × detailedness: β_image_ = −.268, *p* = .022, β_intention_ = −.272, *p* = .021, b_purchase_ = −2.625, *p* = .036) remain stable for the two second cut-off (β_image_ = −.271, *p* = .021, β_intention_ = −.272, *p* = .021, b_purchase_ = −2.605, *p* = .037) and the four second cut-off (β_image_ = −.265, *p* = .023, β_intention_ = −.275, *p* = .019, b_purchase_ = −2.600, *p* = .038).

8 Consumers’ purchase of the focal product depends, arguably, on the type of shopping, i.e. how much they will buy. We, therefore, added the shopping cart’s filling level as a control variable in a robustness check, using a dummy variable distinguishing shoppers with few shopping items (52.4%) from those with many items in the shopping cart (47.6%). Since we do not observe a significant influence of the shopping cart’s filling level, the pattern of effects remains stable (controllability: b = 2.42, Wald(1) = 4.95, *p* = .026; detailedness: b = 2.06, Wald(1) = 3.30, *p* = .069; controllability x detailedness: b = −2.57, Wald(1) = 4.22, *p* = .040).

9 All subjects for the control group were matched from the days on which the ARPI was actually tested in the market. This check confirms the significant ARPI effect (t = 2.88, *p* = .005) outside rush hours, which reflects an increase by 18.7 bottles for other types of beer. We even observed a marginal drop in the number of standard pilsner beer by 10.5 bottles (t = −1.81, *p* = .07). Together, this shift in consumption patterns reflects a difference of 29.2 bottles from other (vs. standard Pilsner) beer types (t = 3.18, *p* = .002). These ARPI effects completely disappeared in shopping rush hours (e.g., overall difference 3.36 bottles, t = .55, *p* = .59), attesting to the robustness of the previous findings. To make sure that our chosen operationalization of rush hour does not distort the results, we reran the analyses with the number of purchasers per 30 min. Again, the matching shows that ARPI substantially increased the shares of other beer types, but only when fewer customers purchased in the market (interaction effect: B = −.83, t = −2.15, *p* = .034). A similar interaction consequently arises for the shift in the consumption of other (vs. standard) beer (B = −1.30, t = −2.39, *p* = .019). More information is provided in Web-Appendix E.
