# Supplementary material for: Augmented reality-delivered product information at the point of sale: when information controllability backfires
Source: J Acad Mark Sci. 2022 Apr 7;50(4):743–76. doi: 10.1007/s11747-022-00855-w (PMC8987517; doi:10.1007/s11747-022-00855-w)
Supplement: Supplementary file 1 — (DOCX 5089 kb) [file 11747_2022_855_MOESM1_ESM.docx]

# Web-Appendix A: Pre-Studies

We ran three pre-studies to answer the following research questions to set the stage for our main research:

1. ***Is the ARPI condition superior to a non-AR condition?***
   (Pre-study 1 and 2 confirmed that the APRI condition is superior.)
2. ***Does ARPI mainly convince consumers via utilitarian or hedonic benefit compared to a non-ARPI condition?***
   (Pre-study 1 and 2 confirmed that mainly the utilitarian benefit affects consumer responses.)
3. ***Can ARPI provide a stronger perceived information-product-fit compared to a QR-code?***
   (Pre-study 3 confirmed that ARPI is superior for creating the information-product-fit.)

## Pre-Study 1

### Objective

The first pre-study tests whether ARPI is superior to a non-ARPI condition. Moreover, it explores the relative importance of utilitarian and hedonic aspects when using ARPI at the point of sale.

### Design

We randomly assigned the participants either to a treatment group with ARPI or a control group without ARPI. The participants watched a customer’s shopping trip in a store. Both groups saw a 25-second video from a first-person perspective. The shopper enters a supermarket, passes by several shelves heading to the cereals section. In the video, the shopper looks at a box of chocolate cereals, takes it from the shelf, and finally inspects the box in order to purchase it. In the experimental condition, the instructions mentioned that the shopper uses AR. The treatment group consequently saw an ARPI overlay appear at the moment the shopper inspects the cereal box. The ARPI has the same design and displayed the same information as in the main study.

The participants answered a brief questionnaire measuring the hedonic and utilitarian benefits of the shopping on four five-point items taken from Voss et al. (2003) (hedonic: “The shopping was …,” “fun,” “exciting,” “delightful,” and “thrilling,” M = 3.15, SD = .85, α = .79; utilitarian: “effective,” “functional,” “necessary,” “practical,” M = 2.75, SD = 1.02, α = .92). We measured purchase intention for the product with three seven-point items (“definitely intend to buy,” “high purchase interest”, “probably will buy it”, M = 3.52, SD = 1.53, α = .94). Exploratory factor analysis yielded three factors, which supports discriminant validity. In all, 469 students (52% male, 21.9 years, SD = 2.76) participated in the online experiment at two universities (n_1_ = 340, n_2_ = 129) for study credits.

### Results

The ARPI group evaluated the shopping experience generally better than the control group (hedonic: M_ARPI_ = 3.19, M_control_ = 2.26, t(467) = 11.134, *p* < .001, Cohen’s d = 1.030; utilitarian: M_ARPI_ = 3.32, M_control_ = 2.95, t(467) = 4.796, *p* < .001, d = .444) and purchase intentions were higher (M_ARPI_ = 3.94, M_control_ = 3.05, t(467) = 6.525, *p* < .001, d = .604). More importantly, regression analysis shows that, in the ARPI group, purchase intentions primarily based on utilitarian motives (β_utilitarian_ = .302, t = 4.354, *p* < .001, β_hedonic_ = .093, t = 1.341, *p* = .181, R² = .13), whereas it mainly based on hedonic motives in the control group (β_hedonic_ = .260, t = 3.805, *p* < .001; β_utilitarian_ = .128, t = 1.872, *p* = .063, R² = .11). This remains stable when controlling for age and gender. As a further robustness check, we also ensured that the pattern occurred for both subsamples of the two universities.

### Discussion

The study confirms that ARPI improves hedonic and utilitarian benefits during shopping, but particularly the utilitarian aspects guide purchase decisions. This effect is clearly AR-specific and does not occur in the non-AR condition.

## Pre-Study 2

### Objective

The objective of the second pre-study is to contrast the ARPI against a control group without ARPI. Additionally, the study asks whether consumers react differently to ARPI provided via tablets or glasses, and we added a group that is exposed to a QR code. Moreover, the study tests again whether the utilitarian and hedonic benefits play different roles in the ARPI conditions and the control group.

### Design

We ran a between-subjects online experiment with three media conditions (QR, ARPI tablet, ARPI glasses) and a control group without additional information. We created videos for all four conditions, which show a shopping scene from a first-person perspective. Participants were asked to imagine that they were this person. Participants in the three experimental groups were further instructed to imagine they are using a (1) tablet with a QR scanner (QR condition), (2) a tablet with an AR function (AR tablet condition), or AR glasses (AR glasses condition), which allow them to see additional product information as soon as the tablet’s or the glasses’ camera fixates a product. In all four conditions, the video started by showing a shelf in a supermarket containing several packages of cereal bars. The person takes one box of chocolate cereal bars out of the shelf. The short video ended with the person holding the box in the hand.

**Figure Web-A1. Experimental Treatment (Screenshots of the Videos)**

|  | **Screenshot 1** | **Screenshot 2 (final screen)** |
| --- | --- | --- |
| **Control group** | **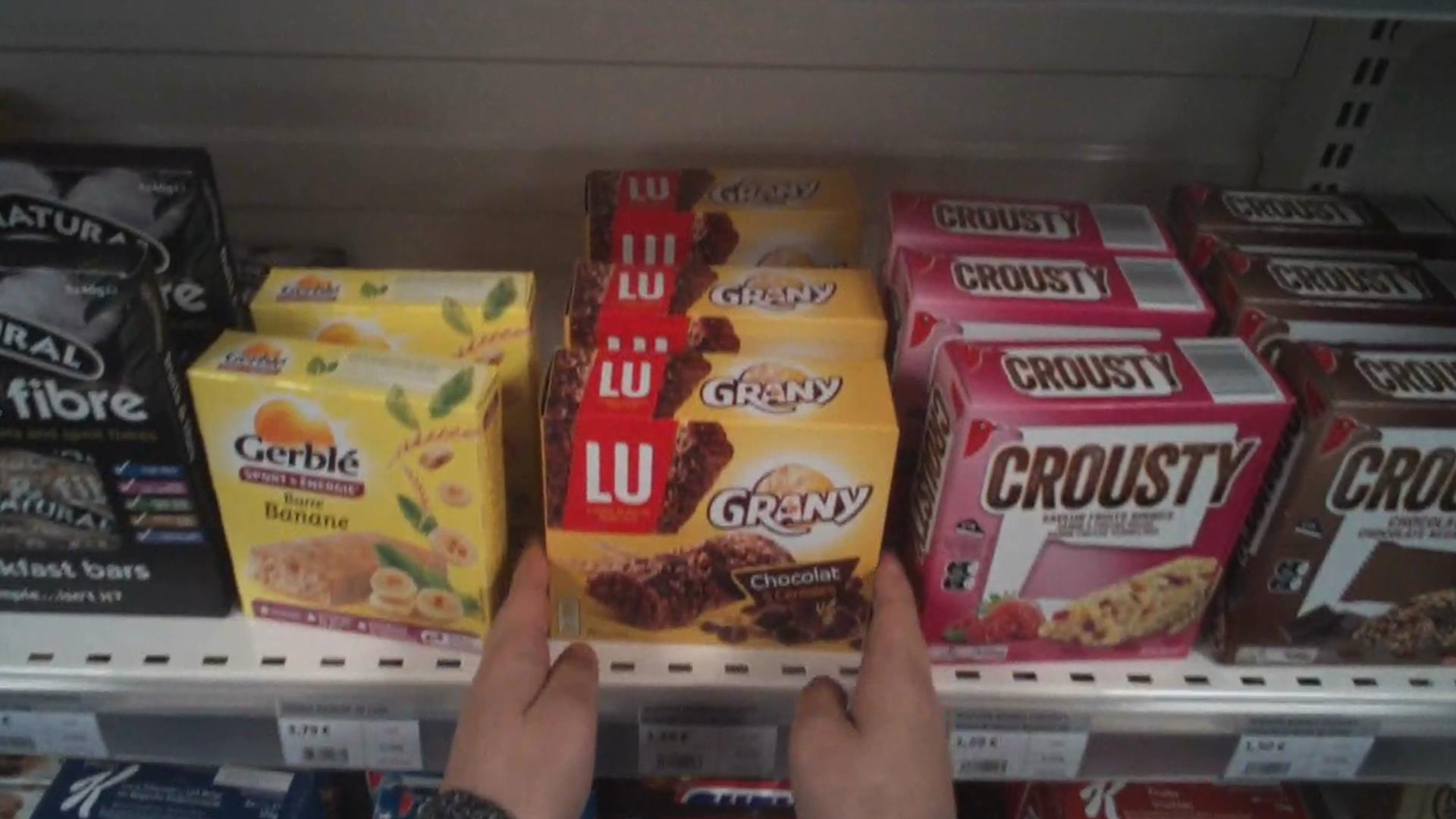** | **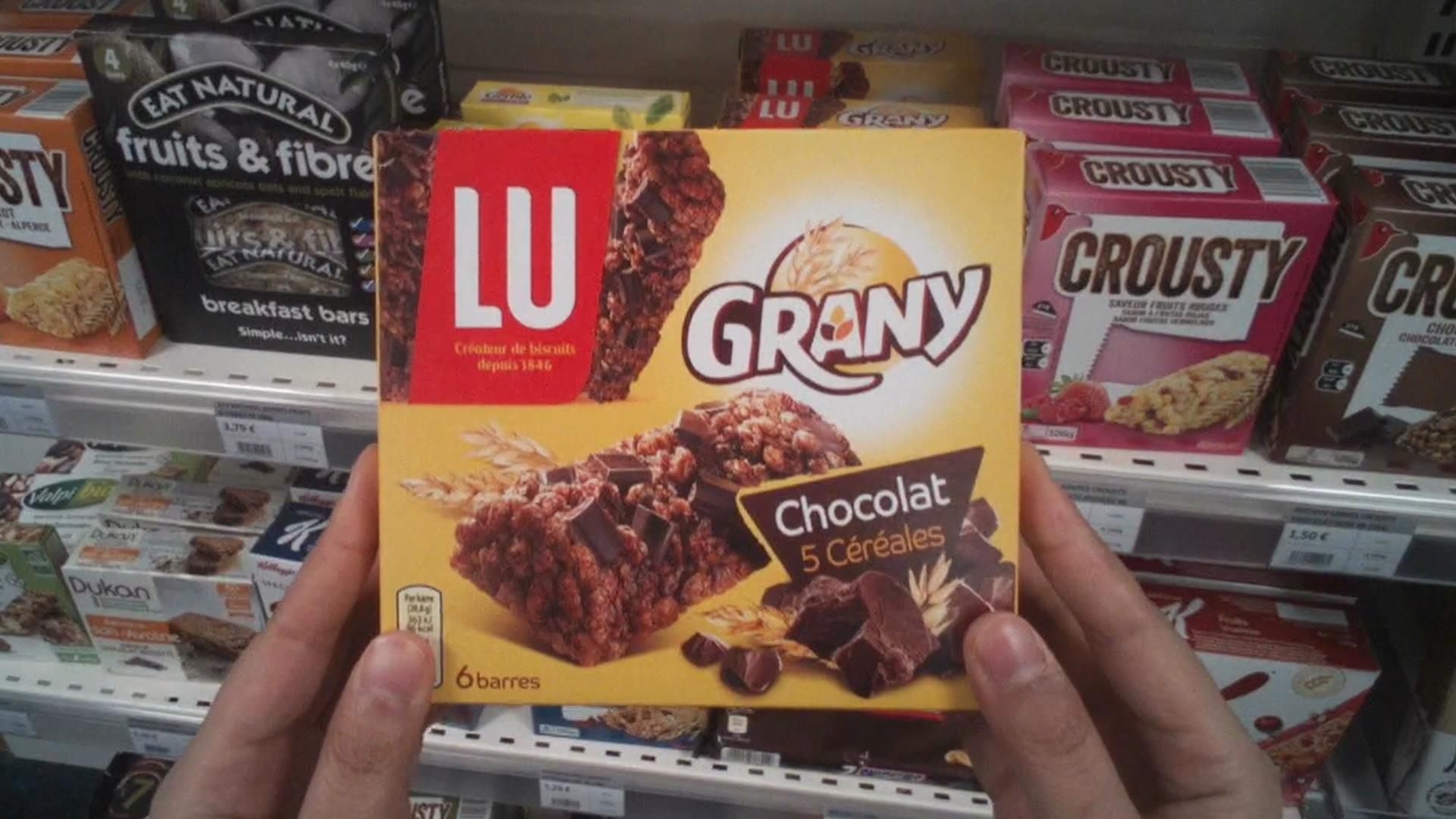** |
| **QR** | **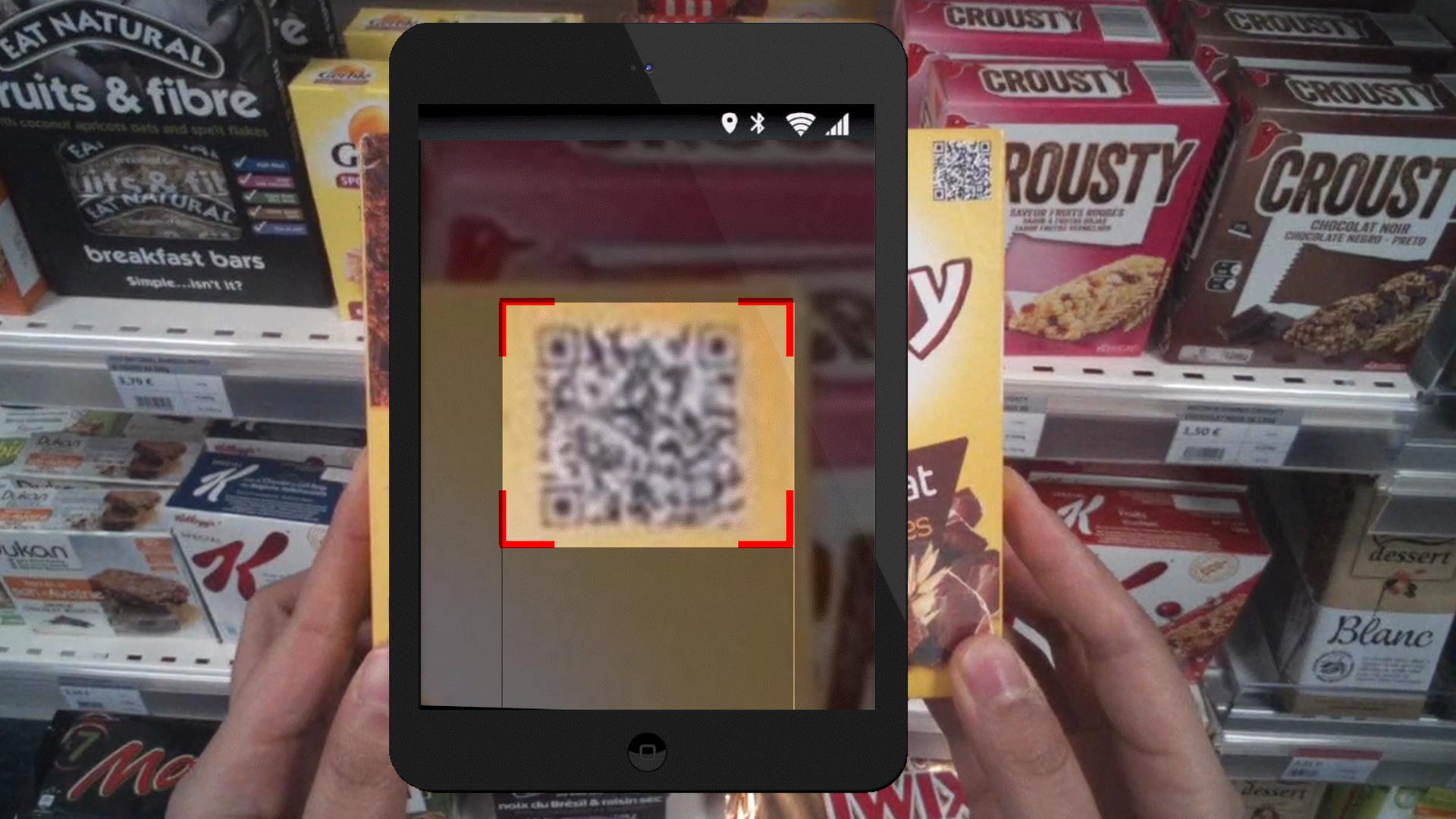** | **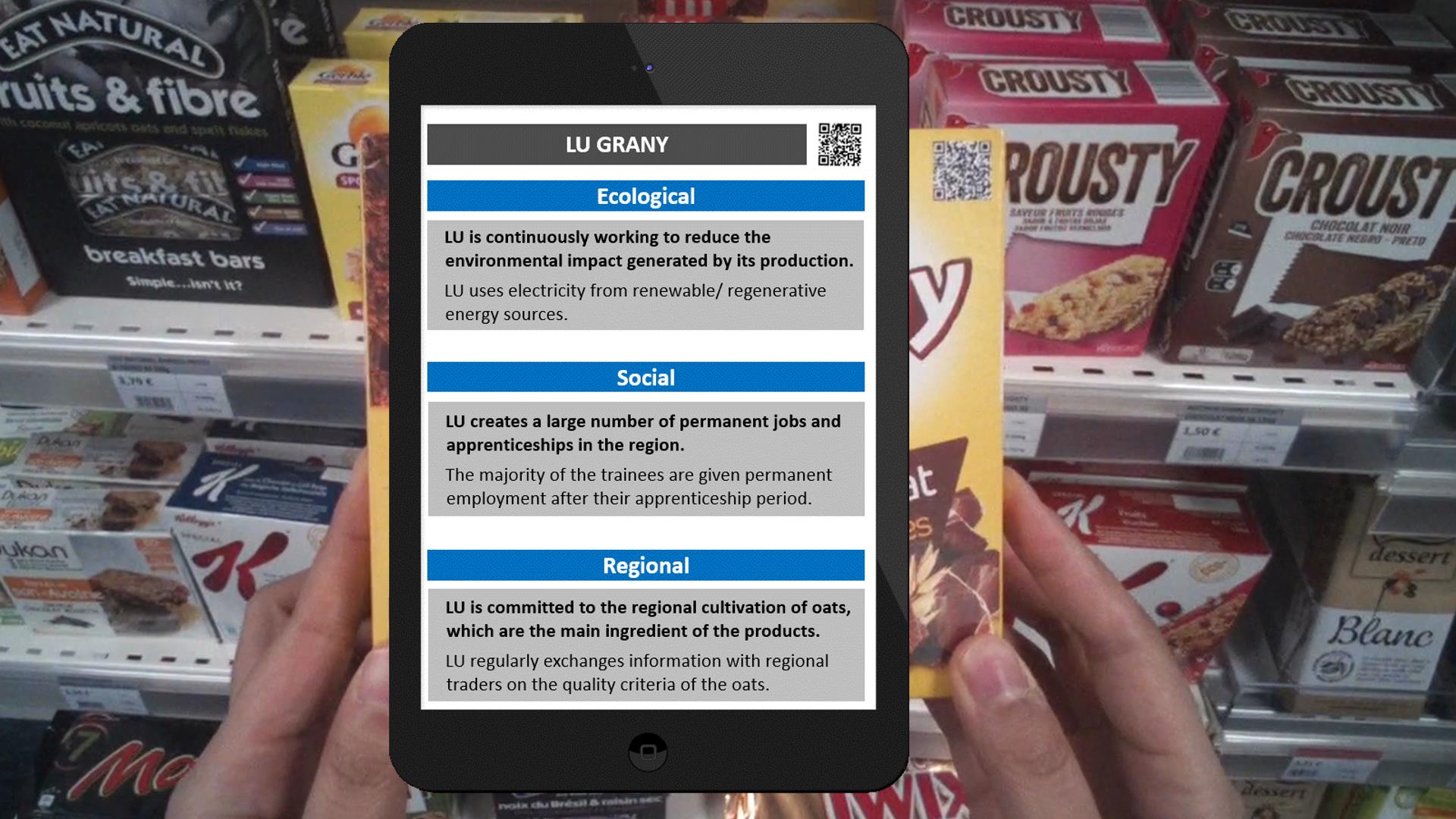** |
| **AR tablet** | **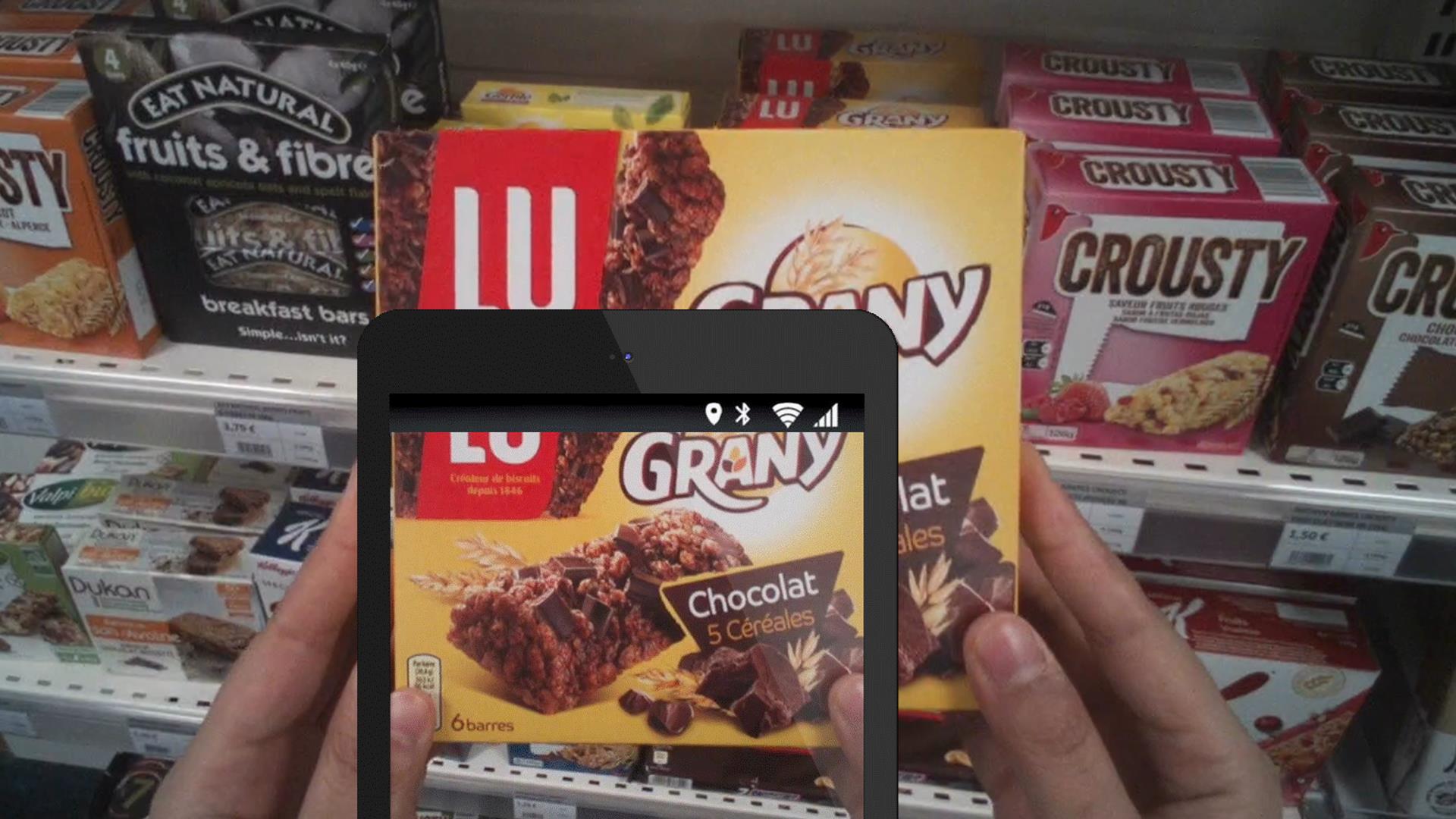** | **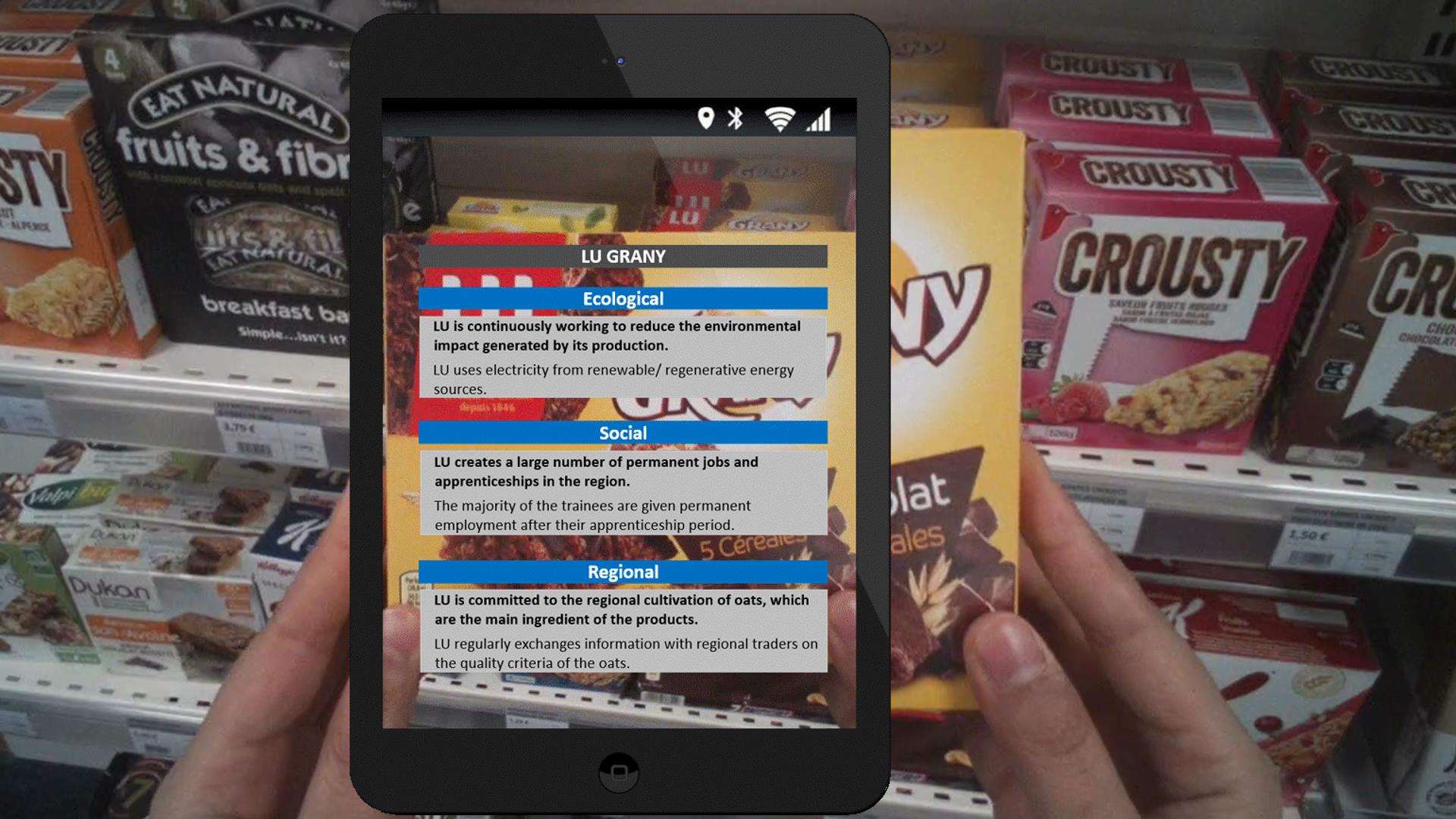** |
| **AR glasses** | **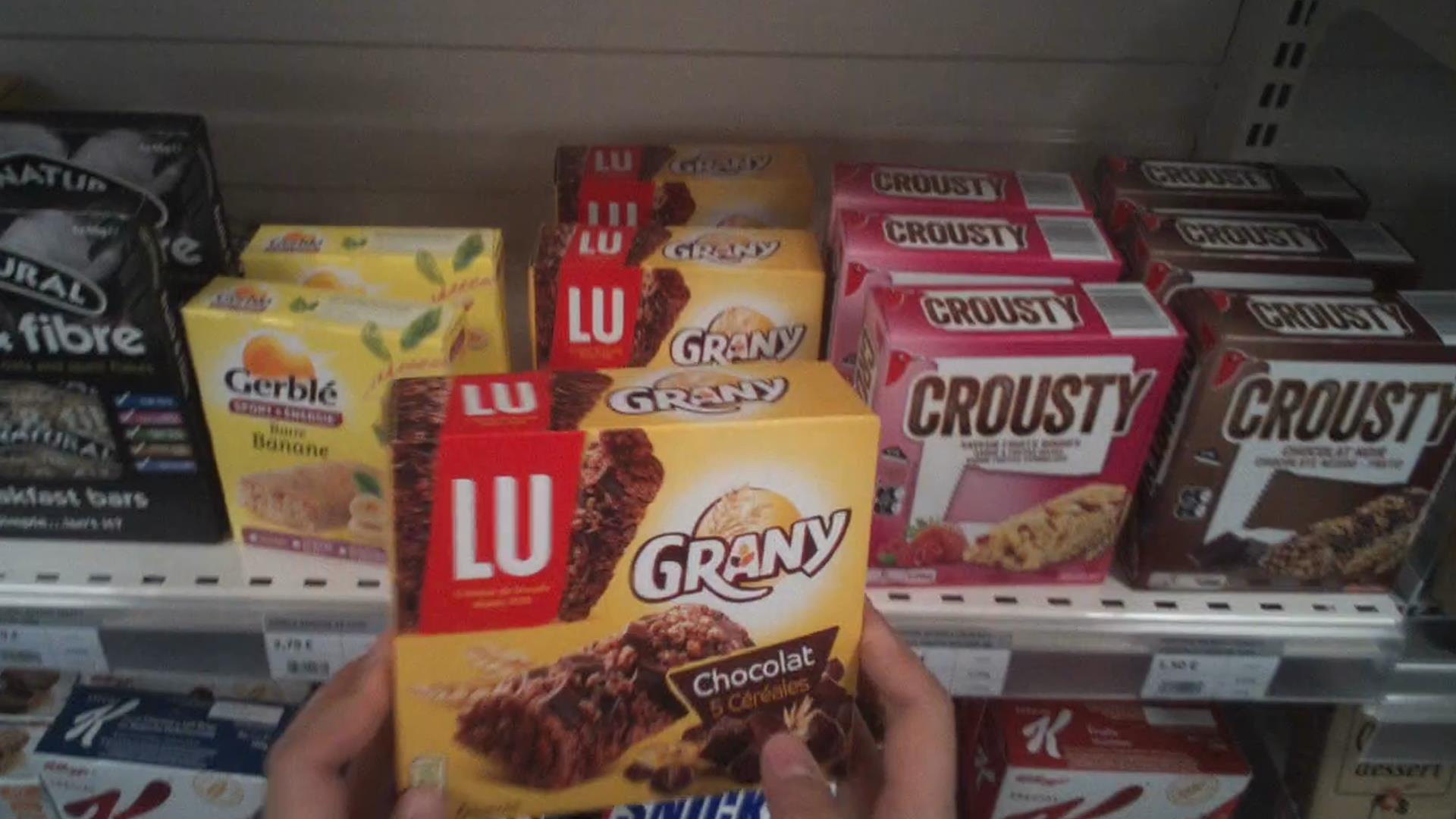** | **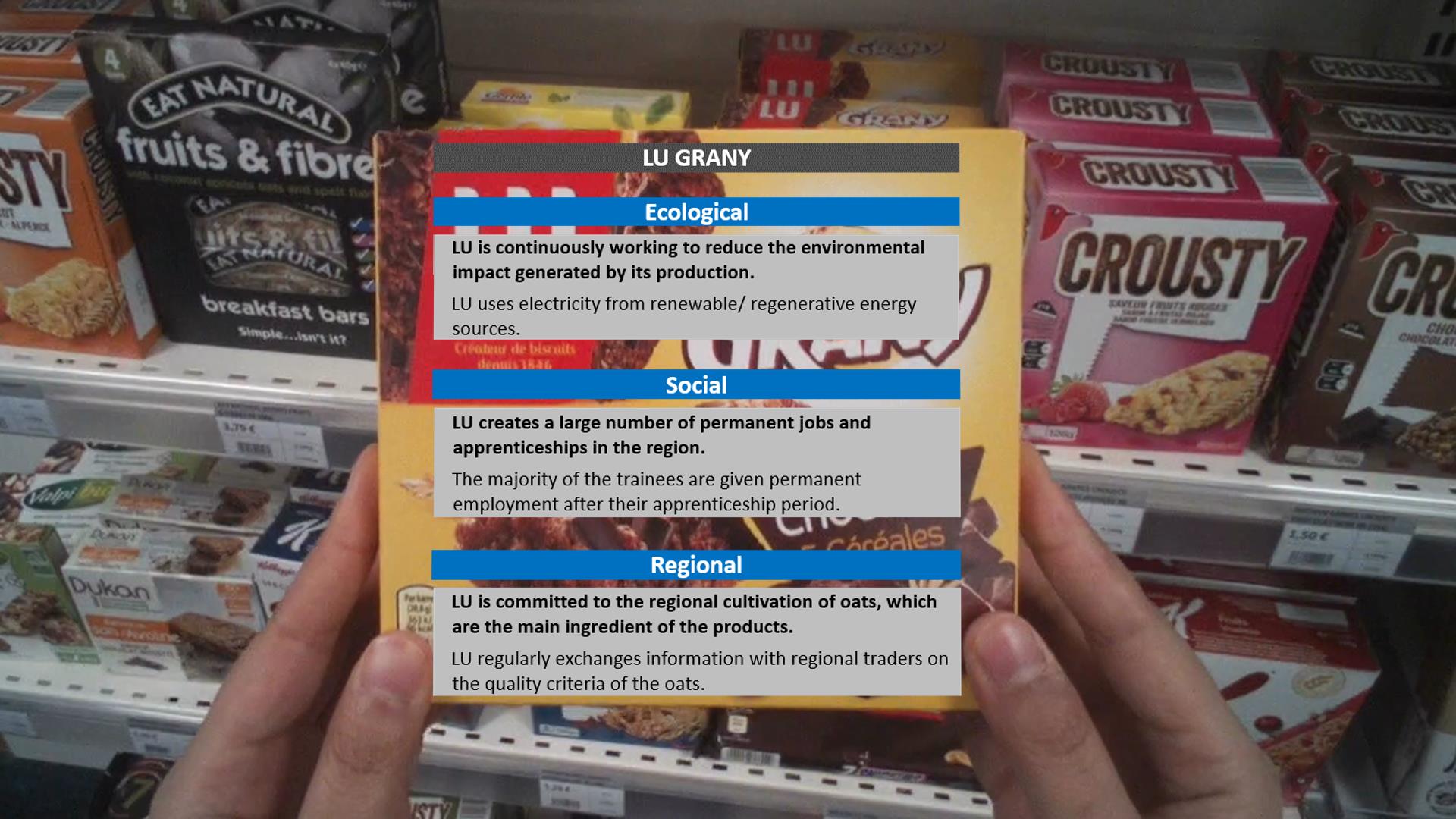** |

The second part of the video depends on the experimental treatment. In all three experimental conditions, additional information about the product’s sustainability appeared, while there was no additional information in the control group. The information consisted of the three headings ecological, social, and regional (white on blue background). Under each heading, there was a respective statement (bold, black) and a supportive argument (black) on a grey background. All statements were also used in the main study, and they were shown identically in all three experimental conditions. However, the way how the information appeared is different across the groups. Participants in the QR condition saw a tablet with the camera moving over the cereal box. The tablet’s camera fixated the QR code on the upper right corner of the cereal bar box. Additional information about the product’s sustainability appeared on the screen of the tablet in front of a plain white background. In the ARPI tablet condition, there was also a tablet with the camera moving over the box. The tablet showed a part of the scene, including a part of the shelf with other boxes in the background and the focal product in the foreground held in the consumer’s hands. After the ARPI app had fixated the box, additional information appeared hovering over the picture of the box. This video visualized the procedure that participants completed in the main study. In the ARPI glasses condition, the information was directly placed on the cereal box simulating the perspective of a consumer wearing AR glasses, which display the environment, additionally integrating digital content. Screenshots of the videos are given in Figure Web-A1.

We measured brand image with three items (M = 4.43, SD = 1.02, Cronbach’s α = .84) and purchase intention with three items (M = 3.60, SD = 1.41, α = .92) on seven-point scales. We added a three-item scale for utilitarian benefits (M = 4.19, SD = 1.53, α = .83), and a three-item scale for hedonic benefit (M = 3.73, SD = 1.57, α = .88), both taken from Voss et al. (2003). As controls, we measured the self-reported product category knowledge in the food domain with two items (M = 4.89, SD = 1.37, α = .85, ρ = .85) and sustainability attitude with two items (M = 5.06, SD = 1.22, α = .87, ρ = .87). All scales were measured on seven-point scales. Confirmatory factor analysis with all multi-item constructs shows a good model fit (χ^2^_(89)_ =165.00, χ^2^/d.f. = 1.85; CFI = .98; RMSEA = .05). The analysis confirms discriminant validity, because each construct's average variance extracted exceeds the maximum of the squared correlations with all latent variables (Fornell and Larcker 1981).

We gathered data of 308 consumers via snowballing through social media. Subjects were randomly assigned to one of the three experimental groups or the control group. The age of the participants ranged from 13 to 71 years, with a mean of 28.1 years (SD = 11.5); 44.2% of the participants are men, 55.2% female, and 0.6% diverse. Of the participants, 52.1% are doing their studies or have a university degree. The subjects were randomly assigned to the group with the QR-code (68), the ARPI tablet (79), the ARPI glasses (75), and the control group (86).

### Results

The means of brand image and purchase intention for all four groups are displayed in Figure Web-A2. The means of the brand image differ across the four treatments: M_control group_ = 4.1, M_QR_ = 4.4, M_ARPI-tablet_ = 4.5, and M_ARPI-glasses_ = 4.8. OLS-regression with dummy variables for the treatment (control group as baseline) confirm that the differences between the experimental groups and the control group are statistically significant (QR-Code: β = .146, t = 2.223, *p* = .027; ARPI-tablet: β = .181, t = 2.733, *p* = .007; ARPI-glasses: β = .290, t = 4.377, *p* < .001). The means of the purchase intention also differ: M_control group_ = 3.4, M_QR_ = 3.6, M_ARPI-tablet_ = 3.5, and M_ARPI-glasses_ = 3.9. OLS-regression with dummy variables for the treatment (control group as baseline) confirm that the difference between the experimental groups and the control group is statistically significant for the ARPI-glasses treatment (ARPI-glasses: β = .172, t = 2.550, *p* = .011), while there is no effect for the QR-Code (β = .059, t = .884, *p* = .377) and the ARPI-tablet (β = .044, t = .652, *p* = .515). These differences remain stable when adding the control variables (sex, age, product category involvement, sustainability attitude) to the analysis (see Table Web-A1).

**Figure Web-A2. Comparison of Media (Pre-Study 2)**

**Table Web-A1. Effect of Treatment (Pre-Study 2)**

|  | **DV: Brand  Image** | | |  | **DV: Purchase  Intention** | | |  | **DV: Utilitarian  Benefit** | | |  | **DV: Hedonic  Benefit** | | |
| --- | --- | --- | --- | --- | --- | --- | --- | --- | --- | --- | --- | --- | --- | --- | --- |
|  | β | t | p |  | β | t | p |  | β | t | p |  | β | t | p |
| Sex^1^ | .028 | .492 | .623 |  | .105 | 1.825 | .069 |  | .015 | .257 | .789 |  | -.009 | -.164 | .870 |
| Age | -.181 | -3.208 | <.001 |  | -.092 | -1.591 | .113 |  | -.196 | -3.515 | <.001 |  | -.240 | -4.365 | <.001 |
| Product category knowledge | .034 | .545 | .587 |  | -.075 | -1.157 | .248 |  | .087 | 1.401 | .162 |  | .076 | 1.245 | .214 |
| Sustainability Attitude | -.035 | -.555 | .579 |  | -.019 | -.291 | .771 |  | .086 | 1.363 | .174 |  | .084 | 1.361 | .174 |
|  |  |  |  |  |  |  |  |  |  |  |  |  |  |  |  |
| *Treatment (baseline: CG)²* |  |  |  |  |  |  |  |  |  |  |  |  |  |  |  |
| - QR-Code | .156 | 2.382 | .018 |  | .060 | .890 | .374 |  | .234 | 3.625 | <.001 |  | .225 | 3.538 | <.001 |
| - ARPI tablet | .192 | 2.914 | .004 |  | .041 | .601 | .548 |  | .322 | 4.967 | <.001 |  | .290 | 4.524 | <.001 |
| - ARPI glasses | .292 | 4.437 | <.001 |  | .158 | 2.340 | .020 |  | .254 | 3.919 | <.001 |  | .326 | 5.086 | <.001 |
|  |  |  |  |  |  |  |  |  |  |  |  |  |  |  |  |
| F | 4.559 |  |  |  | 2.239 |  |  |  | 6.083 |  |  |  | 7.276 |  |  |
| R² | .096 |  |  |  | .050 |  |  |  | .126 |  |  |  | .146 |  |  |
| R²_adj_ | .075 |  |  |  | .028 |  |  |  | .104 |  |  |  | .126 |  |  |

Notes: OLS regression, β = standardized coefficients. ^1^0: male, 1: female and divers, ^2^treatment dummy-coded, control group without treatment as the baseline.

To better understand the differences between the treatments, we ran several regression analyses contrasting two groups (including the control variables). Replicating the results given above, the analyses found statistically significant differences on most dependent variables for the contrast of the ARPI tablet vs. the control group (brand image: β = .22, t = 2.94, *p* = .004; purchase intention: β = .04, t = .56, *p* = .576; utilitarian benefit: β = .35, t = 4.84, *p* < .001; hedonic benefit: β = .34, t = 4.68, *p* < .001), the contrast of ARPI glasses vs. the control group (brand image: β = .35, t = 4,65, *p* < .001; purchase intention: β = .19, t = 2.43, *p* = .016; utilitarian benefit: β = .32, t = 4.27, *p* < .001; hedonic benefit: β = .4, t = 5.56, *p* < .001), and the contrast of the QR code and the control group (brand image: β = .19, t = 2.38, *p* = .019; purchase intention: β = .07, t = .87, *p* = .385; utilitarian benefit: β = .28, t = 3.53, *p* < .001; hedonic benefit: β = .28, t = 3.49, *p* < .001). There are no differences for the contrast of ARPI glasses and ARPI tablet (brand image: β = .12, t = 1.50, *p* = .135; purchase intention: β = .13, t = 1.62, *p* = .107; utilitarian benefit: β = -.07, t = -.91, *p* = .365; hedonic benefit: β = .05, t = .60, *p* = .553), the contrast of ARPI tablet and the QR code (brand image: β = .03, t = .39, *p* = .699; purchase intention: β = -.02, t = -.28, *p* = .777; utilitarian benefit: β = .07, t = .91, *p* = .367; hedonic benefit: β = .07, t = .85, *p* = .395), and the contrast of the ARPI glasses and the QR code (brand image: β = .16, t = 1.93, *p* = .055; purchase intention: β = .13, t = 1.49, *p* = .138; utilitarian benefit: β = .01, t = .17, *p* = .867; hedonic benefit: β = .11, t = 1.29, *p* = .198).

Next, we ran a separate OLS regression analysis for all four groups with brand image and purchase intention as dependent variables and the utilitarian and hedonic benefits as predictors. As visualized in Table Web-A2, the utilitarian benefit is particularly relevant in the three experimental groups, while the hedonic benefit is more important for the control group.

**Table Web-A2: Different Effects of Utilitarian and Hedonic Benefit (Pre-Study 2)**

| **DV** | **IV** | **CG** | | |  | **QR** | | |  | **ARPI tablet** | | |  | **ARPI glasses** | | |
| --- | --- | --- | --- | --- | --- | --- | --- | --- | --- | --- | --- | --- | --- | --- | --- | --- |
|  |  | β | t | p |  | β | t | p |  | β | t | p |  | β | t | p |
| Brand image | Utilitarian | .143 | 1.113 | .269 |  | **.549** | **4.018** | **<.001** |  | **.365** | **2.765** | **.007** |  | **.446** | **3.489** | **<.001** |
|  | Hedonic | .235 | 1.842 | .069 |  | .043 | .313 | **.755** |  | .235 | 1.781 | .079 |  | .091 | .714 | .478 |
|  |  |  |  |  |  |  |  |  |  |  |  |  |  |  |  |  |
| Purchase intention | Utilitarian | .200 | 1.642 | .104 |  | **.489** | **3.585** | **<.001** |  | **.442** | **3.512** | **<.001** |  | **.293** | **2.164** | **.034** |
|  | Hedonic | **.304** | **2.501** | **.014** |  | .125 | .919 | .362 |  | .209 | 1.661 | .101 |  | .155 | 1.145 | .256 |

Notes: OLS regression, β = standardized coefficients.

### Discussion

The study confirms the positive effects of the ARPI conditions on brand image and purchase intention. Moreover, in contrast to the group without ARPI, the effects on brand image and purchase intention are mainly driven by the utilitarian (rather than hedonic) benefits for those consumers who have been exposed to ARPI. Remarkably, there are no differences across the three ways of providing additional digital information. However, in the present study, the videos only showed the landing page of the digital device, which is quite similar for ARPI and QR codes. To gain a deeper understanding of the subsequent more vivid interaction with the device, we will explore the difference between ARPI and QR codes in more detail in the next pre-study, with a video that also shows the information controllability.

## Pre-Study 3

### Objective

The objective of the third pre-study is to test whether ARPI creates a stronger perceived information-product-fit than the QR-condition. In contrast to the second pre-study, the video in this pre-study shows the ARPI in a more realistic, vivid 3D-manner, and it shows how users interact with the digital information to demonstrate the information controllability.

### Design

We ran a between-subjects online experiment with the two media conditions, QR code and ARPI. We created videos from a first-person perspective that show how a user scans a product and selects information with a tablet computer. Participants were asked to imagine that they were this person. As a product example, we used a package of chocolate cereals. In the ARPI condition, the respondents first saw how they can scan a product. The additional product information appeared on the screen of the tablet hovering over the product package. To create a more vivid impression, the video showed how the user could move around with the tablet and that the information seemed to stick on the product package in a 3D manner. Next, the video showed how participants could select deeper information by virtually clicking on a specific piece of information on the product package on the tablet screen. In the QR condition, it was first shown how the user scans a QR-code on the package, and afterward the additional information appeared on the tablet screen with a white background. It was then shown how to select deeper information by clicking on a specific piece of information. Screenshots of the videos are given in Figure Web-A3.

**Figure Web-A3. Experimental Treatment (Screenshots of the Videos)**

|  | **Screenshot 1** | **Screenshot 2** |
| --- | --- | --- |
| **QR** | **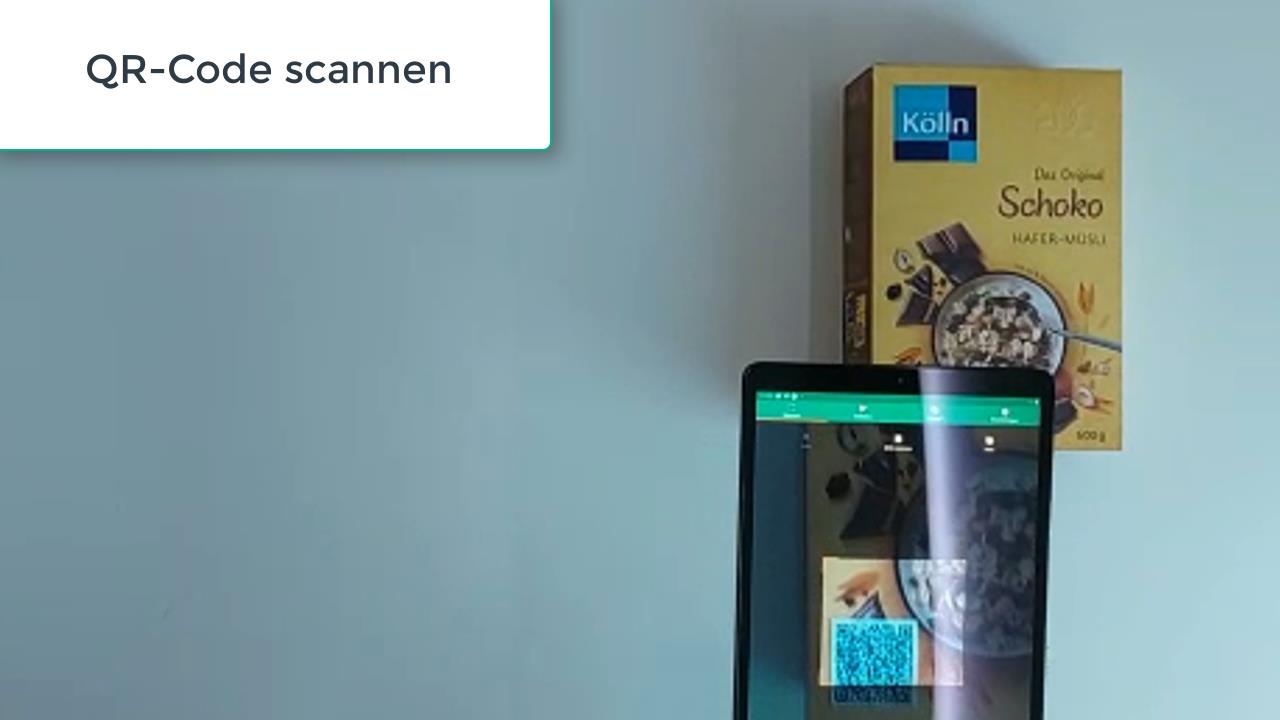** | **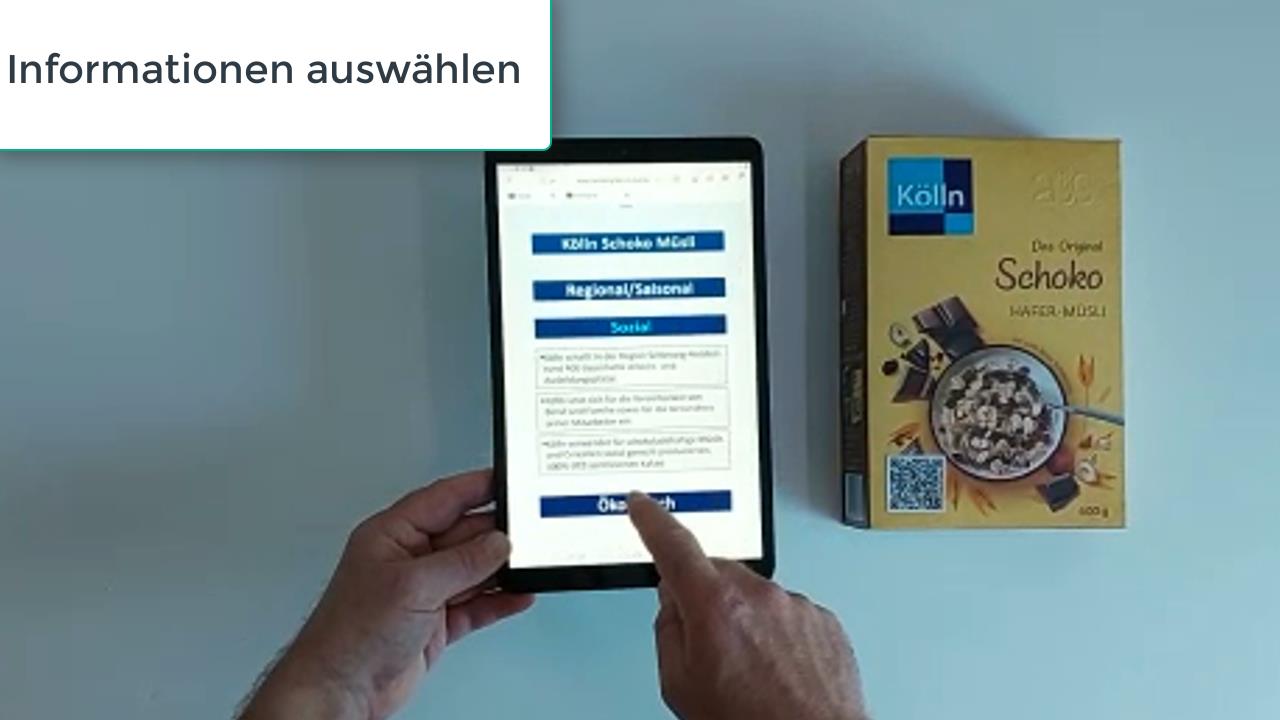** |
| **AR tablet** | **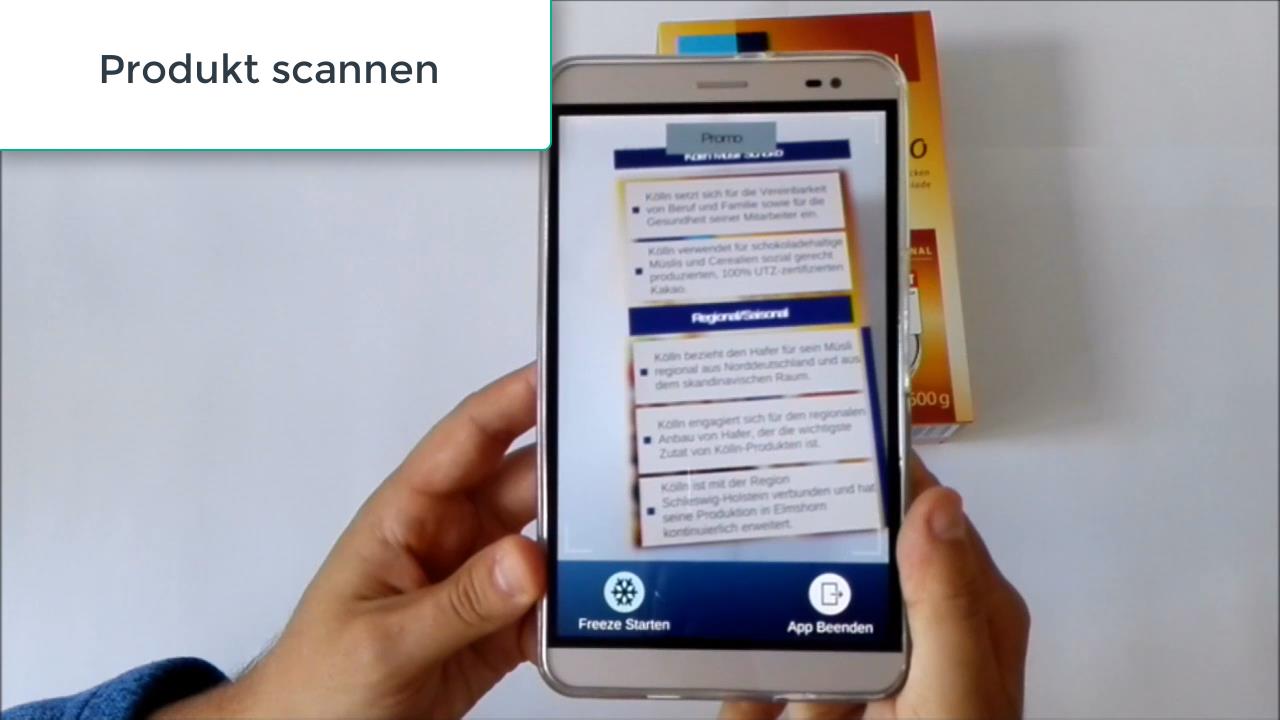** | **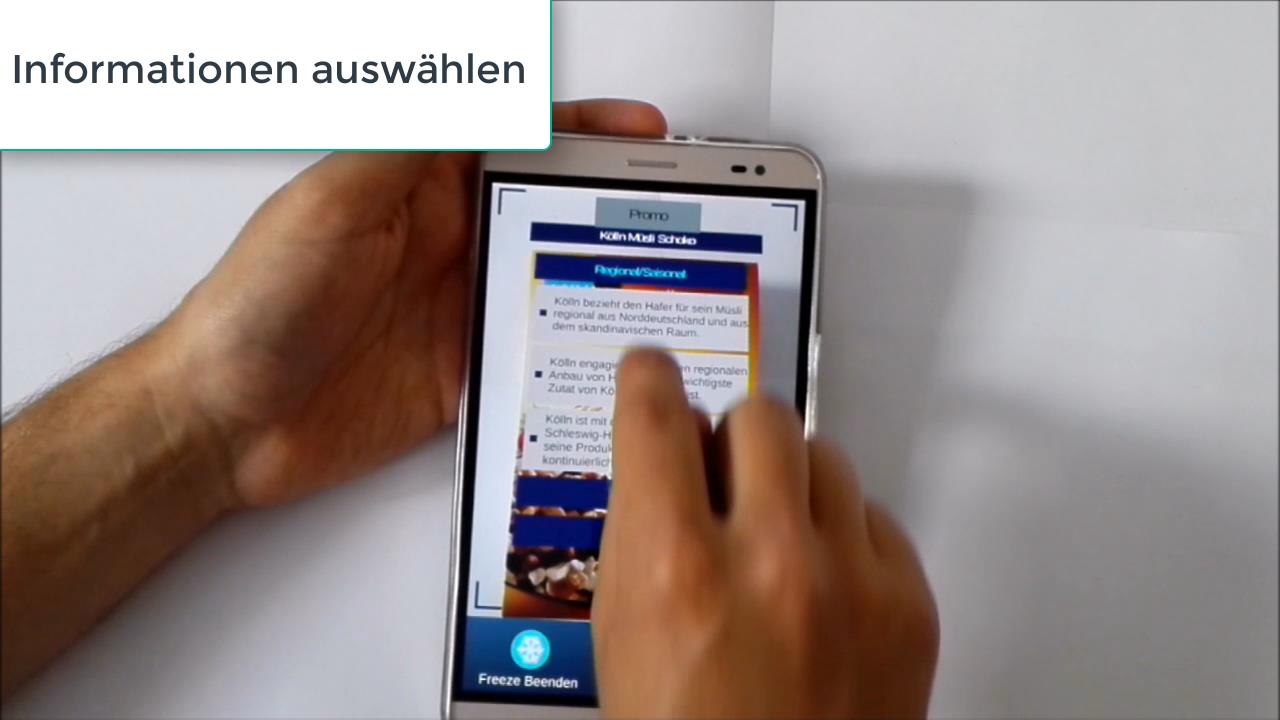** |

We created a new scale on information-product-fit with five items (M = 5.16, SD = 1.04, α = .87, “The provided information…”, “… was well linked to the product”, “… fits well to the product”, “… created a unit with the product”, “… was part of the product”, “… strongly defined the product”). Like in pre-study 2, we again measured brand image with three items (M = 5.16, SD = 1.09, Cronbach’s α = .90) and purchase intention with three items (M = 4.08, SD = 1.63, α = .96). We added a three-item scale for utilitarian benefits (M = 4.78, SD = 1.43, α = .82), and a three-item scale for hedonic benefit (M = 4.42, SD = 1.45, α = .88), both taken from Voss et al. (2003). To account for the more vivid nature of the ARPI vs. the QR-code, we added a three-item-scale of presence adapted from Huang and Liao (2015) (M = 4.28, SD = 1.31, α = .83). As controls, we again measured the self-reported product category knowledge in the food domain with two items (M = 4.62, SD = 1.20, ρ = .80) and sustainability attitude with two items (M = 5.14, SD = 1.09, ρ = .84). All scales were measured on seven-point scales. Confirmatory factor analysis with all multi-item constructs shows a good model fit (χ^2^_(224)_ =404.48, χ^2^/d.f. = 1.81; CFI = .96; RMSEA = .06). The analysis confirms discriminant validity because each construct's average variance extracted exceeds the maximum of the squared correlations with all latent variables (Fornell and Larcker 1981).

We gathered data of 261 consumers via Prolific (German consumers), having to exclude one because the person did not agree with the data protection disclaimer. Subjects were randomly assigned to one of the two groups, QR code (121) vs. ARPI (139). The age of the participants ranged from 18 to 65 years, with a mean of 28.0 years (SD = 9.0); 45.4% of the participants are men, 53.1% female, and 1.5% divers. For this sample, 60.0% are doing their studies or have a university degree.

### Results

A t-test (two-tailed) confirms that consumers perceive a higher information-product-fit in the ARPI condition compared to the QR condition (M_QR_ = 5.02, M_AR_ = 5.28, t(258) = -2.04, *p* = .042; Cohen’s d = -.254). Moreover, the ARPI outperforms the QR code with respect to presence (M_QR_ = 4.08, M_AR_ = 4.46, t(258) = -2.35, *p* = .020, d = -.292) and the perceived hedonic benefit (M_QR_ = 4.21, M_AR_ = 4.61, t(258) = -2.25, *p* = .025, d = -.280). The means for these variables are presented in Figure Web-A4. There are no significant differences between the QR code and the ARPI for utilitarian benefit (M_QR_ = 4.89, M_AR_ = 4.68, t(258) = 1.21, *p* = .229, d = .150), brand image (M_QR_ = 5.20, M_AR_ = 5.12, t(258) = .62, *p* = .538, d = .077), and purchase intention (M_QR_ = 4.19, M_AR_ = 3.99, t(258) = .98, *p* = .331, d = .121). All results remain stable when running the analysis as an ANCOVA with the control variables age, sex, education, product category knowledge, and sustainability attitude.

**Figure Web-A4. Mean Comparisons (Pre-Study 3)**

### Discussion

This pre-study aimed to test whether ARPI creates a stronger perceived information-product-fit than a QR code. With the help of a video that visualizes the ARPI in realistic, vivid 3D-manner and which also demonstrates how users interact with the digital information, the experimental study actually confirms the superior effect of the ARPI with regard to the perceived information-product-fit. ARPI also evokes a higher degree of presence and a stronger hedonic benefit than the QR condition.

# Web-Appendix B: Further Information about the Main Study

**Figure Web-B1: Map of the Hypermarket**

**Table Web-B1. AR-Controllability Effect on Purchase Depending on Medium (Main Study)**

| *Purchase of the product promoted in the ARPI* | **ARPI** | | | | |  | **Paper Booklet** | | | | |  | **Whole Sample** | | | | |
| --- | --- | --- | --- | --- | --- | --- | --- | --- | --- | --- | --- | --- | --- | --- | --- | --- | --- |
|  | b | SE | Wald | p | odds exp(b) |  | b | SE | Wald | p | odds exp(b) |  | b | SE | Wald | p | odds exp(b) |
| Constant | -6.878 | 1.886 | 13.302 | <.001 | .001 |  | -1.542 | 1.848 | 1.272 | .397 | 3.836 |  | -5.952 | 1.526 | 15.217 | <.001 | .003 |
| *Controls* |  |  |  |  |  |  |  |  |  |  |  |  |  |  |  |  |  |
| Gender^1^ | -.677 | .531 | 1.626 | .202 | .508 |  | -.132 | .576 | .053 | .818 | .876 |  | -.465 | .378 | 1.509 | .219 | .628 |
| Age | .019 | .016 | 1.420 | .233 | 1.019 |  | .039 | .017 | 4.996 | .025 | 1.039 |  | .026 | .011 | 5.274 | .022 | 1.026 |
| Device experience^2^ | .152 | .472 | .104 | .747 | 1.165 |  | -1.117 | .631 | 3.135 | .077 | .327 |  | -.264 | .359 | .543 | .461 | .768 |
| Product category knowledge | -.009 | .247 | .001 | .970 | .991 |  | .039 | .400 | .010 | .921 | 1.040 |  | -.008 | .208 | .002 | .968 | .992 |
| Sustainability attitude | .332 | .208 | 2.546 | .111 | 1.394 |  | -.133 | .252 | .279 | .598 | .876 |  | .199 | .155 | 1.646 | .200 | 1.221 |
| *Treatment* |  |  |  |  |  |  |  |  |  |  |  |  |  |  |  |  |  |
| **Controllability** | **2.495** | **1.082** | **5.322** | **.021** | **12.124** |  | -1.123 | .809 | 1.927 | .165 | .325 |  | **2.409** | **1.076** | **5.013** | **.025** | **11.122** |
| **Detailedness** | 2.123 | 1.132 | 3.514 | .061 | 8.356 |  | -1.150 | .771 | 2.220 | .136 | .317 |  | 2.085 | 1.124 | 3.440 | .064 | 8.047 |
| **Controllability ×   detailedness** | **-2.625** | **1.250** | **4.410** | **.036** | **.072** |  | 1.345 | 1.192 | 1.272 | .259 | 3.836 |  | **-2.487** | **1.238** | **4.035** | **.045** | **.083** |
| *Medium* |  |  |  |  |  |  |  |  |  |  |  |  |  |  |  |  |  |
| **Medium** |  |  |  |  |  |  |  |  |  |  |  |  | **2.502** | **1.124** | **4.951** | **.026** | **12.205** |
| **× controllability** |  |  |  |  |  |  |  |  |  |  |  |  | **-3.488** | **1.326** | **6.917** | **.009** | **.031** |
| **× detailedness** |  |  |  |  |  |  |  |  |  |  |  |  | **-3.050** | **1.344** | **5.152** | **.023** | **21.118** |
| **× controllability ×  detailedness** |  |  |  |  |  |  |  |  |  |  |  |  | **3.552** | **1.666** | **4.543** | **.033** | **34.879** |
| –2 log-likelihood | 139.256 |  |  |  |  |  | 90.815 |  |  |  |  |  | 234.921 |  |  |  |  |
| Cox & Snell R² | .079 |  |  |  |  |  | .092 |  |  |  |  |  | .073 |  |  |  |  |
| Nagelkerke’s R² | .159 |  |  |  |  |  | .163 |  |  |  |  |  | .140 |  |  |  |  |

Notes: Logistic regression models. DV = purchase of the focal brand. ^1^0: male, 1: female, ^2^0: no tablet ownership, 1: tablet ownership.

**Table Web-B2. Effects of AR-Controllability on Brand Image and Purchase Intention (Main Study)**

|  | **ARPI** | | | | | | | | |  |  | **Paper Booklet** | | | | | | | | |
| --- | --- | --- | --- | --- | --- | --- | --- | --- | --- | --- | --- | --- | --- | --- | --- | --- | --- | --- | --- | --- |
|  | Brand Image | | | |  | Purchase Intention | | | |  |  | Brand Image | | | |  | Purchase Intention | | | |
|  |  | β | t | p |  |  | β | t | p |  |  |  | β | t | p |  |  | β | t | p |
| *Controls* |  |  |  |  |  |  |  |  |  |  |  |  |  |  |  |  |  |  |  |  |
| Gender^1^ |  | -.007 | -.094 | .925 |  |  | .038 | .534 | .594 |  |  |  | .098 | 1.060 | .291 |  |  | .157 | 1.750 | .083 |
| Age |  | -.048 | -.700 | .485 |  |  | -.097 | -1.393 | .165 |  |  |  | .126 | 1.242 | .217 |  |  | .167 | 1.698 | .092 |
| Device experience^2^ |  | .063 | .936 | .350 |  |  | .031 | .457 | .648 |  |  |  | .004 | .038 | .969 |  |  | .240 | 2.475 | .015 |
| Product category knowledge |  | .126 | 1.718 | .087 |  |  | .021 | .290 | .772 |  |  |  | .003 | .027 | .978 |  |  | .226 | -2.112 | .037 |
| Sustainability attitude |  | .049 | .641 | .522 |  |  | .075 | .981 | .328 |  |  |  | .145 | 1.246 | .215 |  |  | .103 | .913 | .363 |
| *Treatment* |  |  |  |  |  |  |  |  |  |  |  |  |  |  |  |  |  |  |  |  |
| Controllability |  | .157 | 1.433 | .153 |  |  | .123 | 1.277 | .203 |  |  |  | .125 | 1.022 | .309 |  |  | .084 | .702 | .484 |
| Detailedness |  | .124 | 1.382 | .168 |  |  | **.191** | **2.121** | **.035** |  |  |  | .106 | .852 | .396 |  |  | .186 | 1.536 | .127 |
| **Controllability × detailedness** |  | **-.268** | **-2.299** | **.022** |  |  | **-.272** | **2.324** | **.021** |  |  |  | -.034 | .222 | .824 |  |  | -.080 | -.544 | .588 |
| R² |  |  | .050 |  |  |  |  | .046 |  |  |  |  |  | .088 |  |  |  |  | .133 |  |

Notes: OLS regression, β = standardized coefficients. ^1^0: male, 1: female, ^2^0: no tablet ownership, 1: tablet ownership.

# Web-Appendix C: Further Information about the Follow-up Study 1

**Figure Web-C1. Screenshots of ARPI-Videos (Follow-up Study 1)**

| **Shelf** | **Criteria** | **Statements** | **Arguments** |
| --- | --- | --- | --- |
| **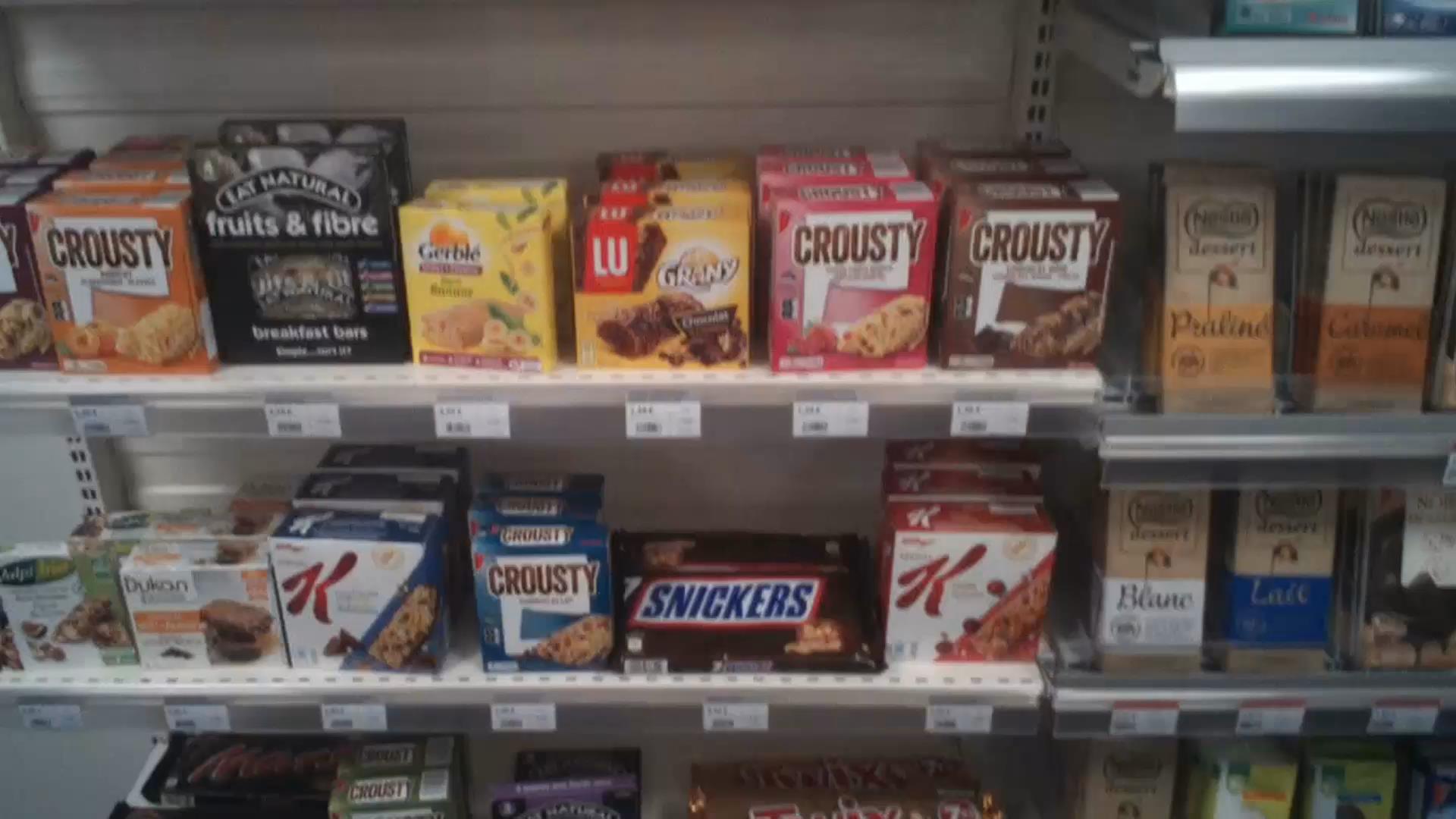** | **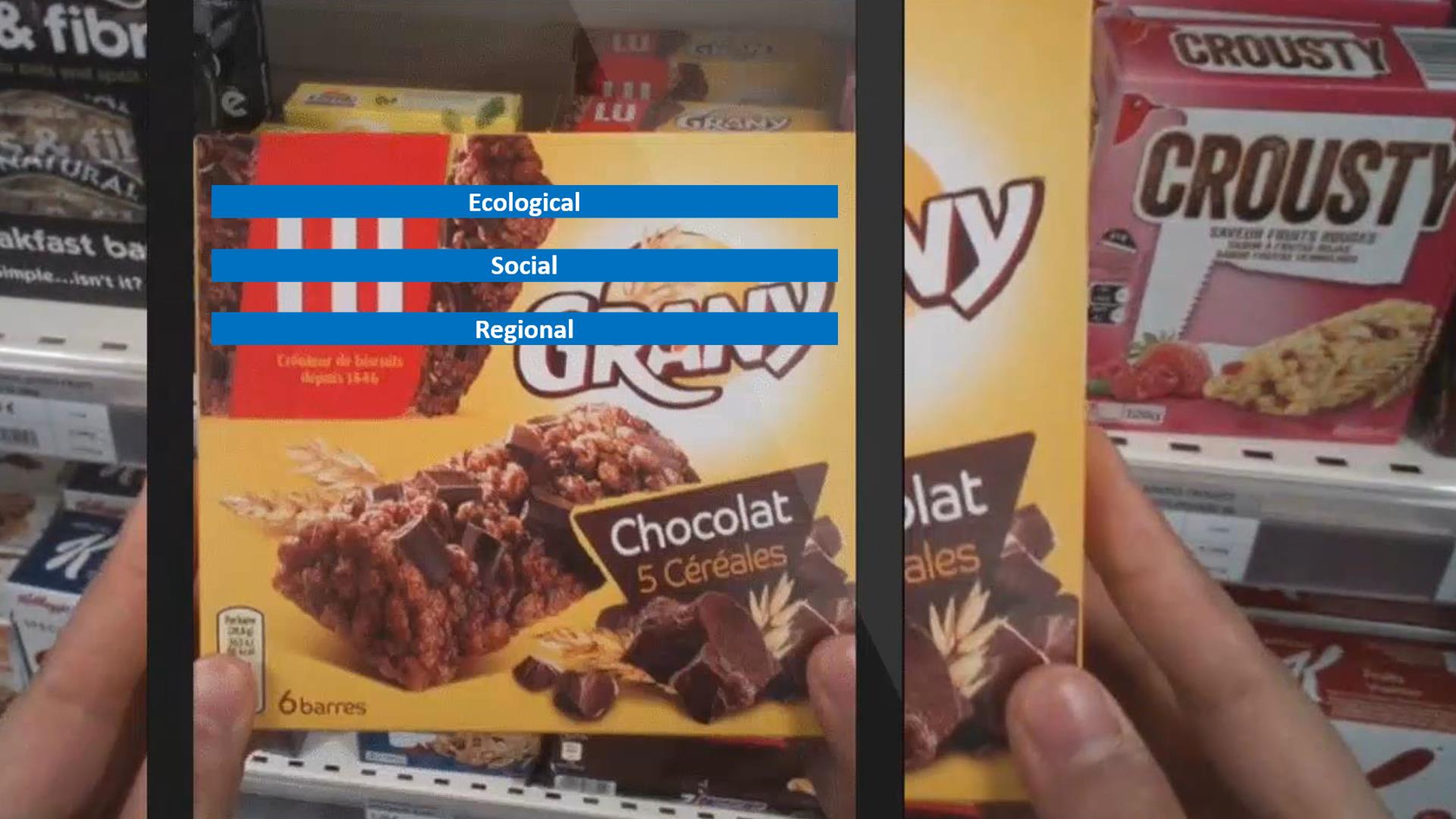** | **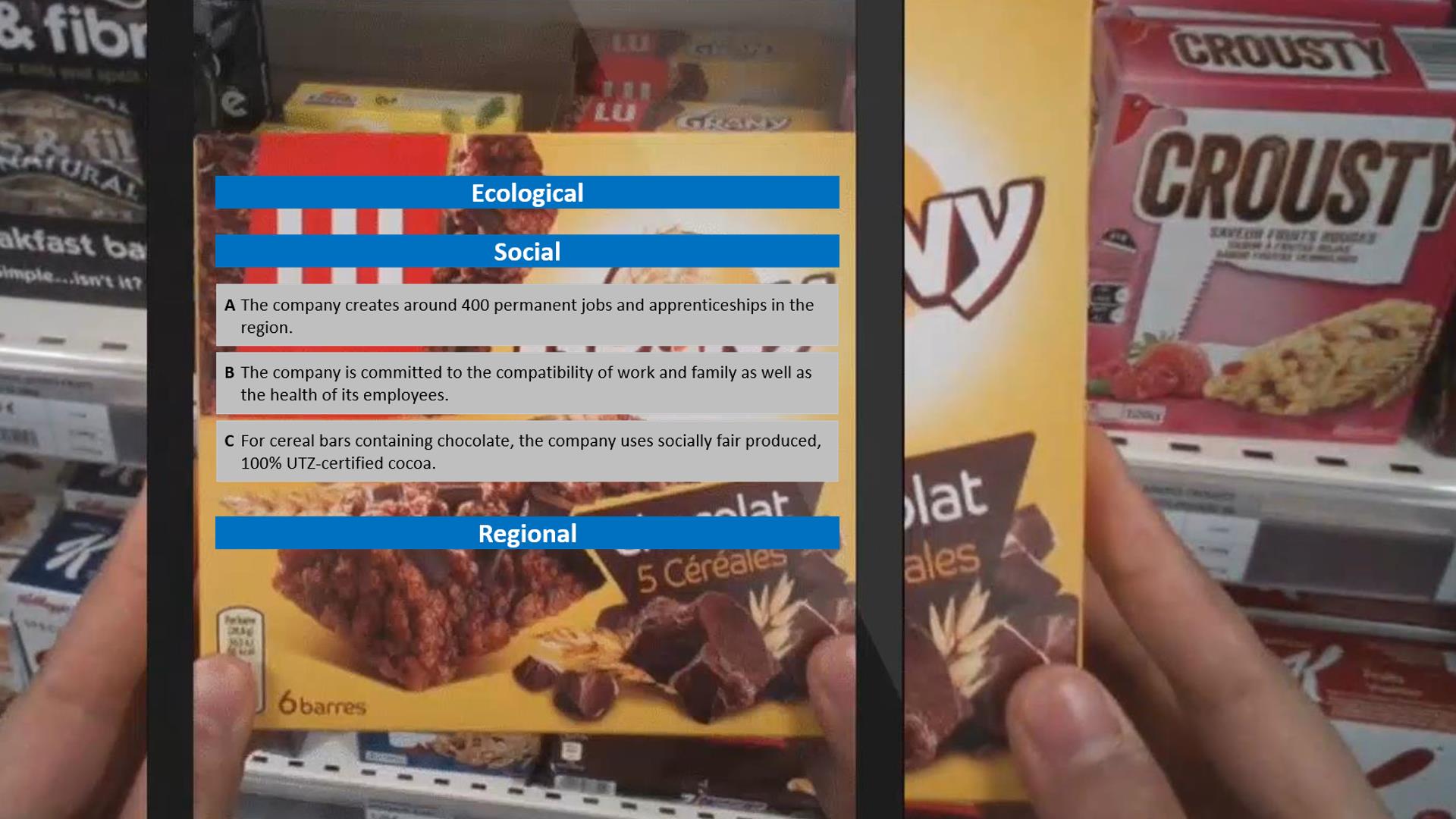** | **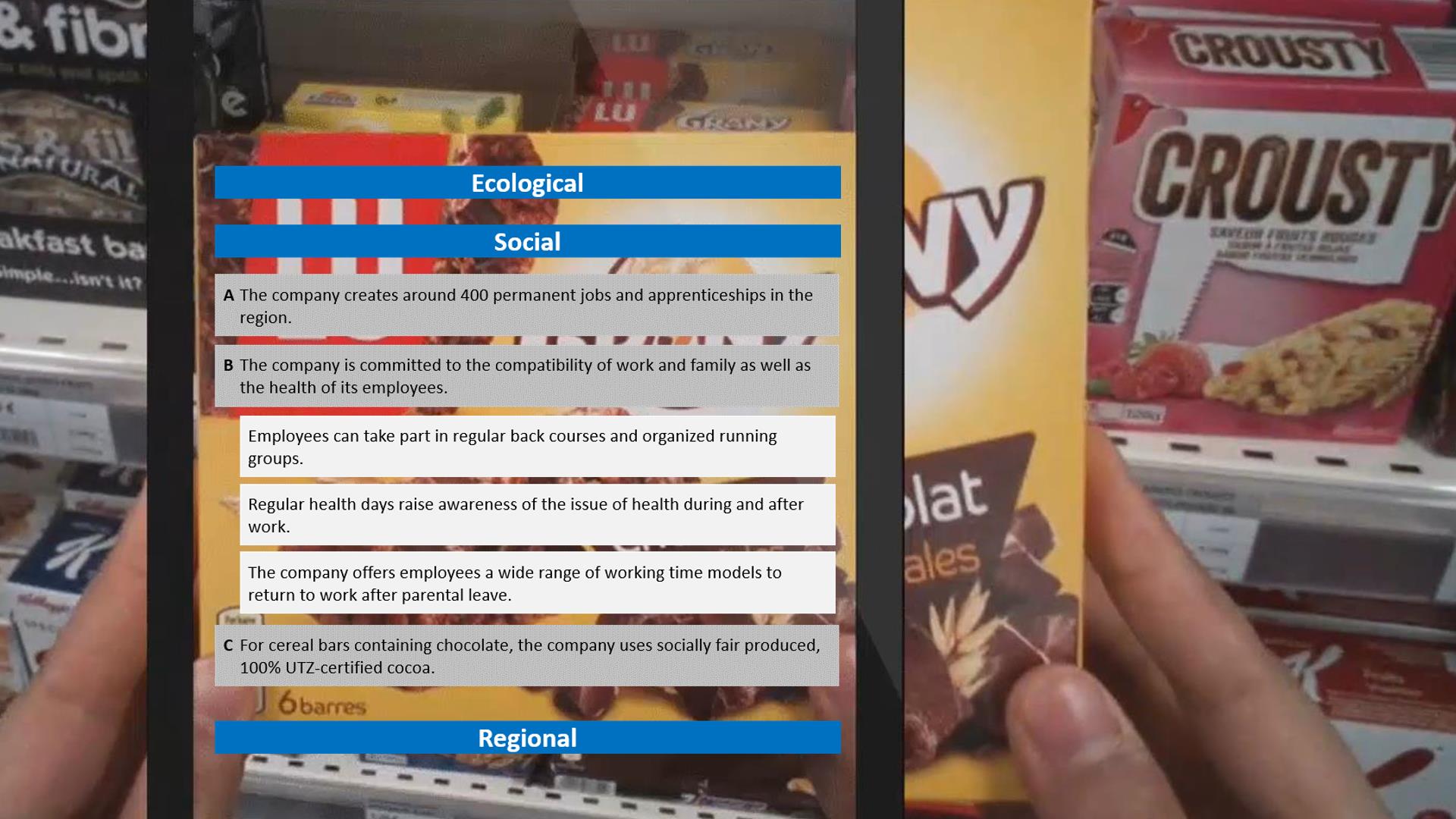** |

**Table Web-C1. Mediation analyses (Follow-up Study 1)**

| **Treatment** | **Mediator** | **Response** | **a (IV 🡪 MV)** | | |  | **b (MV 🡪 DV)** | | |  | **c (IV 🡪 DV)** | | | |  | **c' (IV 🡪 DV)** | | | |  | **a*b** | | | | | |
| --- | --- | --- | --- | --- | --- | --- | --- | --- | --- | --- | --- | --- | --- | --- | --- | --- | --- | --- | --- | --- | --- | --- | --- | --- | --- | --- |
|  |  |  | b | t | p |  | b | t | p |  | | b | t | p |  | | b | t | p |  | | b | LLCI | ULCI |  |  |
|  |  |  |  |  |  |  |  |  |  |  | |  |  |  |  | |  |  |  |  | |  |  |  |  |  |
| Controllability, 1 round | P. comprehensiveness | Brand Image | .371 | 2.122 | .035 |  | .396 | 9.423 | <.001 |  | | .265 | 1.832 | .068 |  | | .119 | .924 | .356 |  | | .161 | -.001 | .321 | † |  |
| Controllability, 3 rounds, no exit |  |  | .608 | 3.576 | <.001 |  |  |  |  |  | | .383 | 2.719 | .007 |  | | .143 | 1.126 | .261 |  | | .264 | .118 | .417 | ** |  |
| No ARPI |  |  | 1.159 | 6.422 | <.001 |  |  |  |  |  | | .476 | 3.184 | .002 |  | | .018 | .127 | .899 |  | | .503 | .345 | .671 | ** |  |
|  |  |  |  |  |  |  |  |  |  |  | |  |  |  |  | |  |  |  |  | |  |  |  |  |  |
| Controllability, 1 round | P. comprehensiveness | Purchase Intention | .371 | 2.122 | .035 |  | .413 | 5.920 | <.001 |  | | .869 | 3.889 | <.001 |  | | .716 | 3.354 | <.001 |  | | .107 | -.004 | .226 | † |  |
| Controllability, 3 rounds, no exit |  |  | .608 | 3.576 | <.001 |  |  |  |  |  | | .542 | 2.494 | .013 |  | | .291 | 1.383 | .168 |  | | .176 | .072 | .296 | ** |  |
| No ARPI |  |  | 1.159 | 6.422 | <.001 |  |  |  |  |  | | .826 | 3.582 | <.001 |  | | .348 | 1.492 | .137 |  | | .335 | .210 | .479 | ** |  |

Notes: Mediation analysis with PROCESS (model 4), Treatment (dummy-coded): baseline = controllability, 3 rounds, exit; mediator = perceived comprehensiveness. b = unstandardized coefficients; IV = controllability. Included control variables: gender, age, product category knowledge, sustainability attitude.. a: regression of the mediator on controllability in a single linear regression analysis; b: regression of DV on the mediator in a multiple regression analysis; c: regression of DV on controllability in a single linear regression analyses; c’: regression of DV on controllability in a multiple regression analysis; a*b: indirect effect; Bootstrapping (*CI-95%, 5,000 samples): LLCI: lower limit, ULCI: upper limit. Bootstrapping with CI-90% (†) and CI-99% (**) applied to test for the p < .10 and p < .01-level.

**Table Web-C2. MANCOVA – Effects of Treatment and Covariates (Follow-up Study 1)**

|  | **Overall effect** | | | | |  | **Brand image** | | |  | **Purchase intention** | |  | **Preference** | |  | **Comprehensiveness** | |
| --- | --- | --- | --- | --- | --- | --- | --- | --- | --- | --- | --- | --- | --- | --- | --- | --- | --- | --- |
|  | Wilks' Lambda | F | hypo. df | error df | p |  | F | p |  |  | F | p |  | F | p |  | F | p |
| Treatment | .828 | 4.88 | 12 | 791 | <.001 |  | 3.901 | .009 |  |  | 6.198 | <.001 |  | 2.090 | .102 |  | 14.578 | <.001 |
|  |  |  |  |  |  |  |  |  |  |  |  |  |  |  |  |  |  |  |
| *Covariates* |  |  |  |  |  |  |  |  |  |  |  |  |  |  |  |  |  |  |
| Sex | .996 | .30 | 4 | 299 | .881 |  | .342 | .559 |  |  | .021 | .884 |  | .238 | .626 |  | .927 | .336 |
| Age | .996 | .33 | 4 | 299 | .857 |  | .005 | .945 |  |  | .308 | .580 |  | .223 | .637 |  | .445 | .505 |
| Sustainability attitude | .927 | 5.90 | 4 | 299 | <.001 |  | 12.688 | <.001 |  |  | 13.438 | <.001 |  | 18.802 | <.001 |  | 1.555 | .213 |
| Category knowledge | .995 | .41 | 4 | 299 | .800 |  | .006 | .940 |  |  | .025 | .874 |  | .013 | .910 |  | 1.151 | .284 |

Notes: MANCOVA (Type III). Partial η² of treatment: Brand image: .037, purchase intention: .058, preference: .020, comprehensiveness: .126.

**Figure Web-C2. Means of the Dependent Variables and the Mediator Variable (Follow-up Study 1)**

# Web-Appendix D: Further Information about the Follow-up Study 2

**Table Web-D1. Experimental Design (Follow-up Study 2)**

| **Group** | **Treatment** | **Start** | **1^st^ Choice** | **Statements** | **2^nd^ Choice** | **Argument** |
| --- | --- | --- | --- | --- | --- | --- |
| 1 | non-controllable,  non-detailed | 3 boxes ecological, social, regional | none | three per box (successively) | none | none |
| 2 | non-controllable,  detailed | 3 boxes ecological, social, regional | none | three per box (successively) | none | three per statement (successively) |
| 3 | controllable,  non-detailed | 3 boxes ecological, social, regional | pick one box | three for  chosen box | none | none |
| 4 | controllable,  detailed | 3 boxes ecological, social, regional | pick one box | three for  chosen box | pick one statement | three for chosen statement |
| 5 | control group  (no ARPI) | no boxes | none | none | none | none |

**Table Web-D2. Contrasts against Control Group (Follow-up Study 2)**

|  | **DV: Brand Image** | | |  | **DV: Purchase intention** | | |  | **DV: Preference** | | |
| --- | --- | --- | --- | --- | --- | --- | --- | --- | --- | --- | --- |
|  | β | t | p |  | β | t | p |  | β | t | p |
| Sex^1^ | .039 | .772 | .440 |  | .025 | .487 | .627 |  | -.110 | -2.202 | .028 |
| Age | .013 | .268 | .788 |  | .047 | .925 | .355 |  | .098 | 1.993 | .047 |
| Education^2^ | -.037 | -.745 | .457 |  | -.065 | -1.277 | .202 |  | .023 | .455 | .649 |
| Sustainability attitude | .108 | 2.004 | .046 |  | .117 | 2.128 | .034 |  | .231 | 4.315 | <.001 |
| Product category knowledge | .072 | 1.352 | .177 |  | -.008 | -.144 | .886 |  | -.010 | -.185 | .853 |
|  |  |  |  |  |  |  |  |  |  |  |  |
| *Treatment (Baseline: CG)*^3^ |  |  |  |  |  |  |  |  |  |  |  |
| - uncontrollable/non-detailed | .211 | 3.584 | <.001 |  | .136 | 2.267 | .024 |  | .094 | 1.603 | .110 |
| - uncontrollable/detailed | .185 | 3.059 | .002 |  | .100 | 1.629 | .104 |  | .135 | 2.247 | .025 |
| - controllable/non-detailed | .148 | 2.388 | .017 |  | .075 | 1.190 | .235 |  | .085 | 1.374 | .170 |
| - controllable/detailed | .247 | 4.010 | <.001 |  | .170 | 2.709 | .007 |  | .141 | 2.302 | .022 |
|  |  |  |  |  |  |  |  |  |  |  |  |
| R² | .072 |  |  |  | .039 |  |  |  | .081 |  |  |
| R²adj | .052 |  |  |  | .018 |  |  |  | .061 |  |  |

Notes: OLS regression, β = standardized coefficients. ^1^0: male, 1: female and divers, ^2^0: no university degree, 1: university degree. ^3^treatment dummy-coded, control group without treatment as baseline.

**Table Web-D3. Interaction Effect of Controllability** × **Detailedness (Follow-up Study 2)**

|  | **DV: Brand Image** | | |  | **DV: Purchase intention** | | |  | **DV: Preference** | | |
| --- | --- | --- | --- | --- | --- | --- | --- | --- | --- | --- | --- |
|  | β | t | p |  | β | t | p |  | β | t | p |
| Sex^1^ | .045 | .806 | .421 |  | -.014 | -.245 | .807 |  | -.172 | -3.116 | .002 |
| Age | -.006 | -.113 | .910 |  | .038 | .676 | .499 |  | .091 | 1.673 | .095 |
| Education^2^ | -.056 | -.997 | .320 |  | -.070 | -1.244 | .214 |  | .008 | .147 | .883 |
| Sustainability attitude | .115 | 1.858 | .064 |  | .129 | 2.064 | .040 |  | .286 | 4.718 | <.001 |
| Product category knowledge | .133 | 2.188 | .029 |  | .081 | 1.327 | .186 |  | -.033 | -.558 | .578 |
|  |  |  |  |  |  |  |  |  |  |  |  |
| *Treatment* |  |  |  |  |  |  |  |  |  |  |  |
| - Controllability | -.119 | -1.504 | .133 |  | -.100 | -1.250 | .212 |  | -.024 | -.310 | .757 |
| - Detailedness | -.062 | -.762 | .446 |  | -.068 | -.832 | .406 |  | .034 | .437 | .662 |
| - Controllability × Detailedness | .173 | 1.786 | .075 |  | .166 | 1.697 | .091 |  | .024 | .256 | .798 |
|  |  |  |  |  |  |  |  |  |  |  |  |
| R² | .061 |  |  |  | .047 |  |  |  | .100 |  |  |
| R²adj | .038 |  |  |  | .023 |  |  |  | .078 |  |  |

Notes: OLS regression, β = standardized coefficients. ^1^0: male, 1: female and divers, ^2^0: no university degree, 1: university degree.

# Web-Appendix E: Further Information about the Follow-up Study 3

**Table Web-E1. Purchases based on ARPI Usage and Busy Shopping Times (Matched Control Group, Follow-up Study 3)**

|  | **Standard beer (pilsner)** | | | |  | **Other beer types** | | | |  | **Other (vs. standard) types** | | | |
| --- | --- | --- | --- | --- | --- | --- | --- | --- | --- | --- | --- | --- | --- | --- |
|  | ARPI  effect | SE | t | p |  | ARPI  effect | SE | t | p |  | ARPI  effect | SE | t | p |
| *Moderation by rush hour* |  |  |  |  |  |  |  |  |  |  |  |  |  |  |
| App use | -10.48 | 5.79 | -1.81 | .073 |  | 18.71 | 6.50 | 2.88 | .005 |  | 29.19 | 9.17 | 3.18 | .002 |
| Rush hour | -8.01 | 5.10 | -1.57 | .120 |  | 1.10 | 5.72 | .19 | .847 |  | 9.11 | 8.08 | 1.13 | .262 |
| App use × Rush hour | 11.41 | 6.98 | 1.64 | .105 |  | -14.41 | 7.83 | -1.84 | .069 |  | -25.83 | 11.05 | -2.34 | .021 |
| *Conditional effects* |  |  |  |  |  |  |  |  |  |  |  |  |  |  |
| Rush hour | +.93 | 3.79 | 0.24 | .812 |  | +4.29 | 4.37 | .98 | .328 |  | +3.36 | 6.16 | .55 | .586 |
| No rush hour | -10.48 | 5.79 | -1.81 | .073 |  | +18.71 | 6.50 | 2.88 | .005 |  | +29.19 | 9.17 | 3.18 | .002 |
| *Moderation by number of purchasing customers* |  |  |  |  |  |  |  |  |  |  |  |  |  |  |
| App use | -10.37 | 6.71 | -1.54 | .126 |  | 23.59 | 7.59 | 3.11 | .002 |  | 33.96 | 10.63 | 3.19 | .002 |
| Rush hour | -.38 | .23 | -1.70 | .093 |  | .36 | .25 | 1.40 | .165 |  | .74 | .36 | 2.07 | .041 |
| App use × Rush hour | .47 | .34 | 1.36 | .176 |  | -.83 | .39 | -2.15 | .034 |  | -1.30 | .54 | -2.39 | .019 |
| *Conditional effects* |  |  |  |  |  |  |  |  |  |  |  |  |  |  |
| 5 customers | -8.02 | 5.27 | -1.52 | .131 |  | +19.42 | 5.96 | 3.26 | .002 |  | +27.44 | 8.35 | 3.29 | .001 |
| 10 customers | -5.68 | 4.05 | -1.40 | .164 |  | +15.24 | 4.57 | 3.33 | .001 |  | +20.93 | 6.41 | 3.26 | .002 |
| 15 customers | -3.34 | 3.30 | -1.01 | .315 |  | +11.07 | 3.73 | 2.96 | .004 |  | +14.41 | 5.23 | 2.75 | .007 |
| 20 customers | -.99 | 3.37 | -.29 | .769 |  | +6.90 | 3.81 | 1.81 | .073 |  | +7.89 | 5.34 | 1.48 | .142 |
| 25 customers | +1.35 | 4.21 | .32 | .748 |  | +2.73 | 4.76 | .57 | .567 |  | +1.38 | 6.66 | .21 | .837 |
| 30 customers | +3.70 | 5.47 | .68 | .501 |  | -1.44 | 6.19 | -.23 | .816 |  | -5.14 | 8.67 | -.59 | .555 |

Notes. All consumers were matched from the days where the ARPI was tested in the market. Rush-hour: N = 51, no rush-hour: N = 51. Propensity score matching. Matching variables: amount of and expenditures on purchased food products (without beer), amount of and expenditures on non-alcoholic beverages, amount of and expenditures on alcoholic drinks (without beer), amount of and expenditures on coffee/tea, amount of and expenditures on sweets (incl. desserts), amount of and expenditures on vegetables and fruits, amount of and expenditures on fish products, amount of and expenditures on meat products, amount of and expenditures on grain products (incl. bread, noodles, cookies), number of purchasers (per 30 min), week and day when the app was used in the marked, time of the purchase, dummies for the week, dummies for the weekday.

**Figure Web-E1. ARPI-induced Shift in the Purchases of Standard vs. Other Beer Types under Different Specifications of Rush Hour (Follow-up Study 3)**

Notes. Upper panel: Contrasting relaxed shopping times vs. rush hours based on weekdays and day times.
Lower panel: Contrasting a few other shoppers in the aisle vs. many other shoppers.

# Web Appendix F: Follow-up Study 4: Experimental Manipulation of the Rush Hour

### Objective

In the previous studies reported in the paper, we have identified the rush hour as an important contextual variable that determines the effectiveness of ARPI. To capture this mechanism, we tested for various proxies, such as the daytime (main study) or the number of purchases in a given period (follow-up study 3). To provide further insights and evidence for the critical role of the rush hour, we manipulated this variable experimentally in follow-up study 4. In line with our observations in the main study and follow-up study 3, which were both conducted in a field setting, we expect that using the AR device in a rush hour has negative implications even for brand judgments and purchases.

### Design

The study was conducted in a fashion lab store of a French business school. In a two-level (rush hour vs. no rush hour) experiment, participants used an AR device to gain access to additional product information. As in the main study, the additional information was sustainability-related, but this time in the context of fashion. The fashion store is part of a behavioral lab that allows simulating customer journeys and consists of various shelves of sports fashion and related utensils, a cash register, etc. The experiment was embedded in a series of studies unrelated to the topic.

Upon arrival at the fashion store, participants were asked to imagine that they are looking for a piece of fashion in this store, which will be a present for a friend who is highly concerned about environmental issues. The instructions told participants that they would like to know more about the manufacturing of one sweater that was positioned on a stand (see Figure Web-F1). The sweater brand provides additional information on the fashion items and the labels, but this information was shown in another language (here German). To access this additional product information, the participants used a tablet with an augmented reality app that allows translations in real-time. The translated information occurred as soon as participants pointed the app on the text on the product. The instructions asked the participant to use the app to receive further information for making a better decision about their friend’s present. Like for the ARPI in the main experiment, the AR function thus showed the additional (translated) information directly on the product. This study zooms in on the moderating role of rush hour and keeps the factors controllability and detailedness constant.

To manipulate the rush hour condition, the participants were told in the beginning that they were “in a hurry because the next customer wants to look at the product too.” After 10 seconds, a screen showed a message that the next customer would like to look at the product too and, after another 10 seconds, a message told them that the next customer was pushing and that they had to hurry. After a total of 30 seconds, they were asked to put the product and tablet back on the table and complete a short questionnaire. In the non-rush condition, participants could use the AR app for 30 seconds to access the additional information on the product, but they were not told that other customers in the store would like to look at the product. Please note that this experiment happened during the COVID19 epidemic, in which social distancing was common in stores, and consumers were asked to keep distance from each other in retail areas. In total, 133 participants (mean age of 20.03 years, 53% females) completed the procedure. The participants indicated on a seven-point-scale no or very poor knowledge of German language (AM = 1.65, SD = 1.35, max = 5). Beyond sociodemographic information, the questionnaire contained the measures for perceived information comprehensiveness and brand image as in the previous studies. As the instructions told the participants that they intended to purchase the sweater, we used their willingness to pay as a purchase variable.

### Results

As a manipulation check, we assessed participant’s perceived rush (“Please, rate how looking at products like this made you feel” not stressful—stressful), which confirms successful manipulation of the rush hour condition (M_no rush_ = 2.61; M_rush_ = 4.42, t = 5.72, *p* < .001). We also observed a wide variance in the perceptions of the comprehensiveness of information that was accessed through the AR device, ranging from very low (1) to very high (6.5) with a mean at the midpoint of the scale (4.00, SD = 1.01). These perceptions did not differ across both conditions (T = .992, p = .323), which allows contrasting the conditional impact of AR use in rush hour on our marketing-related outcomes (the estimation results are presented in Table Web-F1).

**Table Web-F1. The Effect of Rush Hour in AR Use Depending on the Perceived Amount of Information Available (Follow-up Study F)**

|  | **Willingness to pay** | | | |  | **Willingness to pay (ranks)** | | | |  | **Brand image** | | | |
| --- | --- | --- | --- | --- | --- | --- | --- | --- | --- | --- | --- | --- | --- | --- |
|  | B | SE | t | p |  | B | SE | t | p |  | B | SE | t | p |
| Constant | 41.05 | 34.51 | 1.19 | .118 |  | 20.84 | 20.06 | 1.04 | .150 |  | 2.54 | .44 | 5.77 | <.001 |
| Rush hour | 64.92 | 46.86 | 1.39 | .084 |  | 42.20 | 27.24 | 1.55 | .062 |  | 1.46 | .60 | 2.44 | .008 |
| Information compre­hensiveness (IC) | 18.40 | 8.57 | 2.15 | .017 |  | 12.71 | 4.98 | 2.55 | .006 |  | .66 | .11 | 6.03 | <.001 |
| Rush hour × IC | -19.34 | 11.39 | -1.70 | .046 |  | -12.64 | 6.62 | -1.91 | .029 |  | -.34 | .15 | -2.32 | .011 |
| *Conditional effects of rush hour at different levels of perceived information comprehensiveness (IC)* | | | | | | | | | | | | | | |
| Low IC (3) | 6.89 | 15.99 | .43 | .333 |  | 4.29 | 9.30 | .46 | .323 |  | .45 | .20 | 2.19 | .015 |
| Moderate IC (4) | -12.45 | 11.31 | -1.10 | .137 |  | -8.34 | 6.57 | -1.27 | .104 |  | .11 | .14 | .77 | .223 |
| High IC (5) | -31.80 | 16.11 | -1.97 | .025 |  | -20.98 | 9.36 | -2.24 | .027 |  | -.23 | .21 | -1.10 | .137 |

Note. Level of significance (one-tailed).

For the willingness to pay, we observe a negative shift for AR-use being in a rush with greater perceptions of information comprehensiveness. Rush hour does not affect willingness to pay when the accessible information is perceived as less comprehensive (B_-1SD_ = 6.89, t = .43, p = .333). However, AR use under rush reduces willingness to pay by around €32 when information comprehensiveness is perceived as high (B_+1SD_ = -31.80, t = -1.97, p = .025). Results remained stable when we ran several robustness checks (see Table Web-F2). Likewise, we observed a similar negative shift for brand attitudes with elevating perceptions of information comprehensiveness. For this marketing outcome, AR use in a rush has positive implications when the accessible information accessed through the AR device is perceived to be less comprehensive (B_-1SD_ = .45, t = 2.19, *p* = .030). However, this advantage disappears when this information is perceived to be moderate (B_mean_ = .11, t = .77, *p* = .446) or very comprehensive (B_+1SD_ = -.23, t = -1.10, *p* = .273).

**Table Web-F2. Check for Extreme Values and Control Variables**

|  | **Willingness to pay^1^ (extreme cases removed)** | | | |  | **Willingness to pay  (90% winsorization)** | | | |  | **Willingness to pay  (90% winsorization)** | | | |
| --- | --- | --- | --- | --- | --- | --- | --- | --- | --- | --- | --- | --- | --- | --- |
|  | B | SE | t | p |  | B | SE | t | p |  | B | SE | t | p |
| Constant | 47.42 | 22.39 | 2.12 | .018 |  | 45.46 | 29.08 | 1.56 | .060 |  | -24.17 | 128.83 | -.19 | .425 |
| Rush hour | 49.92 | 30.44 | 1.64 | .052 |  | 53.06 | 39.48 | 1.34 | .091 |  | 55.20 | 40.16 | 1.37 | .086 |
| Information compre­hensiveness (IC) | 13.48 | 5.58 | 2.42 | .009 |  | 16.33 | 7.22 | 2.26 | .013 |  | 16.49 | 7.34 | 2.25 | .013 |
| Rush hour × IC | -16.90 | 7.45 | -2.27 | .013 |  | -15.97 | 9.60 | -1.66 | .049 |  | -16.37 | 9.74 | -1.68 | .048 |
| *Controls* |  |  |  |  |  |  |  |  |  |  |  |  |  |  |
| Age |  |  |  |  |  |  |  |  |  |  | 1.45 | 6.15 | .24 | .407 |
| Gender^2^ |  |  |  |  |  |  |  |  |  |  | -.36 | 10.08 | -.04 | .486 |
| Familiarity with app |  |  |  |  |  |  |  |  |  |  | 1.63 | 1.44 | 1.13 | .130 |
| Sustainability attitude |  |  |  |  |  |  |  |  |  |  | 4.38 | 4.65 | .94 | .174 |
| Product category knowledge |  |  |  |  |  |  |  |  |  |  | 3.46 | 3.04 | 1.14 | .129 |
| *Conditional effects of rush hour at different levels of perceived information comprehensiveness (IC)* | | | | | | | | | | | | | | |
| Low IC (3) | -.77 | 10.25 | -.08 | .471 |  | 5.15 | 13.47 | .38 | .352 |  | 6.08 | 13.76 | .44 | .330 |
| Moderate IC (4) | -17.67 | 7.29 | -2.42 | .009 |  | -10.82 | 9.53 | -1.14 | .129 |  | -10.29 | 9.72 | -1.06 | .146 |
| High IC (5) | -34.57 | 10.60 | -3.26 | <.001 |  | -26.79 | 13.57 | -1.97 | .026 |  | -26.67 | 13.77 | -1.94 | .028 |

Note. Level of significance (one-tailed). ^1^ participants excluded who indicated unreasonably low (< €25) or high (> €190) willingness to pay (N = 112). ^2^ one participant did not indicate the gender (N = 132).

### Discussion

This follow-up lab experiment confirmed the crucial role of rush hour as a context factor for AR use to obtain additional information. While, thus far, we have tapped this important context variable through proxies, we manipulated participants’ experienced rush in this study. Confirming our findings from the field setting in the main study and follow-up study 3, this lab study confirms that AR use in rush hour hampers marketing-related outcomes.

**Figure Web-F1. Stand in the Fashion Store and Focal Product**
